# Long read isoform sequencing reveals hidden transcriptional complexity between cattle subspecies

**DOI:** 10.1186/s12864-023-09212-9

**Published:** 2023-03-13

**Authors:** Yan Ren, Elizabeth Tseng, Timothy P. L. Smith, Stefan Hiendleder, John L. Williams, Wai Yee Low

**Affiliations:** 1 The Davies Research Centre, School of Animal and Veterinary Sciences, University of Adelaide, Roseworthy, Adelaide, SA 5371 Australia; 2 Pacific Biosciences, Menlo Park, CA USA; 3 U.S. Meat Animal Research Center, USDA-ARS, Clay Center, Clay Center, Nebraska USA; 4 Robinson Research Institute, The University of Adelaide, North Adelaide, Adelaide, SA 5006 Australia; 5 Department of Animal Science, Food and Nutrition, Università Cattolica del Sacro Cuore, 29122 Piacenza, Italy

**Keywords:** Iso-Seq, RNA-seq, Cattle, Differential Isoform expression, Transcriptome, Multi-mapped reads, Sequence duplication, Subspecies, Alternative splicing, Long read sequencing

## Abstract

The Iso-Seq method of full-length cDNA sequencing is suitable to quantify differentially expressed genes (DEGs), transcripts (DETs) and transcript usage (DTU). However, the higher cost of Iso-Seq relative to RNA-seq has limited the comparison of both methods. Transcript abundance estimated by RNA-seq and deep Iso-Seq data for fetal liver from two cattle subspecies were compared to evaluate concordance. Inter-sample correlation of gene- and transcript-level abundance was higher within technology than between technologies. Identification of DEGs between the cattle subspecies depended on sequencing method with only 44 genes identified by both that included 6 novel genes annotated by Iso-Seq. There was a pronounced difference between Iso-Seq and RNA-seq results at transcript-level wherein Iso-Seq revealed several magnitudes more transcript abundance and usage differences between subspecies. Factors influencing DEG identification included size selection during Iso-Seq library preparation, average transcript abundance, multi-mapping of RNA-seq reads to the reference genome, and overlapping coordinates of genes. Some DEGs called by RNA-seq alone appear to be sequence duplication artifacts. Among the 44 DEGs identified by both technologies some play a role in immune system, thyroid function and cell growth. Iso-Seq revealed hidden transcriptional complexity in DEGs, DETs and DTU genes between cattle subspecies previously missed by RNA-seq.

**Supplementary Information:**

The online version contains supplementary material available at 10.1186/s12864-023-09212-9.

## Introduction

RNA-seq has greatly advanced our understanding of the transcriptome in many species, however, it does not accurately resolve transcript structures from start to end, and the gene expression level estimates derived from this technology vary widely depending on the choice of analysis tools [1]. Most studies of transcript abundance by RNA-seq use 30 – 60 million RNA-seq reads [2], but even at this depth accurate assembly of transcripts to develop a complete picture of gene and isoform abundance is difficult (Gonzalez-Garay 2015). Pacific Biosciences (PacBio) long read sequencing provides full-length, single-molecule RNA sequence, termed Iso-Seq, which does not need transcript assembly and can improve gene annotation [3–6], even for well-characterized species including human and mouse [7–9]. Iso-Seq has also been used to study RNA editing sites [10], such as A-to-I modifications that have a role in the immune system and to diversify the transcriptome [11]. Additionally, long read transcripts can be incorporated to proteomics pipelines to better characterize protein isoform diversity [12]. A SMRT cell on Sequel II system produces on average 5 million Circular Consensus Sequencing (CCS) reads, which translates to fewer transcripts than RNA-seq for a given cost. Currently Iso-Seq is an order of magnitude more expensive than RNA-seq and few studies have compared Iso-Seq and RNA-seq to quantifying gene expression [8, 13, 14].

This study examined the correlation between RNA-seq and Iso-Seq estimations of relative transcript abundance and their predictions of differential gene expression. The correlation was tested in a situation where substantial numbers of DEGs might be anticipated, specifically using a set of samples of fetal livers from the two subspecies of domesticated cattle, *Bos indicus* and *Bos taurus*, that are genetically and phenotypically distinct [15]. Taurine cattle have been intensively selected for high milk production and beef production. However, their production potential is limited e.g. by heat stress [16]. In contrast, indicine cattle are less productive but well adapted to hotter environments, and are generally more disease- and parasite-tolerant [17, 18].

Phenotypic differences between the cattle subspecies are observed during fetal development at mid-gestation (~ day 150) [19]. These differences include larger bone size of taurine cattle when compared to indicine cattle [20] leading to higher birth weight. To study the molecular mechanisms underlying the phenotypic differences occurring during fetal development, we have recently used RNA-seq to profile gene expression differences in liver, brain, lung, muscle and placenta between representatives of taurine (Angus) cattle and indicine (Brahman) cattle [21]. A total of 110 genes were identified as differentially expressed between the two subspecies across all five tissues at mid fetal development. In particular, the expression differences in liver are high between the two subspecies, with 328 differentially expressed genes (DEGs) found between Brahman and Angus. We have reported the use of PacBio Iso-Seq to detect transcripts that would have otherwise been missed by RNA-seq [6], however, due to lack of biological replicates, the study was primarily focused on annotating the transcriptome.

In this study, we compared the expression of genes and transcripts between taurine and indicine cattle to gain biological insights into expression differences at a critical time point in fetal development. We profiled the liver tissue of Brahman and Angus fetuses at day 153 with both Iso-Seq and RNA-seq using the same biological samples to compare the technologies and investigate the utility of long reads in quantifying gene and transcript abundance, and determining the effect of platform on identifying differentially expressed genes between samples.

## Materials and methods

### Sample collection

All animal experiments and procedures described in this study were compliant with Australian guidelines, approved by the University of Adelaide’s Animal Ethics Committee and followed the ARRIVE Guidelines (https://arriveguidelines.org/) (Approval No. S-094–2005). The Brahman and Angus conception were generated as previously described [22]. Fetuses were recovered at day 153 ± 1 of gestation after dams were sacrificed in an abattoir and the fetal liver samples (*Lobus hepaticus sinister*) snap frozen in liquid nitrogen and stored at -80 °C until further use. Day 153 of gestation was chosen based on the fact that the bovine fetus enters the accelerated growth phase at this time. The gestation length in cattle is similar to humans. In addition, the growth trajectory and development in cattle also appear to be surprisingly similar to humans. A subset of the liver samples analysed in our previous work [21] was used for this study, which consists of three female Brahman (*Bos indicus*) and three female Angus (*Bos taurus*) samples.

### RNA-seq data generation and pre-processing

RNA was extracted from the tissue and prepared for Illumina RNA-seq short-read sequencing and PacBio Iso-Seq long-read sequencing. The RNA-seq protocol and data availability (GEO accession number: GSE148909) were previously described [21]. The target was to produce 50 million 100 bp paired-end reads per sample on a NextSeq 500 Illumina platform. Initially the quality of raw RNA-seq reads was checked using FASTQC v0.11.4, then TrimGalore v0.4.2 [23] was used to trim the reads and the Phred score of 10 was set. Sequencing adapters and reads shorter than 100 bp were removed by AdapterRemoval v2.2.1 [24]. The cleaned reads were checked by FASTQC again. Using Hisat2 v2.1.0 [25], the cleaned reads were mapped to the Brahman Ensembl v104 reference genome [6] (GenBank accession no GCF_003369695.1). The mapping rate was over 80% for all samples. Mapped reads were sorted using SAMtools v1.8 [26].

The analyses were conducted separately at the level of genes and transcripts (Fig. S1). At the gene level, FeatureCounts v1.5.2 [27] was used to count RNA-seq reads mapped in genes. For the transcript level, Kallisto v0.48.0 [28] was used to quantify abundances of transcripts based on pseudoalignments. gffcompare v0.11.2 [29] was used to combine annotations from PacBio transcripts found in this study with those of Brahman from Ensembl v104 to optimize the assignment of RNA-seq reads. Using the combined annotations and Kallisto, we obtained transcripts per kilobase million (TPM).

### PacBio Iso-Seq long read data pre-processing

RNA was extracted from the same female liver samples used for RNA-seq and processed into sequencing libraries using the SMRTbell Express Template Prep Kit 2.0 and Iso-Seq Express 2.0 Workflow as per the manufacturer’s standard protocol with 15 cycles of PCR and 86 µL of ProNex beads for size selection (Pacific Biosciences, Menlo Park CA). The procedure includes amplification (15 cycles) of complementary DNA (cDNA) and bead-based size selection (86 µL of ProNex beads) during library preparation. Each of the six libraries were sequenced on two SMRT cells on a Sequel II instrument loaded at 100 pM on-plate concentration. Data from each pair of SMRT cells (12 SMRT cells total; two for each sample) were pooled and run through the Iso-Seq analysis in SMRTLink v8.0 to produce high-quality, full-length transcripts. Briefly, the Iso-Seq process involved assignment of molecules to Full-length (FL) reads and Non-FL reads. A clustering analysis was then done to group FL reads at the transcript level. Non-FL and FL reads were used to generate consensus reads for each transcript. The high-quality full-length transcripts were mapped to the Brahman reference genome [6] (GenBank accession no GCF_003369695.1) using minimap2 v2.17. Redundant and degraded isoforms were collapsed using Cupcake v17.1.0 as per the protocol described in GitHub (https://github.com/Magdoll/cDNA_Cupcake/wiki/Cupcake:-supporting-scripts-for-Iso-Seq-after-clustering-step#collapse). The non-redundant isoforms were filtered and classified using the Brahman transcriptome annotation, Ensembl v104 and SQANTI3 [30].

### Differential gene expression

The counts per gene of RNA-seq short read data were first filtered for a minimum count per million (CPM) > 0.5 in at least three samples. Then, the counts were normalized using the trimmed mean of M values (TMM) method [31], where the M-values were weighted according to inverse variances by default. The limma v3.44.3 [32] linear model was used to compare the expression levels between Brahman and Angus samples using the gene counts. The *p*-values were adjusted by false discovery rate (FDR) to correct for multiple testing. Differentially expressed genes were found after filtering for FDR < 0.05 and log fold change > 1. The R package sleuth v 0.30.0 [33] was used for differential gene expression analysis of Kallisto outputs, to aggregate the transcripts at the gene-level.

Differential gene expression analysis for Iso-Seq data was based on the counts of transcripts that were transcribed from the same gene. Each gene was then filtered for count per million (CPM) > 0.5 in at least three samples. The counts per gene were normalized using the TMM method [31]. The limma linear model was used to compare the expression levels between pure Brahman and pure Angus. The results were filtered for FDR < 0.05 and log fold change > 1.

### DEG discrepancy analysis

The Wilcoxon rank sum test [34] was used to test whether there was any difference in average expression values between DEGs found by RNA-seq alone and both RNA-seq and Iso-Seq. Multi-mapping of RNA-seq reads in DEGs was identified using the “NH” tag in bam files. Specifically, in the “NH:i:x” tag, if x > 1, indicated multi-mapping of a read. For each DEG per sample, the level of multi-mapping was defined as: (the number of multi-mapped reads / total number of mapped reads) × 100%.

For each DEG, the percentage of overlap with other genes was calculated as: (length of overlapped positions with other genes / length of DEG) × 100%.

### Differential transcript analysis

Differential transcript analysis of the RNA-seq data was conducted with TPM values from Kallisto using sleuth in R. We required TPM > 1 in at least three samples to consider the transcripts for further analysis. Transcripts were normalized using median ratio normalization before running sleuth. To test for transcripts that were differentially expressed between the subspecies, both “full” and “reduced” measurement error models were fitted. The software sleuth first fitted a “reduced” model that presumed transcript abundances were unaffected by cattle subspecies. Then it performed a “full” model that considered the cattle subspecies as an explanatory variable. A likelihood ratio test between the two fitted models revealed transcripts where there was a significant subspecies effect.

The Iso-Seq pipeline produced the count of transcripts which were filtered for CPM > 0.5 in at least three samples. The counts per transcript were normalized using TMM. The limma v3.44.3 linear model was used to compare the expression levels of genes and transcripts between pure Brahman and pure Angus. The results were filtered for FDR < 0.05 and log fold change > 1.

### Transcriptome characterization

The Iso-Seq transcripts were categorized into four major groups by SQANTI3: Full Splice Match (FSM), Incomplete Splice Match (ISM), Novel in Catalog (NIC), and Novel Not in Catalog (NNC). For RNA-seq data we classified the transcripts into two categories: 1) ‘Known’ transcripts that matched transcripts in the Ensembl Brahman annotation identified by Kallisto. 2) ‘Novel’ transcripts matching Iso-Seq-defined novel transcripts identified by Kallisto.

The Coding Potential Calculator v2.0.0 (CPC2) [35] was used to identify the open reading frames (ORFs) and predict protein coding potential of novel transcripts.

### Gene and transcript expression correlation analyses

The correlation of gene expression between RNA-seq and Iso-Seq was determined by calculation of the Pearson correlation coefficient for the counts from FeatureCounts for RNA-seq data and the counts from SQANTI3 for Iso-Seq data.

At the transcript level, Pearson correlation analysis was done using values obtained from Kallisto for RNA-seq and SQANTI3 for Iso-Seq. For RNA-seq, the TPM values of expressed transcripts (TPM > 1 in at least three samples) from Kallisto were used for correlation analyses. For Iso-Seq, the counts of expressed transcripts for each sample were first converted to TPM values and then filtered by TPM > 1 in at least three samples.

### Differential transcript usage analysis

Differential transcript usage analysis was applied to the RNA-seq based transcriptome identified by Kallisto using the R package DRIMSeq v1.18.0 [36]. Data were filtered by TPM.filter with TPM > 1 in at least three samples. The maximum likelihood method was used to calculate the precision parameter in the Dirichlet-multinomial model used for the differential transcript usage analysis. The data were fitted for “null” and “full” models; the difference between the two models was “full” had cattle subspecies as an explanatory variable. Likelihood ratio tests were performed to detect differential transcript usage.

The R package, DRIMSeq v1.18.0, was used for differential transcript usage analysis of the Iso-Seq data. The Iso-Seq data were filtered for at least 10 counts in each transcript in at least 3 samples using the function dmFilter() before running the maximum likelihood method, which was used to detect differential transcript usage described for RNA-seq data. The structure of transcripts was shown using ggtranscript [37].

### Pathway analysis

DEGs and DTU genes that were found by more than one method were used as inputs for GO pathway analysis using g:Profiler [38].

## Results

### Summary statistics of RNA-seq and Iso-Seq

The depth of RNA-seq sequencing (Table 1; average 60 million 100 bp paired-end raw reads per sample, range 45–103 million) was sufficient to detect alternative splicing variants genome wide. Approximately 95% of the reads were successfully aligned to the reference genome, and ~ 75% of these mapped reads could be assigned to at least one gene by FeatureCounts.

**Table 1:** Mapping statistics of RNA-seq and Iso-Seq. The % of mapped reads and assigned genes were calculated based on cleaned reads

Sample	RNA-seq raw reads	RNA-seq cleaned reads	RNA-seq mapped reads (%)	RNA-seq assigned to genes (%)	Iso-seq CCS reads	Iso-seq FLNC reads
Angus_7	49,189,375	47,752,086	95.66%	75.70%	8,840,157	8,124,187
Angus_53	51,315,712	50,634,082	94.88%	76.30%	8,730,907	7,893,212
Angus_60	103,506,419	101,100,269	95.97%	76.80%	8,388,042	7,759,609
Brahman_65	45,816,738	44,996,438	95.26%	76.90%	8,578,336	7,631,806
Brahman_22	53,889,070	53,018,406	94.99%	76.30%	10,983,128	9,340,386
Brahman_99	57,939,684	56,934,361	95.94%	77.60%	10,217,439	8,404,511

Depth of sequencing for Iso-Seq was lower than the RNA-seq, with between 8,388,042 and 10,983,128 circular consensus sequence (CCS) reads, with an average of ~ 28 billion bases of CCS reads per sample (Table 1). Read corrections and removal of adapters and poly-A tails left an average of 8,192,285 Full Length Non-Chimeric (FLNC) reads per sample with an average FLNC length of 2,902 bases. Rarefaction curves of Iso-Seq sequencing depths reached a plateau at the gene-level and approached a plateau at the transcript level indicating sufficient depth to identify most expressed isoforms (Fig. S2).

### Transcriptome characterization

The analysis of unique isoforms derived from Iso-Seq data identified 54,692 novel transcripts (81.7%) and 12,247 known transcripts. Over 90% of the novel transcripts were predicted to be protein-coding with an average ORF length of 492 nucleotides. Approximately 1.9% of the identified 12,765 genes (237 genes) were novel genes i.e. not found in the Ensembl annotation. About 45% of identified genes had more than 5 isoforms, and genes with multiple isoforms were more likely to be found as annotated genes (Fig. S3A and B). Classification of transcripts in comparison to annotated splice forms indicated that 23.9% transcripts were FSM i.e. matching a reference transcript at all splice junctions whereas 27.6% transcripts were ISM i.e. partially matching a reference transcript at splice junctions. Novel transcripts that aligned with known genes but used either novel splice donor and/or acceptors (class NNC) accounted for 25.7% of the transcripts, whereas 20.8% of the transcripts contained new combinations of already annotated splice junctions (class NIC) (Fig. S4A). The longer transcripts largely fitted into either the NIC and NCC categories (Fig. S4B).

The majority of transcripts (~ 86%) identified by Kallisto from RNA-seq reads were predicted to be protein-coding with an average ORF length of 1,146 nucleotides. These transcripts were compared to those identified by Iso-Seq to determine the overlap of novel transcript identification between approaches. This identified 20,529 novel transcripts (27.11% of total RNA-seq defined transcripts) in addition to 55,485 known transcripts.

### Gene and transcript expression and correlation

The FLNC transcripts identified by Iso-Seq included 66,939 unique transcripts corresponding with 12,765 genes. In contrast, we identified 16,386 genes with FeatureCounts and 76,024 transcripts with Kallisto using short RNA-seq reads. Genes that were identified by RNA-seq but missed by Iso-Seq tend to have lower counts i.e. lowly expressed genes. At the gene-level, among the genes with detectable expression, there were 11,960 (70.6%) genes that overlapped between RNA-seq and Iso-Seq. At the transcript-level, there were 12,329 transcripts (9.4%) that overlapped between the two sequencing methods.

**Fig. 1:**
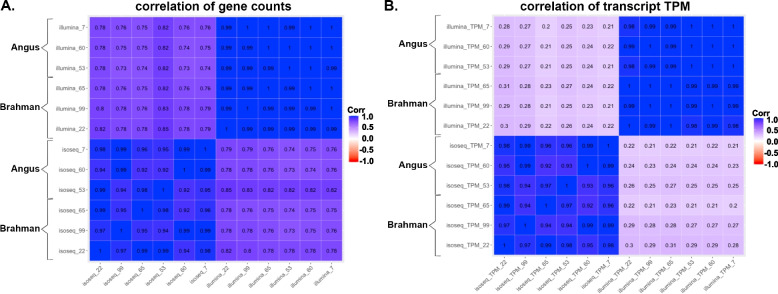
Iso-Seq sequencing correlation of gene/transcript expression with RNA-seq. **A** Correlation of gene expression using the counts from Iso-Seq SQANTI3 and RNA-seq FeatureCounts. **B** Correlation of transcript expression using the TPM from Iso-Seq SQANTI3 and RNA-seq Kallisto. Notes: Samples with names ending with 7, 60, and 53 are individual Angus samples whereas samples with names ending with 65, 99, and 22 are individual Brahman samples

The overall correlation of expression for all pairwise comparisons at the gene-level (range 0.7–1, Fig. 1A) was higher than at transcript-level using TPM (range 0.2–1, Fig. 1B). Higher correlations of gene and transcript expression were observed within individual technology (i.e. within RNA-seq or within Iso-Seq) between samples rather than across technologies but within sample. The correlations among samples within technology were 0.98 for the RNA-seq at both gene- and transcript-levels, whereas correlation was slightly lower for Iso-Seq, but still above 0.92 for all pairwise correlations.

### Differential gene and transcript expression

There were 168 DEGs between fetal livers of taurine and indicine cattle identified from Iso-Seq CCS reads, including 67 transcripts with increased abundance and 101 with decreased abundance in Angus compared to Brahman (Table S1). In contrast, 283 DEGs were identified from RNAs-eq data (68% higher than from CCS data), of which 120 and 163 genes had higher and lower abundance, respectively, in Angus fetal livers (Fig. S1; Table S2). Alternate estimates based on TPM values from RNA-seq data via sleuth and Kallisto identified fewer (141) DEGs including 69 and 72 genes with increased and decreased expression in Angus, respectively (Table S3).

The comparison of Iso-Seq and RNA-seq analyses at the level of DETs sharply contrasted with results at the gene level. There were 184 transcripts that were differentially expressed when analysed with Iso-Seq counts, of which 64 had higher frequency and 120 had lower frequency in Angus (Table S4). In contrast, only 10 transcripts were identified as differentially expressed in the RNA-seq data using TPM values from Kallisto. The frequency of two of these transcripts were increased whereas eight transcripts decreased in Angus (Table S5). The 18-fold lower number of DETs detected by RNA-seq probably reflects the limitations of using short reads to accurately identify specific transcripts, in contrast to the ability to quantify abundance at the gene level.

### Overlap of DEGs and DETs

**Fig. 2:**
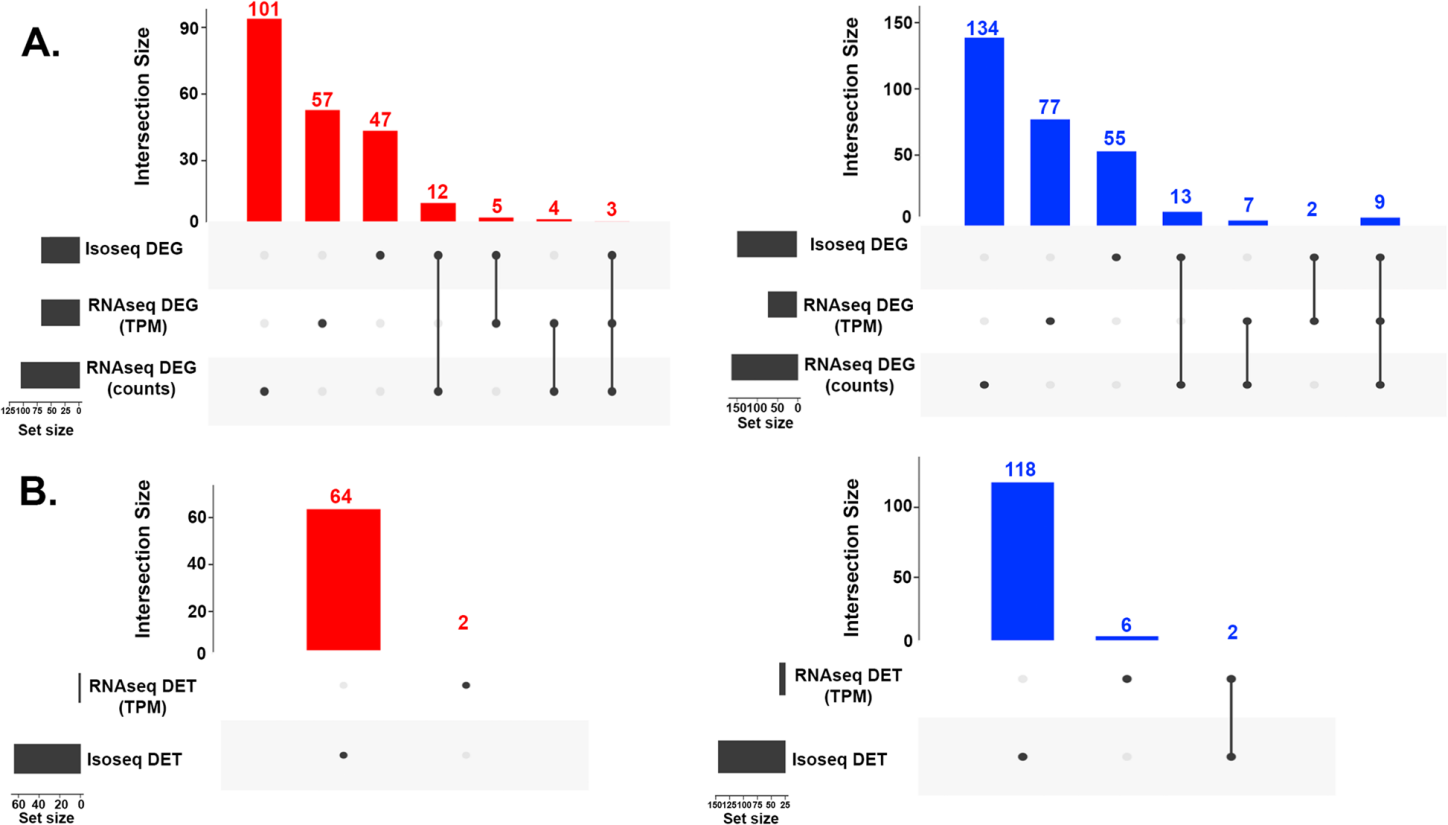
Overlap of differentially expressed genes and transcripts between different sequencing technologies and analysis methods. **A** The left panel shows up regulated genes in red and the right panel shows down regulated genes in blue. **B** The left panel shows up-regulated transcripts in red and down regulated transcripts in blue. DEG denotes differentially expressed genes; DET denotes differentially expressed transcripts. Notes: the datasets are ordered by set size. The denominator is Angus when interpreting up and down regulation e.g. red bars are up-regulation in Angus

The overlap of DEGs between sequencing technologies and analysis methods was examined to assess the potential biases in either approach (Fig. 2A). At the gene level, sequence counts for both Iso-Seq and RNA-seq identified the same 12 and 13 genes with increased and decreased frequency in Angus. There was less overlap in genes identified as differentially expressed between Iso-Seq and RNA-seq (TPM). Similarly, the overlap was also poor between RNA-seq (count) and RNA-seq (TPM).

The DEGs consistently identified by more than one analysis methods were considered as high confidence (Table 2). These high confidence DEGs are enriched for catalytic activity (GO:0,003,824), hydrolase activity (GO:0,016,787) and calcium ion binding (GO:0,005,509) (Fig. S7A). Seven of these high confidence DEGs are novel genes. Forty-four DEGs were identified by both Iso-Seq and RNA-seq, which included 6 novel genes.

**Table 2:** Overlap of DEGs and DETs identified by different methods. Only genes and transcripts that were found to be differentially expressed by more than one method are listed

Overlapped DEGs						
**Gene ID**	**Gene name**	**Protein name**	**Iso-Seq Q-value**	**RNA-Seq Q-value (Counts)**	**RNA-Seq Q-value (TPM)**	**Up- or down-regulated in Angus**
ENSBIXG00005021219	FAM13C	Family with sequence similarity 13 member C	4.13E-02	6.97E-03	4.57E-02	up
PB.41124	Novel gene	-	7.32E-03	4.37E-03	1.82E-03	up
ENSBIXG00005006815	SYT9	Synaptotagmin 9	5.91E-03	2.45E-02	5.13E-03	up
ENSBIXG00005007343	LOC113891523	5-hydroxytryptamine receptor 3E-like	NA	4.36E-04	1.30E-02	up
ENSBIXG00005021755	TNFSF18	TNF superfamily member 18	NA	1.40E-02	2.89E-08	up
PB.20446	Novel gene	-	NA	2.43E-02	1.64E-02	up
PB.20287	Novel gene	-	NA	1.25E-03	3.86E-02	up
ENSBIXG00005001387	REEP1	Receptor expression-enhancing protein	1.72E-02	NA	1.03E-03	up
ENSBIXG00005001405	GIMAP8	GTPase, IMAP family member 8	2.38E-02	NA	3.12E-03	up
ENSBIXG00005010189	PDE8B	Phosphodiesterase	3.96E-03	NA	5.13E-03	up
ENSBIXG00005008859	MGAM	Maltase-glucoamylase	2.75E-02	NA	1.18E-02	up
ENSBIXG00005002619	DIO1	Iodothyronine deiodinase	1.55E-02	NA	1.55E-02	up
ENSBIXG00005015707	PON1	Paraoxonase	1.31E-02	1.25E-03	NA	up
ENSBIXG00005028188	ALDH8A1	Aldehyde dehydrogenase 8 family member A1	2.73E-02	6.97E-03	NA	up
ENSBIXG00005003242	PCP4L1	Purkinje cell protein 4 like 1	4.13E-02	8.06E-03	NA	up
ENSBIXG00005031507	NT5E	Ecto-5'-nucleotidase	4.55E-02	1.05E-02	NA	up
ENSBIXG00005007237	LOC113890186	UDP-glucuronosyltransferase	4.24E-02	1.32E-02	NA	up
ENSBIXG00005018867	C1S	Complement C1s	2.73E-03	1.34E-02	NA	up
ENSBIXG00005000033	ERMP1	Endoplasmic reticulum metallopeptidase 1	3.88E-03	1.77E-02	NA	up
ENSBIXG00005008802	LOC113906361	Complement factor H	1.24E-02	2.22E-02	NA	up
ENSBIXG00005012404	-	-	4.13E-02	3.33E-02	NA	up
ENSBIXG00005000643	CDH17	Cadherin 17	7.32E-03	3.58E-02	NA	up
ENSBIXG00005018766	C1R	Complement subcomponent C1r	1.12E-02	3.78E-02	NA	up
ENSBIXG00005021852	CCND2	Cyclin D2	2.75E-02	3.95E-02	NA	up
PB.26206	Novel gene	-	1.29E-03	4.13E-02	4.68E-02	down
ENSBIXG00005001381	FRMD1	FERM domain containing 1	4.36E-04	4.58E-04	3.13E-10	down
ENSBIXG00005019601	LOC113900017	Inosine phosphorylase	7.50E-03	2.18E-03	1.55E-03	down
ENSBIXG00005006062	LRRK1	Non-specific serine/threonine protein kinase	9.00E-04	2.04E-02	4.15E-09	down
ENSBIXG00005004010	-	Ig-like domain-containing protein	1.25E-03	8.10E-04	3.71E-59	down
ENSBIXG00005012399	TGM2	Transglutaminase 2	1.59E-03	2.39E-02	1.25E-02	down
ENSBIXG00005013614	MINDY4	Ubiquitin carboxyl-terminal hydrolase MINDY	5.38E-03	2.04E-02	3.84E-04	down
ENSBIXG00005024440	KMO	Kynurenine 3-monooxygenase	6.81E-03	3.80E-02	2.47E-06	down
ENSBIXG00005027231	PTCHD4	Patched domain-containing protein 4-like	3.80E-02	3.96E-03	4.25E-07	down
ENSBIXG00005015655	-	RNA-directed DNA polymerase	NA	2.02E-02	4.86E-02	down
ENSBIXG00005007934	-	-	NA	1.25E-03	4.57E-02	down
PB.7890	Novel gene	-	NA	6.93E-03	5.33E-03	down
ENSBIXG00005027705	-	-	NA	4.36E-04	3.00E-07	down
ENSBIXG00005008786	ZNF804A	Zinc finger protein 804A	NA	2.07E-02	3.56E-04	down
ENSBIXG00005020160	LOC113882971	Serpin B4-like	NA	1.25E-03	1.03E-03	down
ENSBIXG00005015994	PI4KA	1-phosphatidylinositol 4-kinase	NA	1.74E-02	4.42E-02	down
ENSBIXG00005022489	RYR2	Ryanodine receptor 2	1.27E-02	NA	1.90E-10	down
ENSBIXG00005021285	-	-	2.86E-02	NA	3.25E-03	down
ENSBIXG00005003959	FAM169B	Family with sequence similarity 169 member B	9.00E-04	1.99E-02	NA	down
ENSBIXG00005010700	CPQ	Carboxypeptidase Q	1.25E-03	2.38E-02	NA	down
ENSBIXG00005022407	AQP3	Aquaglyceroporin-3	1.25E-03	4.13E-02	NA	down
ENSBIXG00005000868	SSC4D	Scavenger receptor cysteine rich family member with 4 domains	1.59E-03	7.32E-03	NA	down
ENSBIXG00005024637	TPO	Thyroid peroxidase	1.64E-03	1.70E-02	NA	down
ENSBIXG00005007913	MPPED1	Metallophosphoesterase domain containing 1	4.04E-03	1.40E-02	NA	down
ENSBIXG00005006455	LOC113882933	Serpin B3-like	3.16E-03	2.38E-02	NA	down
ENSBIXG00005021328	-	-	4.53E-03	5.41E-03	NA	down
ENSBIXG00005012422	IL1RL1	Interleukin 1 receptor like 1	7.39E-03	4.78E-02	NA	down
PB.14589	Novel gene	-	8.67E-03	8.43E-03	NA	down
PB.20615	Novel gene	-	1.92E-02	1.82E-02	NA	down
ENSBIXG00005007140	ENPP6	Choline-specific glycerophosphodiester phosphodiesterase	2.22E-02	4.93E-02	NA	down
ENSBIXG00005019829	SLC27A6	Solute carrier family 27 member 6	4.91E-02	2.02E-02	NA	down
**Overlapped DETs**						
**Transcript ID**	**Gene ID**	**Protein name**	**Iso-Seq Q-value**		**RNA-Seq Q-value (TPM)**	**Up or downregulated in Angus**
PB.35887.71	ENSBIXG00005004010	Ig-like domain-containing protein	2.94E-03		1.99E-04	down
PB.35887.157	ENSBIXG00005004010	Ig-like domain-containing protein	3.73E-02		1.45E-03	down

The overlap of differentially expressed transcripts is shown in Fig. 2B and the two DETs in common between analyses are listed at the bottom of Table 2. Only two novel transcripts, PB.35887.71 and PB.35887.157, which belong to the same gene ENSBIXG00005004010, were identified as significantly reduced in Angus in both Iso-Seq and RNA-seq. PB.35887.71 and PB.35887.157 were novel transcripts revealed by this Iso-Seq work and they are not currently available in the Ensembl annotation. The gene ENSBIXG00005004010 encodes an Ig-like domain-containing protein which was found to be reduced in Angus by all differential gene expression methods (Table 2).

### Discrepancy in calling differentially expressed genes

**Fig. 3:**
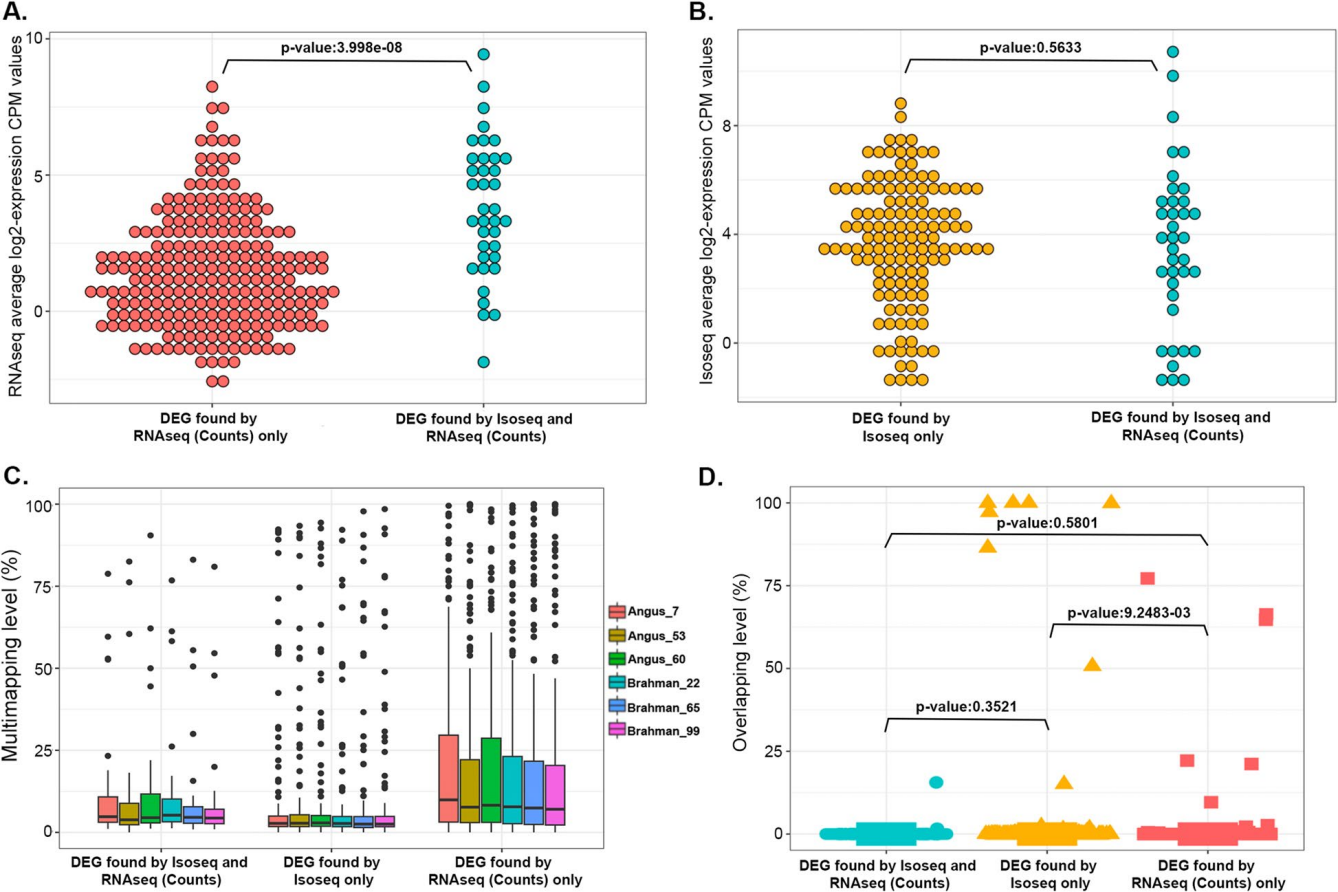
Discrepancy in DEGs called between RNA-seq and Iso-Seq. **A** Average expression of RNA-seq (counts) vs both Iso-Seq + RNA-seq (counts) (**B**) Average expression of Iso-Seq vs both Iso-Seq + RNA-seq (counts) (**C**) Multi-mapping level of all DEGs across three different categories grouped by analysis methods for each sample. For each DEG per sample, the level of multi-mapping was defined as: (the number of multi-mapped reads / total number of mapped reads) × 100%. **D** Overlapping of genomic coordinates of all DEGs across three different categories grouped by analysis methods. For each DEG, the percentage of overlap with other genes was calculated as: (length of overlapped positions with other genes / length of DEG) × 100%

Few genes were consistently identified as DEGs by more than one method, but those that were found by both RNA-seq (counts) and Iso-Seq had higher average expression (Table 2, Fig. 3A). The DEGs identified by RNA-seq (counts) only tend to have lower expression than those found by both Iso-Seq and RNA-seq (counts) (*p*-value < 0.05). However, the genes identified as DEGs by Iso-seq only did not show different average levels of expression than those found by both RNA-seq (counts) and Iso-Seq (Fig. 3B).

We assessed the extent of multi-mapped reads in DEGs by grouping them into “identified by RNA-seq only”, “identified by Iso-Seq only” and “both” for visualization. Multi-mapped reads are defined as RNA-seq reads in a DEG that not only mapped to the DEG but they also mapped elsewhere in the genome. Specifically, the “NH” tag in bam files with “NH:i:x > 1” was considered as a multi-mapped read. This analysis indicated that DEGs identified by RNA-seq had significantly (*p* < 0.05) higher numbers of multi-mapped reads than DEGs identified by Iso-Seq (Fig. 3C, Table S6). One source of multi-mapping of reads could be overlapping of more than one gene, such that a sequence can be potentially assigned to both genes. The short RNA-seq reads with multi-mapping were more difficult to confidently assign to a gene, and tend to be over counted hence reporting false DEGs. We also evaluated the performance of both technologies in dealing with overlapping between multiple genes. Assigning RNA-seq reads to genes that had overlapping genomic coordinates was more difficult than for Iso-Seq reads, as assignment was ambiguous for multiple genes. Therefore, significantly (*p* < 0.05) fewer DEGs called by RNA-seq had overlapping coordinates with other genes compared to those called by Iso-Seq (Fig. 3D).

### Iso-Seq identified differential transcript usage missed by RNA-seq

**Fig. 4:**
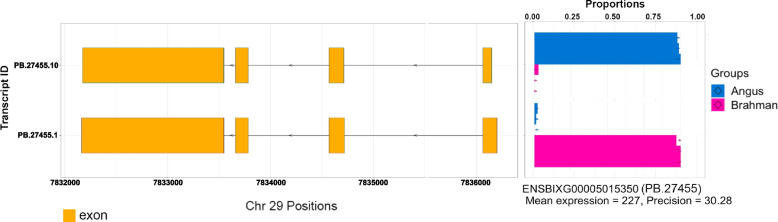
Significantly differential transcripts usage of gene *MRPL49* between Angus and Brahman. The structure of transcripts for gene *MRPL49*. The proportion of transcript usage in Angus (blue) and Brahman (pink)

Differential gene expression can involve overall higher or lower transcription of a gene or changes in the isoform usage for a gene. The possibility of differential expression at the transcript level was first examined using Iso-Seq data, which identified 721 genes that had significant differential transcript usage (Table S7). Some genes displayed breed specific transcript usage, e.g. *MRPL49* (ENSBIXG00005015350) that encodes the mitochondrial ribosomal L49 protein. Angus mainly expressed PB.27455.10 whereas Brahman used PB.27455.1. The main difference between the two transcripts is the 5’ UTR region, which is 52 bp longer in the Brahman specific transcript (Fig. 4). This is an example of genes with significant differential transcript usage that were identified from Iso-Seq data were missed by the RNA-seq analysis described below, which demonstrates the utility of Iso-Seq to fill in the gaps in knowledge of transcript usage.

Differential transcript expression was next examined using RNA-seq data, which identified 186 genes that had significant differential transcript usage between subspecies (Table S8), including 35 that were novel genes. For example, the gene ENSBIXG00005004010, that codes for Ig-like domain-containing protein, had the most statistically significant differential transcript usage. Six transcripts from this gene were found to be differentially expressed between the subspecies. Two (PB.35887.71, PB.35887.157 and PB.35887.148) were highly abundant (> 60% of transcripts from this gene) in Brahman fetal livers, while Angus samples had only negligible abundance of these two isoforms. Conversely, three other transcripts (PB.35887.74, PB.35887.169, PB.35887.89) represented > 10% of transcripts from this gene in Angus fetal livers while they had negligible abundance in Brahman samples.

There were 36 genes with differential transcript usage in common between those identified by RNA-seq and Iso-Seq (Table 3). There were 19 of these genes involved in the function of catalytic activity (GO:0,003,824, Q-value: 0.0331, Fig. S7B).

**Table 3:** Differential transcript usage genes identified by both RNA-seq and Iso-Seq.

**Gene ID**	**Gene name/symbol**	**Description**	**Iso-seq Q-value**	**RNA-seq Q-value**
ENSBIXG00005020385	MASP2	mannan binding lectin serine peptidase 2	8.78E-163	5.59E-08
ENSBIXG00005005332	NUSAP1	nucleolar and spindle associated protein 1	5.39E-112	6.30E-18
ENSBIXG00005022121	GRTP1	growth hormone regulated TBC protein 1	8.75E-35	2.52E-02
ENSBIXG00005009015	GAMT	guanidinoacetate N-methyltransferase	3.26E-25	1.86E-02
ENSBIXG00005007178	MCM10	minichromosome maintenance 10 replication initiation factor	8.05E-15	1.06E-07
ENSBIXG00005000548	PROC	protein C, inactivator of coagulation factors Va and VIIIa	2.34E-14	6.40E-23
ENSBIXG00005005056	IVD	isovaleryl-CoA dehydrogenase	1.10E-13	2.63E-02
ENSBIXG00005024440	KMO	kynurenine 3-monooxygenase	5.18E-12	5.87E-04
ENSBIXG00005023567	PIGW	phosphatidylinositol glycan anchor biosynthesis class W	1.29E-10	3.80E-02
ENSBIXG00005008286	AGTR1	angiotensin II receptor type 1	1.60E-10	3.43E-08
ENSBIXG00005016748	TTC3	tetratricopeptide repeat domain 3	1.03E-09	2.13E-04
ENSBIXG00005006900	FECH	ferrochelatase	4.13E-09	2.82E-04
ENSBIXG00005011852	PTGR1	prostaglandin reductase 1	9.55E-09	4.68E-41
ENSBIXG00005004648	PSMC2	proteasome 26S subunit, ATPase 2	5.31E-08	2.13E-02
ENSBIXG00005031552	CLK1	CDC like kinase 1	8.38E-07	1.85E-05
ENSBIXG00005030508	PAM	peptidylglycine alpha-amidating monooxygenase	1.13E-06	2.75E-02
ENSBIXG00005017614	FAM241B	Family with sequence similarity 241 member B	2.08E-05	9.85E-04
ENSBIXG00005010669	ALAD	aminolevulinate dehydratase	4.47E-05	6.79E-06
ENSBIXG00005007376	C5H12orf75	chromosome 5 C12orf75 homolog	4.86E-05	4.40E-09
ENSBIXG00005003661	LOC113876353	very-long-chain 3-oxoacyl-CoA reductase-B-like	7.61E-04	1.83E-18
ENSBIXG00005020956	TPM1	tropomyosin 1	9.18E-04	6.40E-04
ENSBIXG00005003152	LIPC	lipase C, hepatic type	9.78E-04	5.33E-05
ENSBIXG00005030889	GHITM	growth hormone inducible transmembrane protein	0.00110707	3.05E-02
ENSBIXG00005020914	SEC31A	SEC31 homolog A, COPII coat complex component	0.00116596	0.016632608
ENSBIXG00005018018	CALM1	calmodulin 1	0.0012903	0.009381684
ENSBIXG00005030960	CAST	calpastatin	0.0039888	0.014115567
ENSBIXG00005009706	AVPI1	arginine vasopressin induced 1	0.00401675	0.021481672
ENSBIXG00005024769	ERG28	RNA-directed DNA polymerase	0.00592012	7.12E-06
ENSBIXG00005022063	AK4	adenylate kinase 4	0.01056799	2.85E-32
PB.36968			0.01114297	5.31E-06
ENSBIXG00005031540	ARG1	arginase 1	0.0125591	3.58E-07
ENSBIXG00005010835	LOC113889558	vascular cell adhesion protein 1-like	0.02095845	1.49E-09
ENSBIXG00005029121	ACP5	acid phosphatase 5, tartrate resistant	0.02214711	0.025738332
ENSBIXG00005000161	EIF4B	eukaryotic translation initiation factor 4B	0.03090533	0.02907026
ENSBIXG00005008273	CMAS	cytidine monophosphate N-acetylneuraminic acid synthetase	0.03220453	0.010395561
ENSBIXG00005019414	RCL1	RNA terminal phosphate cyclase like 1	0.04492615	0.011265087

### Iso-Seq resolves immunoglobulin-like domain-containing transcripts

**Fig. 5:**
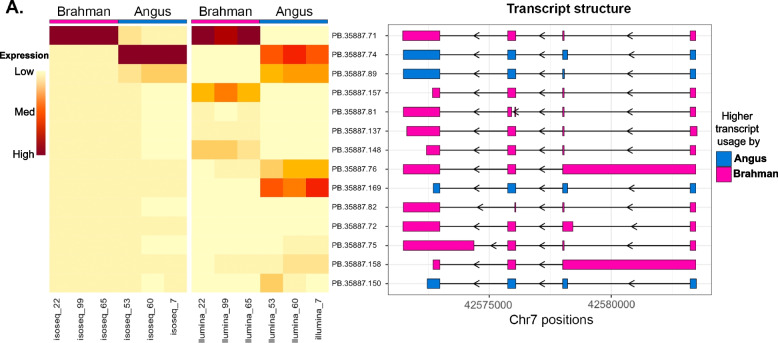
Expression level of common transcripts between technologies for gene encoded for Ig-like domain-containing protein and their structures. On left, the expression level of common transcripts found in both Iso-Seq and RNA-seq for gene (PB.35887, ENSBIXG00005004010) that encodes for Ig-like domain-containing protein. On right, for the transcript structure, the transcripts were colored by higher transcript usage in either Angus (blue) or Brahman (pink) according to Iso-Seq data

The immunoglobulin-like domain-containing gene (ENSBIXG00005004010) was consistently detected as having differential abundance in Angus or Brahman fetal liver at both gene and transcript levels. The expression and structure of all transcripts for this gene were studied in detail. There were 75 unique transcripts detected for this gene by at least one sequence read in at least one dataset, of which 21 were expressed at a high enough level to be reliably identified by Iso-Seq and 31 identified by RNA-seq. There were 37 predicted transcripts that were in too low abundance for confident assignment and 14 transcripts could be confidently detected by both sequencing technologies. The abundance of these 14 shared transcripts was compared in Angus and Brahman samples (Fig. 5). The overall differential expression at the gene level was driven by subspecies-specific variation in abundance of transcript PB.35887.71, which was predominantly expressed in Brahman livers. The result of both Iso-Seq and RNA-seq were generally in agreement with respect to expression of this transcript. However, for some of the transcripts detected for this gene, differences between Iso-Seq and RNA-seq analyses were observed that were, in part, because RNA-seq could not disentangle transcripts with similar exons and introns. For example, both PB.35887.76 and PB.35887.169 transcripts produced identical protein but PB.35887.169 has a longer 5’UTR. In general, another potential reason for the difference could be the depth of our Iso-Seq was insufficient at quantifying lowly expressed transcripts.

## Discussion

This study used deep sequencing by Iso-Seq and standard depth of RNA-seq to compare the DEGs and DETs predicted by each and to examine the differences in gene expression results using full length reads. Iso-Seq depth was > 9 million CCS reads per sample, enabling higher resolution quantification of gene expression than most previous studies with full-length cDNA sequencing. For comparisons, there were two other cattle Iso-Seq datasets, one with ~ 3.3 million FLNC reads from seven pooled tissues [6] and another with only 276,295 FLNC reads from six tissues [39]. The main purpose of these Iso-Seq datasets was genome annotation. Our study used more than 7.6 million FLNC reads per sample. Standard sequencing depth of RNA-seq (30–50 million paired reads) for transcriptome characterization was available for the same samples, including three biological replicate samples of fetal liver from each of two cattle subspecies. This experimental design enabled an estimate of concordance between quantification of gene expression, transcript expression and transcript usage by long- and short-read technology.

The comparison between short and long read characterization of differential gene expression and transcript abundance in the present study indicated that the technology used has a major impact on results. Specifically, there was a larger influence of technology than of biology between Angus and Brahman fetal liver transcriptome characterization, with higher correlations across subspecies within technology than within subspecies but across technology. This was the case at both gene- and transcript-level expression and is consistent with a previous study in bears [13]. Nevertheless, significant positive correlations between Iso-Seq and RNA-seq expression levels have been reported in mouse and human cortex [8], although these other studies did not quantify gene expression directly with Iso-Seq data, presumably due to the much lower Iso-Seq sequence depth than we had available. In the present study, we found differences in DEGs, DETs and DTUs between Iso-Seq and RNA-seq.

The DEGs found between Angus and Brahman fetal livers were not completely consistent between Iso-Seq and RNA-seq based methods. One potential source for the discrepancy in identifying DEGs is the amplification step of the Iso-Seq library preparation, in which the length of transcripts being amplified span a range from hundreds to thousands of bases. This wide range can lead to amplification biases based on length. In addition, the Iso-Seq library is size-selected using binding beads providing an additional source of potential bias. Finally, longer-range amplification in Iso-Seq template preparation might lead to “drop out” of transcripts at low abundance in the source RNA, i.e. low expression-level genes might not have sufficient depth to accurately evaluate mean expression level among samples. This potential bias would be expected to impact the overlap of DEG definition between technologies to favour genes at relatively high expression level, and indeed DEGs that had high average expression were found consistently by both RNA-seq and Iso-Seq, but DEGs with low levels of expression tended to be found only by RNA-seq. The Iso-Seq rarefaction curve had reached plateau at the gene-level in the samples of our study, but despite this observation it is likely that greater depth is required to quantify transcripts with a low level of expression and support confident identification of DEGs.

RNA-seq based methods have intrinsic limitations and biases, including the requirement to map the short reads to a reference genome or transcriptome. The alignment process usually leads to some reads being aligned with more than one genomic location [40] and produced biased analysis for genes with copy number higher than one or where two distinct genes have overlapping exons. This was a particular problem for the RNA-seq dataset used here as it included two subspecies that have diverged substantially with many known differences in duplicated genes and repeats [6, 41]. More DEGs found by RNA-seq alone had multi-mapped reads than those identified using both methods, suggesting that interpretations based on RNA-seq should be treated with caution [21] as these could be an artifact resulting from duplicated sequences. We suspect that studies comparing groups of highly diverged samples using RNA-seq will also have erroneously called some genes as DEGs, which are an artifact of sequence duplication. False negative identification of DEGs can also occur in RNA-seq analysis for genes that overlap other genes, for example due to the default setting of Feature Count which is set to ignore reads assigned to more than one feature e.g. gene as a feature.

Only one gene displayed evidence of DEG, DET and DTU. The gene encodes an Ig-like domain containing protein and had 75 unique predicted transcripts associated with it although only a fraction of those isoforms had sufficient counts to be considered for statistical analysis. The expression pattern of this gene was distinct between the two cattle breeds with higher gene-level expression in Brahman principally represented by two transcripts. Interestingly, the transcripts preferentially used by Angus were different from Brahman, hence this gene also showed evidence of DTU. The Ig-like domain has been previously described [42] as a common protein domain that potentially has a role in the immune system.

There were 55 DEGs that we designated as high confidence based on being identified by more than one analysis methods. These included Cyclin D2 (*CCND2*), which is known to play a role in cell growth and proliferation. In a genome wide association study (GWAS), *CCND2* has been associated with average daily weight gain in Hereford cattle [43] and five SNPs flanking *CCND2* have been associated with body weight in Siberian cattle [44]. The higher expression of *CCND2* in Angus may therefore be associated with higher liver weight observed during fetal development compared to Brahman (unpublished data).

Two high confidence DEGs, phosphodiesterase 8B (*PDE8B*) and zinc finger protein 804A (*ZNF804A*) have previously been identified as expression master regulators that are associated with meat quality in beef cattle [45]. These master regulators influence the gene expression of several other genes, including other DEGs found in this work. The differential expression may also be associated with the observed superior meat quality of Angus compared to Brahman cattle. In humans, *PDE8B* is highly expressed in the thyroid gland [46] and may play a role in signalling of physiological processes. It is also interesting to note that two other high confidence DEGs, iodothyronine deiodinase 1 (*DIO1*) and thyroid peroxidase (*TPO*), are also associated with thyroid function. Thyroid hormone is a major driver of fetal growth, sexual differentiation and gonadal development in animals [47], therefore these high confidence DEGs may suggest genetic regulatory networks influencing fetal development.

Few studies have explored the transcriptome of cattle using long read sequencing. A previous Iso-Seq analysis in taurine cattle, that examined six tissues (cerebrum, rumen, liver, spleen, renal cortical and longissimus muscle) with a small number of CCS reads, identified new gene models and transcripts, and alternative polyadenylation sites [39]. We have also been able to improve genome annotation using Iso-Seq data for seven cattle tissues (brain, heart, kidney, liver, lung, muscle and placenta) [6]. In the present work, using Iso-Seq data, we detected 237 genes in the liver tissue that were not in the reference transcriptome. This demonstrates the benefits of using Iso-Seq to detect novel genes and transcripts, as genome annotation has typically used RNA-seq data to form gene models and so was unable to accurately define alternative spliced transcripts.

This study demonstrates the utility of Iso-Seq to uncover hidden mammalian transcriptional complexity, not seen by RNA-seq alone. The lower output and higher relative cost of Iso-Seq has limited its use beyond discovery of transcript isoforms and genome annotation, but we suggest that the use of RNA-seq alone may introduce bias in the evaluation of transcriptome complexity and differential gene expression. Improvements in the throughput of Iso-Seq or other long read sequencing technologies will lead to increased availability for the characterization of RNA populations and routine quantification of gene expression in the future.

## Supplementary Information

**Additional file 1: Table S1.** Significant DEGs (*p*-value <0.05) identified from Iso-Seq dataset using limma linear model. **Table S2.** DEGs identified by RNA-seq technology using RfeatureCounts and limma linear model with *p*-value <0.05. **Table S3.** DEGs identified by RNA-seq technology using sleuth with *p*-value <0.05. **Table S4.** DETs identified by Iso-Seq technology using limma Linear model with *p*-value <0.05. **Table S5.** DETs identified by RNA-Seq technology using sleuth with *p*-value <0.05. **Table S6.** The *p*-values from Wilcoxon test of the significance of different multi-mapping levels between three groups (DEGs identified by RNA-seq, DEGs identified by Iso-Seq, DEGs identified by both). **Table S7.** The significant DTUs (*p* < 0.05) identified using Iso-Seq technology.** Table S8.** The significant DTUs (*p* < 0.05) identified using Iso-Seq technology. **Figure S1.** An overview of the analysis pipeline used to generate full-length transcript annotations in Brahman and Angus liver samples. In expression correlation, pair-wise correlation plots were made between the outputs for the same colors (green and grey). Similarly, the comparisons were made for the same colors (purple, yellow, pink and orange) in sequencing technology comparison. **Figure S2.** Rarefaction curve of Iso-Seq data for gene and transcript level. **Figure S3.** Number of isoforms per gene identified by Iso-Seq. (A) Number of isoforms per gene separated by known and novel genes. (B) The distribution of number of isoforms per gene. **Figure S4.** Transcript distribution across structural categories and the distribution of transcript length identified by SQANTI3. Full Splice Match (FSM), Incomplete Splice Match (ISM), Novel in Catalog (NIC), and Novel Not in Catalog (NNC). **Figure S5.** The percentage of overlapping genes in DEGs. The percentage of overlapping DEGs identified by RNA-seq only, the percentage of overlapping DEGs identified by Iso-Seq only and the percentage of overlapping DEGs identified by both are presented. **Figure S6.** Proportion of differential transcript usage for gene encoding Ig-like domain-containing protein. This transcript usage result was done with RNA-seq data. **Figure S7.** The GO pathway analysis results on high confidence DEGs (A) and DTUs (B).

## Data Availability

The PacBio dataset of this work is available in the SRA under BioProject PRJNA626458. The code to perform various gene and transcript expression studies is available at https://github.com/DaviesCentreInformatics/Angus_Brahman_Iso-Seq.
