# Supplementary material for: Long read isoform sequencing reveals hidden transcriptional complexity between cattle subspecies
Source: BMC Genomics. 2023 Mar 13;24:108. doi: 10.1186/s12864-023-09212-9 (PMC10012480; doi:10.1186/s12864-023-09212-9)

SUPPLEMENTARY DATA

Tables

Table S1. Significant DEGs (p-value <0.05) identified from Iso-Seq dataset using limma linear model.

Gene ID	logFC	log2 Average Expression	P-value	Q-value	Up or downregulated in Angus
ENSBIXG00005001381	-5.9267846	3.86453313	4.37E-08	4.58E-04	down
ENSBIXG00005029834	-4.5759322	3.96580108	7.17E-08	4.58E-04	down
ENSBIXG00005004010	-3.3576182	9.83096559	2.47E-07	8.10E-04	down
ENSBIXG00005001579	-5.5203288	4.3280436	2.54E-07	8.10E-04	down
ENSBIXG00005019496	-9.6943347	1.21307963	5.27E-07	0.00134458	down
ENSBIXG00005023663	-3.0666924	3.90690428	1.10E-06	0.00218048	down
ENSBIXG00005019601	-2.6185932	7.07906573	1.20E-06	0.00218048	down
ENSBIXG00005007230	-3.7874606	2.94813834	1.64E-06	0.00260938	down
ENSBIXG00005002897	-3.0186501	3.21064917	3.24E-06	0.0038106	down
ENSBIXG00005019252	-7.1554232	-0.0400954	3.28E-06	0.0038106	down
ENSBIXG00005027231	-3.4176015	4.21161116	4.28E-06	0.00396229	down
ENSBIXG00005012082	-3.0397583	2.9726776	4.80E-06	0.00408243	down
ENSBIXG00005019123	-2.4793712	3.27471986	5.41E-06	0.00431966	down
ENSBIXG00005020118	-2.5455645	3.10050507	6.32E-06	0.00474794	down
ENSBIXG00005021328	-6.2611718	-0.4697864	7.63E-06	0.00540817	down
ENSBIXG00005015511	-1.6651846	5.86731921	9.26E-06	0.0059096	down
ENSBIXG00005025666	-1.7304619	7.34263175	1.05E-05	0.00637029	down
ENSBIXG00005005261	-2.1728765	4.56227708	1.33E-05	0.00731884	down
ENSBIXG00005000868	-3.9700427	3.61820982	1.54E-05	0.00731884	down

ENSBIXG00005013111	-3.276213	3.91733101	1.72E-05	0.00786056	down
PB.14589 (chr18: 54423463- 54430781)	-5.6654011	-0.6870252	1.92E-05	0.00843178	down
ENSBIXG00005009520	-4.6000124	4.11566149	2.07E-05	0.00879468	down
ENSBIXG00005021776	-2.3317184	3.33581701	2.35E-05	0.00967848	down
ENSBIXG00005014257	-6.3066757	-0.5006625	3.05E-05	0.01179738	down
ENSBIXG00005022489	-5.0838023	1.27930924	3.37E-05	0.01243168	down
ENSBIXG00005007913	-7.8456011	1.3276722	4.15E-05	0.01395019	down
ENSBIXG00005027457	-5.1296609	-1.0189032	4.51E-05	0.01463014	down
ENSBIXG00005016416	-6.9549156	-0.2212861	5.22E-05	0.01552759	down
ENSBIXG00005018837	-3.9122581	3.29255189	5.31E-05	0.01552759	down
ENSBIXG00005024637	-5.9696076	-0.5014907	6.27E-05	0.01703024	down
PB.20615 (chr22: 5534177-5542770)	-4.5712326	2.43414924	7.28E-05	0.01819167	down
ENSBIXG00005009662	-2.3028637	3.50768654	7.67E-05	0.01819167	down
ENSBIXG00005030516	-7.6657466	1.03731616	7.82E-05	0.01819167	down
ENSBIXG00005003959	-4.4214392	2.50261686	8.87E-05	0.01986143	down
ENSBIXG00005019829	-2.1841719	4.92483022	9.26E-05	0.02021349	down
ENSBIXG00005018073	-1.8557381	4.10148466	9.34E-05	0.02021349	down
ENSBIXG00005006062	-1.9733244	2.72969554	9.74E-05	0.02035144	down
ENSBIXG00005013614	-1.4325129	4.55700294	9.80E-05	0.02035144	down
ENSBIXG00005006169	-1.3823489	5.74960195	1.14E-04	0.02272898	down
ENSBIXG00005021652	-2.3033651	2.3452274	1.25E-04	0.02382965	down
ENSBIXG00005010700	-3.0165722	1.8567057	1.25E-04	0.02382965	down
ENSBIXG00005006455	-4.790989	-1.2253749	1.29E-04	0.02382965	down

ENSBIXG00005012399	-1.6753229	6.27364061	1.31E-04	0.02389018	down
ENSBIXG00005018011	-1.3186202	8.81545184	1.59E-04	0.02741037	down
ENSBIXG00005003117	-2.7843736	1.77040902	1.61E-04	0.02741037	down
ENSBIXG00005029593	-3.162377	1.91547855	1.70E-04	0.0275396	down
ENSBIXG00005021747	-1.7770168	3.61903516	1.79E-04	0.0275396	down
ENSBIXG00005029109	-1.73261	3.61393367	1.79E-04	0.0275396	down
ENSBIXG00005023700	-1.9852068	5.1030397	1.82E-04	0.0275396	down
ENSBIXG00005006342	-3.2335749	2.12689338	1.85E-04	0.0275396	down
ENSBIXG00005002987	-1.3717142	5.0067639	1.88E-04	0.0275396	down
ENSBIXG00005021285	-2.0258533	4.03545974	1.90E-04	0.02754774	down
ENSBIXG00005001982	-1.3392973	5.72914216	1.94E-04	0.02786172	down
ENSBIXG00005015746	-1.3631762	4.4628686	2.11E-04	0.02869422	down
ENSBIXG00005020961	-1.5447254	4.78629139	2.17E-04	0.02869422	down
ENSBIXG00005018941	-1.4884778	5.67056038	2.17E-04	0.02869422	down
ENSBIXG00005004545	-1.6719263	3.41211961	2.22E-04	0.02869422	down
ENSBIXG00005011368	-1.6420108	4.06134598	2.24E-04	0.02869422	down
ENSBIXG00005002416	-1.2931766	4.67068459	2.24E-04	0.02869422	down
ENSBIXG00005030552	-2.9781694	2.63934664	2.38E-04	0.03005644	down
ENSBIXG00005009142	-1.5669812	3.46257884	2.51E-04	0.03068281	down
ENSBIXG00005022036	-1.2551142	5.77665372	2.52E-04	0.03068281	down
ENSBIXG00005010712	-1.8986233	5.24071109	2.72E-04	0.0325697	down
ENSBIXG00005012054	-1.3916635	5.61301326	2.82E-04	0.0325697	down
ENSBIXG00005027312	-3.1507156	2.8133475	2.83E-04	0.0325697	down
ENSBIXG00005002251	-4.8107151	-0.1230169	2.96E-04	0.03376331	down
ENSBIXG00005019987	-4.2974449	-1.4498207	3.09E-04	0.03493441	down

ENSBIXG00005009372	-4.9944334	0.13990769	3.34E-04	0.03670333	down
ENSBIXG00005027919	-5.3277822	1.93251317	3.53E-04	0.03804392	down
ENSBIXG00005004090	-3.3020227	0.8390121	3.55E-04	0.03804392	down
ENSBIXG00005024440	-1.5635393	3.78003444	3.58E-04	0.03804392	down
ENSBIXG00005002007	-1.1197498	6.01974114	3.62E-04	0.03814451	down
ENSBIXG00005000323	-1.0609371	7.27920195	4.07E-04	0.04134544	down
ENSBIXG00005016718	-4.2650488	-1.4818093	4.17E-04	0.04134544	down
ENSBIXG00005014624	-1.3176247	5.76616025	4.24E-04	0.04134544	down
ENSBIXG00005023256	-2.0768042	2.03938881	4.25E-04	0.04134544	down
ENSBIXG00005006548	-1.5404249	3.68345507	4.27E-04	0.04134544	down
PB.26206 (chr27: 38634482-38736901)	-4.2375256	-1.4875804	4.34E-04	0.04134544	down
ENSBIXG00005022407	-4.8347527	-0.3764812	4.40E-04	0.04134544	down
ENSBIXG00005007656	-1.5354285	4.16267766	4.48E-04	0.04134544	down
ENSBIXG00005028296	-4.2219114	-1.3813995	4.50E-04	0.04134544	down
PB.13415 (chr18: 4870151- 4869660)	-1.2575551	6.31725785	4.59E-04	0.04134544	down
ENSBIXG00005008437	-4.1962283	-1.3905527	4.63E-04	0.04134544	down
ENSBIXG00005031028	-1.3892053	4.69111391	4.70E-04	0.04154244	down
ENSBIXG00005010243	-1.1073029	8.22174871	4.72E-04	0.04154244	down
ENSBIXG00005031539	-1.5434641	3.31069525	4.79E-04	0.04159429	down
ENSBIXG00005007092	-2.2026395	5.8448256	4.95E-04	0.04237084	down
ENSBIXG00005029852	-1.4031328	4.27064755	5.01E-04	0.04264123	down
ENSBIXG00005015098	-1.1846917	7.0976492	5.21E-04	0.04402328	down
ENSBIXG00005017232	-1.52605	4.09488156	5.26E-04	0.04418471	down

ENSBIXG00005000553	-2.3034869	2.5273238	5.33E-04	0.04438513	down
ENSBIXG00005015011	-1.4061808	3.49233669	5.39E-04	0.04439308	down
ENSBIXG00005017154	-1.196615	5.3804907	5.62E-04	0.04549479	down
ENSBIXG00005023232	-2.1801296	5.49369502	5.82E-04	0.04651907	down
ENSBIXG00005011859	-1.5069302	4.71604484	5.83E-04	0.04651907	down
ENSBIXG00005028467	-1.7357757	5.67682392	5.99E-04	0.04749418	down
ENSBIXG00005012422	-1.6022253	4.66924114	6.07E-04	0.04780028	down
ENSBIXG00005004662	-1.4551285	4.38123072	6.35E-04	0.04879647	down
ENSBIXG00005014737	-1.3960519	5.99753054	6.41E-04	0.04897955	down
ENSBIXG00005007140	-2.0020517	3.15702735	6.49E-04	0.04928241	down
ENSBIXG00005002234	-1.4247657	3.57711143	6.55E-04	0.04944914	down

Table S2. DEGs identified by Iso-RNA technology using RfeatureCounts and limma linear model with p-value <0.05.

Gene ID	logFC	log2 Average Expression	P-value	Q-value	Up or downregulated in Angus
ENSBIXG00005007343	6.09888197	0.49282539	7.99E-08	4.36E-04	up
PB.38967(chr9: 10706295-10730287)	3.61446265	0.22844238	3.43E-07	9.00E-04	up
PB.21751(chr22: 54780705-54788972)	4.18662887	-1.3038297	3.96E-07	9.00E-04	up
ENSBIXG00005015707	1.94035532	3.417218	1.11E-06	0.00125356	up
PB.20287(chr21: 65547820-65557338)	3.74535704	0.34916894	1.17E-06	0.00125356	up
PB.19309(chr21: 610908-614172)	4.20138588	-2.4144941	1.51E-06	0.0013063	up

ENSBIXG00005021014	4.71565993	-1.9769579	5.86E-06	0.00315958	up
ENSBIXG00005006053	1.51250653	2.57549657	6.12E-06	0.00315958	up
PB.41124(chrX: 35004902-35007294)	5.40842353	0.78363373	1.01E-05	0.00437238	up
ENSBIXG00005011405	1.95606668	0.43139135	1.06E-05	0.00444051	up
ENSBIXG00005019156	3.42968889	-0.7681947	1.09E-05	0.00447921	up
ENSBIXG00005024909	2.64216855	1.6066366	1.21E-05	0.00453185	up
ENSBIXG00005020560	2.17114995	-0.5674355	1.27E-05	0.00453185	up
ENSBIXG00005011413	1.13547648	3.09083093	1.70E-05	0.00538345	up
ENSBIXG00005004000	1.42784363	3.55004878	2.00E-05	0.00604122	up
PB.13804(chr18: 14956282-14964029)	3.2937234	-0.748553	2.25E-05	0.00646551	up
ENSBIXG00005009833	1.68974603	5.40655906	2.51E-05	0.00681407	up
PB.18705(chr20: 11553845-11643313)	1.66733143	3.94347633	2.56E-05	0.00681407	up
ENSBIXG00005022689	1.07894365	4.84464348	2.64E-05	0.00681407	up
ENSBIXG00005000540	4.65941908	0.7458867	3.26E-05	0.00697253	up
ENSBIXG00005021219	1.10678857	5.15332837	3.26E-05	0.00697253	up
PB.25816(chr27: 14825078-14834737)	1.368788	2.2104039	3.36E-05	0.00697253	up
ENSBIXG00005017907	1.62960541	2.77061635	3.37E-05	0.00697253	up
ENSBIXG00005024295	2.51702723	-0.8425014	3.40E-05	0.00697253	up
ENSBIXG00005028188	1.6234143	5.54021842	3.40E-05	0.00697253	up
ENSBIXG00005003242	1.20908875	3.14933344	4.28E-05	0.00805666	up
ENSBIXG00005005851	3.25507872	-1.5632823	4.43E-05	0.00815002	up
ENSBIXG00005004154	1.22662291	2.11870969	4.59E-05	0.00822501	up

PB.41363(chrX: 52900940-52928589)	1.62893701	1.12482501	5.02E-05	0.00866566	up
ENSBIXG00005004338	1.0945648	4.57112123	5.64E-05	0.0093288	up
PB.7629(chr13: 37848046-37855644)	1.15569264	3.20331195	6.32E-05	0.01035676	up
ENSBIXG00005031507	1.20170744	3.93641806	6.52E-05	0.01052276	up
ENSBIXG00005017915	2.57990833	3.1835711	7.02E-05	0.01066208	up
PB.3181(chr10: 60342426-60555219)	2.53906929	1.91847551	7.75E-05	0.01154776	up
ENSBIXG00005018143	1.00318921	4.14075903	8.96E-05	0.01298596	up
ENSBIXG00005027431	1.19757478	6.94358287	9.25E-05	0.01321812	up
ENSBIXG00005007237	1.15162164	2.9290211	9.28E-05	0.01321812	up
ENSBIXG00005018867	2.87031646	5.6050693	9.55E-05	0.01337091	up
ENSBIXG00005021755	1.04905838	8.33863944	1.02E-04	0.01402806	up
ENSBIXG00005021694	1.331817	3.61763852	1.14E-04	0.01491371	up
ENSBIXG00005015702	1.13236442	0.9900013	1.36E-04	0.01703643	up
ENSBIXG00005021166	1.72309653	0.79573346	1.46E-04	0.01742763	up
ENSBIXG00005030272	1.23511964	2.42258574	1.49E-04	0.01751958	up
ENSBIXG00005000033	1.00094575	7.60119585	1.53E-04	0.01773086	up
ENSBIXG00005031161	1.20462683	2.1468433	1.59E-04	0.01806608	up
ENSBIXG00005015183	1.32284538	3.72505161	1.73E-04	0.01877835	up
ENSBIXG00005015901	5.28043573	1.14014432	1.74E-04	0.01877835	up
ENSBIXG00005029698	1.04102356	1.46577304	1.76E-04	0.01890072	up
ENSBIXG00005017802	2.24556321	2.01855397	1.78E-04	0.01895632	up
ENSBIXG00005011092	1.96864584	1.55605939	1.95E-04	0.0197843	up
PB.19222(chr20:	1.12158227	3.67720913	2.06E-04	0.02015108	up

62192943-62226623)					
ENSBIXG00005010604	1.69880985	-1.4505985	2.15E-04	0.0205874	up
ENSBIXG00005008039	1.46775512	-1.1485052	2.43E-04	0.02179613	up
ENSBIXG00005017641	1.13293063	2.16853674	2.49E-04	0.02203663	up
ENSBIXG00005006106	1.01686613	1.40273958	2.52E-04	0.02203663	up
ENSBIXG00005021359	2.31311652	3.2547838	2.53E-04	0.02203663	up
ENSBIXG00005020819	1.45627023	-0.2807899	2.56E-04	0.02205766	up
ENSBIXG00005009315	1.01739555	2.22357441	2.60E-04	0.02205766	up
PB.12185(chr17: 3827257-3835044)	1.08499106	1.1921553	2.71E-04	0.02218873	up
ENSBIXG00005008802	1.9515255	9.43194784	2.72E-04	0.02218873	up
PB.30149(chr4: 171496-196721)	1.91048364	2.05182349	2.88E-04	0.02266956	up
PB.22934(chr24: 4623330-4631529)	1.74783341	-1.2198125	2.93E-04	0.02291172	up
PB.4832(chr11: 60046745-60059687)	1.47769752	-0.3377097	3.01E-04	0.02313399	up
ENSBIXG00005031306	1.20955779	1.37430547	3.25E-04	0.02400497	up
ENSBIXG00005027266	1.46119341	2.32291779	3.28E-04	0.0240637	up
ENSBIXG00005029039	2.32622613	0.69136587	3.29E-04	0.0240637	up
PB.20446(chr22: 161013-169322)	1.63461905	2.14031944	3.45E-04	0.02432878	up
ENSBIXG00005006815	1.83995819	5.12552267	3.54E-04	0.02448355	up
ENSBIXG00005016002	1.58351157	1.51272481	3.61E-04	0.02465218	up
ENSBIXG00005024973	1.46935385	-1.5658462	3.64E-04	0.02473457	up
ENSBIXG00005025795	1.4716184	0.59551177	3.84E-04	0.02557904	up
ENSBIXG00005000621	1.16063417	0.37623887	3.96E-04	0.02582515	up

ENSBIXG00005024522	2.14858355	-0.3258649	5.08E-04	0.02908572	up
ENSBIXG00005003622	1.10254088	3.69886838	5.09E-04	0.02908572	up
ENSBIXG00005026237	1.69180316	-0.8850603	5.58E-04	0.03027457	up
ENSBIXG00005027901	1.15686185	0.67447875	6.08E-04	0.03167894	up
ENSBIXG00005026949	1.22195961	3.28018819	6.09E-04	0.03167894	up
ENSBIXG00005021239	1.97013591	0.69963484	6.15E-04	0.03183008	up
ENSBIXG00005012404	2.12093178	2.18102969	6.72E-04	0.03325365	up
ENSBIXG00005022653	1.32169687	3.63847086	6.79E-04	0.03331622	up
ENSBIXG00005001680	1.8851338	2.89756095	7.20E-04	0.0341852	up
ENSBIXG00005000643	2.02702113	3.85832341	7.67E-04	0.03582038	up
PB.27666(chr29: 18031419-18035208)	1.43776488	-0.0626636	7.93E-04	0.03638271	up
PB.41369(chrX: 53048972-53071938)	1.13623957	0.6439153	8.08E-04	0.03648729	up
ENSBIXG00005029082	1.40516564	-0.5560183	8.17E-04	0.03648729	up
ENSBIXG00005029704	1.22796243	6.41386796	8.18E-04	0.03648729	up
ENSBIXG00005022644	2.41661519	-0.3149959	8.32E-04	0.03657196	up
ENSBIXG00005012692	1.85226768	3.63889875	8.42E-04	0.03673627	up
ENSBIXG00005000647	1.22900716	2.31754896	8.50E-04	0.03673627	up
ENSBIXG00005009443	2.01140194	0.137852	8.54E-04	0.03673916	up
PB.7778(chr13: 47091654-47120427)	1.23554532	0.76903919	8.67E-04	0.03713936	up
ENSBIXG00005028454	1.537022	0.38167956	8.80E-04	0.0373732	up
ENSBIXG00005018766	1.34190178	5.51629475	9.00E-04	0.03780473	up
ENSBIXG00005028017	1.28268794	1.36796139	9.36E-04	0.03797501	up
ENSBIXG00005020348	2.60518725	-0.8449607	9.45E-04	0.03797501	up

ENSBIXG00005003254	5.09907841	-1.4358901	9.61E-04	0.03797501	up
PB.5784(chr12: 14773515-14780548)	1.53959117	-0.1384862	9.62E-04	0.03797501	up
ENSBIXG00005001548	1.60847532	1.6011539	9.88E-04	0.03872677	up
ENSBIXG00005016693	1.03593683	1.78381692	9.98E-04	0.03882972	up
PB.21752(chr22: 54777397-54780881)	1.91609719	-1.6885546	0.00101564	0.03925049	up
ENSBIXG00005025825	1.42757463	-0.4673236	0.00103838	0.0393374	up
ENSBIXG00005027935	1.29537627	-0.4933815	0.00104457	0.0393374	up
ENSBIXG00005021852	1.02961881	5.60959838	0.00106264	0.03952377	up
PB.13810(chr18: 15223903-15229922)	2.90635779	-0.3909209	0.00106436	0.03952377	up
ENSBIXG00005012913	2.69902054	2.39499047	0.00108658	0.0400284	up
ENSBIXG00005001617	1.41666729	-0.4967164	0.00111536	0.04043432	up
ENSBIXG00005019147	1.74389372	-0.4807095	0.00112184	0.04047183	up
ENSBIXG00005010444	1.66602647	4.76519483	0.00112609	0.04047183	up
ENSBIXG00005018089	3.16456683	2.05185712	0.00113513	0.04047183	up
ENSBIXG00005028831	1.21849038	0.79659111	0.00116298	0.04098187	up
ENSBIXG00005021398	1.02350192	0.66700842	0.00118306	0.0415108	up
ENSBIXG00005004914	2.96096329	1.76918232	0.0012273	0.04213847	up
ENSBIXG00005028674	1.01690309	1.48892002	0.00127066	0.04284171	up
ENSBIXG00005013391	1.68497213	-1.4665057	0.00145418	0.0457356	up
ENSBIXG00005012867	1.46034708	-1.0687766	0.00153996	0.04686231	up
ENSBIXG00005013467	1.07342854	4.08444618	0.00156325	0.04716276	up
ENSBIXG00005023876	2.47338589	2.02224253	0.0015688	0.04716276	up
ENSBIXG00005001428	2.35917523	2.80793886	0.00162247	0.04829248	up

ENSBIXG00005030718	1.49198627	6.27443825	0.00162408	0.04829248	up
ENSBIXG00005025774	1.77157089	-1.5560505	0.00162979	0.04829248	up
ENSBIXG00005027705	-3.6431729	0.64588059	3.22E-08	4.36E-04	down
ENSBIXG00005001381	-5.0507327	2.43871178	6.42E-08	4.36E-04	down
ENSBIXG00005003959	-2.7161875	4.45800646	2.90E-07	9.00E-04	down
ENSBIXG00005000145	-1.5434889	4.86178811	3.04E-07	9.00E-04	down
ENSBIXG00005006062	-1.3269582	6.4172037	4.39E-07	9.00E-04	down
ENSBIXG00005007934	-5.6095436	-2.95E-05	7.39E-07	0.00125356	down
ENSBIXG00005022407	-3.4638981	-0.0136183	7.89E-07	0.00125356	down
ENSBIXG00005008938	-5.5436272	-1.3096836	8.49E-07	0.00125356	down
ENSBIXG00005004010	-3.1715592	8.15091896	1.01E-06	0.00125356	down
ENSBIXG00005020160	-2.814905	3.09985615	1.07E-06	0.00125356	down
ENSBIXG00005017813	-3.4067291	0.99671896	1.26E-06	0.00125356	down
ENSBIXG00005010700	-1.5411271	4.72025158	1.30E-06	0.00125356	down
PB.26206(chr27: 38634482-38736901)	-3.648319	2.15680515	1.42E-06	0.00129444	down
ENSBIXG00005012399	-1.4089	4.75446699	2.06E-06	0.00159067	down
PB.37997(chr8: 49186981-49209542)	-3.4392692	-0.7388019	2.13E-06	0.00159067	down
ENSBIXG00005000868	-2.9232828	2.49206428	2.14E-06	0.00159067	down
ENSBIXG00005024637	-4.7413352	0.00863471	2.30E-06	0.00163995	down
ENSBIXG00005030462	-2.0188639	1.93862272	2.72E-06	0.00185795	down
ENSBIXG00005027206	-3.4873881	1.55324957	2.96E-06	0.00194138	down
ENSBIXG00005025471	-1.3031208	3.31164901	3.12E-06	0.00196753	down
ENSBIXG00005004054	-4.2207539	-1.8122908	3.33E-06	0.00202143	down

ENSBIXG00005008929	-3.0581706	-0.5204827	5.17E-06	0.00302337	down
ENSBIXG00005006455	-3.8374676	-1.7565213	5.59E-06	0.00315871	down
ENSBIXG00005018356	-3.4025512	-0.7273822	6.17E-06	0.00315958	down
ENSBIXG00005005756	-1.9087997	0.95876613	7.14E-06	0.0035095	down
PB.7729(chr13: 44493541-44510022)	-2.2444889	0.89140765	7.28E-06	0.0035095	down
ENSBIXG00005016082	-1.1982006	3.22340231	8.24E-06	0.00385817	down
ENSBIXG00005007913	-6.3274656	1.49619365	8.87E-06	0.00403842	down
PB.22638(chr23: 42274262-42278669)	-1.9226886	0.75726504	1.01E-05	0.00437238	down
ENSBIXG00005003855	-2.2250503	0.22276378	1.20E-05	0.00453185	down
ENSBIXG00005021328	-4.4990508	1.5014461	1.23E-05	0.00453185	down
ENSBIXG00005005579	-1.1186105	7.32032808	1.25E-05	0.00453185	down
ENSBIXG00005001503	-1.6117577	1.80543211	1.40E-05	0.00487215	down
PB.38863(chr8: 110494075-110537768)	-1.9296395	3.00933632	1.47E-05	0.00502114	down
PB.19241(chr20: 62956333-62972405)	-1.8960702	-0.2961887	1.67E-05	0.00538345	down
ENSBIXG00005013614	-1.1155809	6.13096535	1.71E-05	0.00538345	down
ENSBIXG00005006320	-2.5630139	-0.2017691	2.06E-05	0.00604122	down
ENSBIXG00005024495	-1.2139525	2.01249292	2.47E-05	0.00681407	down
PB.32739(chr5: 37235044-37246727)	-2.6726472	-0.6335532	2.57E-05	0.00681407	down
ENSBIXG00005024440	-1.3967198	5.3201584	2.60E-05	0.00681407	down
ENSBIXG00005022057	-2.211454	-0.5324487	2.66E-05	0.00681407	down
ENSBIXG00005018347	-1.6774612	0.20830956	2.74E-05	0.00683439	down
ENSBIXG00005027021	-1.9678253	-0.401266	2.75E-05	0.00683439	down

ENSBIXG00005029164	-1.1599108	5.80683736	2.80E-05	0.00684456	down
ENSBIXG00005022874	-1.0358938	2.39260957	2.84E-05	0.00685472	down
ENSBIXG00005027690	-2.2941781	2.11728508	3.00E-05	0.00693268	down
PB.7890(chr13: 52555026-52877321)	-2.3833804	6.12620404	3.00E-05	0.00693268	down
ENSBIXG00005013636	-1.7975805	1.49102087	3.28E-05	0.00697253	down
ENSBIXG00005012422	-1.2108727	3.47259196	3.65E-05	0.00738955	down
ENSBIXG00005019601	-1.884625	6.41867657	3.75E-05	0.00750146	down
ENSBIXG00005012953	-1.9613448	-0.5424891	3.86E-05	0.00762259	down
ENSBIXG00005027678	-1.5628342	2.06391672	4.11E-05	0.00793051	down
ENSBIXG00005016265	-1.6521354	0.48013698	4.32E-05	0.00805666	down
PB.14530(chr18: 51999890-52006504)	-1.60094	-0.2352158	4.33E-05	0.00805666	down
ENSBIXG00005016335	-1.3069537	4.00936411	4.69E-05	0.00826031	down
PB.14589(chr18: 54423463-54430781)	-1.9289839	0.18592499	5.13E-05	0.00866566	down
ENSBIXG00005023367	-3.0087218	-0.7287379	6.60E-05	0.01052276	down
ENSBIXG00005000026	-3.3021572	-1.5703836	6.70E-05	0.01055505	down
ENSBIXG00005001551	-1.805119	2.14092511	6.98E-05	0.01066208	down
ENSBIXG00005027822	-1.2509796	1.75008693	7.03E-05	0.01066208	down
ENSBIXG00005017503	-2.5323381	0.62595908	7.55E-05	0.01134769	down
ENSBIXG00005022779	-1.0125361	4.04902641	1.03E-04	0.01402806	down
ENSBIXG00005025372	-1.5477591	5.54512143	1.12E-04	0.01491371	down
PB.8608(chr14: 8133786-8150613)	-4.0508306	-1.0453028	1.14E-04	0.01491371	down
PB.42194(chrX: 127254957-127260754)	-1.0972186	0.78329835	1.29E-04	0.01622955	down

ENSBIXG00005027378	-1.1318535	3.3262508	1.43E-04	0.01729736	down
ENSBIXG00005015994	-1.0475767	6.39071661	1.45E-04	0.01742763	down
ENSBIXG00005010916	-1.3518724	7.31381192	1.49E-04	0.01751958	down
ENSBIXG00005015829	-1.4495457	4.10153406	1.58E-04	0.01806608	down
ENSBIXG00005013061	-2.3167176	1.79879727	1.63E-04	0.01828866	down
ENSBIXG00005009669	-2.9718568	0.91665405	1.68E-04	0.01862049	down
ENSBIXG00005028463	-1.1152489	3.42512243	1.68E-04	0.01862049	down
ENSBIXG00005014426	-1.3260153	1.38866734	1.71E-04	0.01877835	down
PB.20615(chr22: 5534177-5542770)	-2.2119555	1.68567846	1.84E-04	0.01921619	down
ENSBIXG00005014504	-1.5484749	4.27838781	1.88E-04	0.01950894	down
ENSBIXG00005008640	-1.271922	-0.1035469	1.95E-04	0.0197843	down
ENSBIXG00005031220	-1.5032841	-0.2525677	1.97E-04	0.01984799	down
ENSBIXG00005002468	-1.0433597	3.66771353	2.01E-04	0.01988389	down
ENSBIXG00005015655	-5.001567	-2.72782	2.08E-04	0.02015108	down
ENSBIXG00005008786	-1.6684297	5.61970317	2.18E-04	0.0207486	down
ENSBIXG00005009088	-1.9774759	3.76797939	2.38E-04	0.02179322	down
ENSBIXG00005010889	-1.1590903	2.89973645	2.41E-04	0.02179613	down
PB.28883(chr3: 50268075-50272432)	-1.5444259	0.43200847	2.43E-04	0.02179613	down
ENSBIXG00005016589	-1.8148473	2.75568293	2.43E-04	0.02179613	down
ENSBIXG00005010329	-1.209169	4.0666022	2.53E-04	0.02203663	down
ENSBIXG00005030493	-1.2461305	3.56872643	2.61E-04	0.02205766	down
ENSBIXG00005007140	-1.5154155	2.87733916	2.69E-04	0.02218873	down
ENSBIXG00005030575	-1.2694198	2.78919024	2.80E-04	0.02250496	down

ENSBIXG00005008806	-1.1797238	2.77584571	2.87E-04	0.02266956	down
ENSBIXG00005026125	-2.0090082	2.84676273	3.05E-04	0.02327347	down
PB.15513(chr19: 32970795-33019431)	-1.5776838	3.05471905	3.21E-04	0.02398673	down
ENSBIXG00005028913	-1.0403672	2.48707035	3.22E-04	0.02398673	down
ENSBIXG00005024908	-1.3822982	1.52411305	3.49E-04	0.02432878	down
ENSBIXG00005012468	-4.574842	-2.0422478	3.50E-04	0.02432878	down
ENSBIXG00005012257	-1.2567541	1.79877286	3.50E-04	0.02432878	down
PB.30968(chr4: 47029330-47316079)	-1.4521794	4.21445274	3.50E-04	0.02432878	down
ENSBIXG00005028123	-1.5438532	-0.3947404	3.70E-04	0.02488603	down
ENSBIXG00005016091	-1.7658849	2.30244175	3.70E-04	0.02488603	down
ENSBIXG00005009668	-1.3162031	0.09200309	3.71E-04	0.02488603	down
PB.27935(chr29: 50057479-50061456)	-1.5040078	1.04258666	3.93E-04	0.02582515	down
ENSBIXG00005015701	-1.2330869	1.57708244	4.15E-04	0.02627617	down
ENSBIXG00005025942	-1.1188704	0.80110932	4.28E-04	0.02680202	down
ENSBIXG00005028342	-1.1621284	0.51567362	4.52E-04	0.0277068	down
ENSBIXG00005002017	-2.9093228	2.19804686	4.58E-04	0.0277068	down
ENSBIXG00005002395	-1.3579088	3.98432478	4.77E-04	0.02821695	down
ENSBIXG00005028729	-1.1282827	0.45683212	4.82E-04	0.02830066	down
ENSBIXG00005008106	-1.0535125	4.56057587	4.90E-04	0.02859418	down
PB.40246(chr9: 103651342-103661362)	-1.5008095	1.11350324	4.99E-04	0.02897219	down
ENSBIXG00005030641	-1.121533	-0.9103875	5.02E-04	0.02897219	down
ENSBIXG00005030199	-1.4409916	0.2402639	5.07E-04	0.02908572	down

ENSBIXG00005025087	-3.7809866	-1.2851701	5.14E-04	0.02923176	down
ENSBIXG00005019755	-2.0555892	1.36773789	5.51E-04	0.03021461	down
ENSBIXG00005017403	-1.420539	5.29872246	5.57E-04	0.03027457	down
ENSBIXG00005013097	-1.0746149	2.09433058	5.78E-04	0.03105873	down
ENSBIXG00005026538	-1.4490164	1.18535686	5.83E-04	0.0311237	down
ENSBIXG00005030594	-1.1188468	2.84172323	5.95E-04	0.03133771	down
ENSBIXG00005031209	-1.0437549	1.27513119	6.38E-04	0.03242645	down
ENSBIXG00005009451	-2.280431	0.54443593	6.60E-04	0.03299835	down
ENSBIXG00005016535	-1.6086871	-0.3454163	6.61E-04	0.03299835	down
PB.26550(chr28: 14577133-14590494)	-1.6754307	1.96947712	6.68E-04	0.03314616	down
ENSBIXG00005011205	-1.3295596	-1.3718436	6.99E-04	0.03379464	down
ENSBIXG00005022978	-1.4477038	4.99371564	7.03E-04	0.03379464	down
ENSBIXG00005027280	-2.8262687	-1.4825214	7.30E-04	0.03426827	down
ENSBIXG00005021686	-3.7610006	3.14214229	7.59E-04	0.03551634	down
ENSBIXG00005022303	-1.0557865	2.96830486	8.04E-04	0.03648729	down
ENSBIXG00005026000	-1.8728217	-0.7677796	8.06E-04	0.03648729	down
PB.24210(chr25: 16200135-16214902)	-1.1356238	-0.4848831	8.42E-04	0.03673627	down
ENSBIXG00005027231	-3.0244235	6.60262533	9.52E-04	0.03797501	down
ENSBIXG00005007217	-1.5504092	-0.041679	9.60E-04	0.03797501	down
ENSBIXG00005028103	-1.1828414	-0.1297917	0.00103779	0.0393374	down
ENSBIXG00005008290	-1.0979601	-1.1919513	0.0010539	0.0393374	down
ENSBIXG00005029012	-1.0655409	0.17804029	0.00106612	0.03952377	down
ENSBIXG00005027885	-1.3946211	-0.1579842	0.00113032	0.04047183	down

PB.27217(chr29: 779683-784625)	-1.3500422	0.89378216	0.00113274	0.04047183	down
ENSBIXG00005014754	-1.3687304	0.05735599	0.00117496	0.04131535	down
ENSBIXG00005018559	-1.4382428	1.26566803	0.00122041	0.04213847	down
ENSBIXG00005013176	-1.2403132	0.17285382	0.00123377	0.04213847	down
ENSBIXG00005013518	-1.0052287	2.72875926	0.00124512	0.04232892	down
ENSBIXG00005006445	-1.2395862	1.96279756	0.00128625	0.0431013	down
ENSBIXG00005025138	-1.0861373	1.34413317	0.00138718	0.04492149	down
PB.30013(chr3: 114107624-114143716)	-1.5304551	-0.1650219	0.00139315	0.04502603	down
ENSBIXG00005018877	-1.5840415	1.63243159	0.00140004	0.04514754	down
ENSBIXG00005001549	-1.0641652	-0.4027857	0.00142406	0.04556886	down
ENSBIXG00005030566	-1.025452	-1.1934268	0.00143103	0.0456204	down
ENSBIXG00005025476	-1.0433002	1.52612508	0.00144461	0.0457356	down
ENSBIXG00005021226	-1.6112403	1.32908817	0.0014667	0.04586002	down
ENSBIXG00005000364	-1.2161809	0.43678023	0.00147444	0.04586002	down
ENSBIXG00005003901	-1.6964025	-0.2672041	0.00147718	0.04586002	down
PB.9412(chr14: 77181034-77187951)	-2.7796524	-0.5324845	0.00149361	0.04609081	down
ENSBIXG00005028048	-2.8847164	0.59643924	0.00154149	0.04686231	down
ENSBIXG00005027084	-2.8464941	0.28944736	0.00156477	0.04716276	down
ENSBIXG00005025682	-1.1898049	1.62366765	0.00157152	0.04716276	down
ENSBIXG00005004915	-1.0080359	1.29102734	0.00157768	0.04722057	down
ENSBIXG00005021512	-2.3914448	0.58045179	0.00165714	0.048688	down
ENSBIXG00005028050	-1.4598377	-0.0998488	0.001658	0.048688	down
ENSBIXG00005019370	-1.5781197	0.60255181	0.00166453	0.04879246	down

ENSBIXG00005022833	-1.2884707	1.44670141	0.00166799	0.04880652	down
ENSBIXG00005028295	-1.0268199	0.19948697	0.00168927	0.04915413	down
ENSBIXG00005019829	-1.8488627	3.24305516	0.00169433	0.04915413	down
ENSBIXG00005027569	-1.0862068	1.05891451	0.00169821	0.04915413	down
ENSBIXG00005027906	-1.0370855	-0.1258393	0.00171485	0.04915413	down
PB.31587(chr4: 85517588-85717614)	-1.136329	5.30820638	0.00172932	0.04928101	down
ENSBIXG00005024482	-1.3040683	0.18678059	0.00173313	0.04930386	down

Table S3. DEGs identified by Iso-RNA technology using sleuth with p-value <0.05.

Gene ID	No of Aggregated Transcripts	P-value	Q-value	Up or downregulated in Angus
PB.10855(chr16:5537662-6117426)	97	3.21E-49	4.51E-45	up
PB.22495(chr23:32998699-33305328)	49	1.22E-12	3.81E-09	up
PB.6554(chr12:71818122-72095198)	23	5.40E-12	1.26E-08	up
ENSBIXG00005021755	13	1.34E-11	2.89E-08	up
ENSBIXG00005015588	35	5.02E-11	9.80E-08	up
ENSBIXG00005007178	21	1.55E-10	2.41E-07	up
ENSBIXG00005011873	4	6.94E-10	8.47E-07	up
ENSBIXG00005001800	33	8.01E-10	9.00E-07	up
ENSBIXG00005028377	7	1.94E-09	2.10E-06	up
ENSBIXG00005008599	43	3.12E-09	3.03E-06	up

ENSBIXG00005027604	12	6.41E-09	6.00E-06	up
ENSBIXG00005002297	1	1.25E-08	1.13E-05	up
PB.8232(chr13:72343034-72739013)	48	3.89E-08	3.12E-05	up
PB.37807(chr8:38483047-38759945)	12	4.41E-08	3.44E-05	up
ENSBIXG00005012354	6	1.30E-07	9.63E-05	up
PB.4998(chr11:70632510-70657558)	34	2.19E-07	1.46E-04	up
ENSBIXG00005020385	36	5.57E-07	3.48E-04	up
PB.32119(chr5:6971631-6989332)	41	6.39E-07	3.82E-04	up
ENSBIXG00005008345	33	8.46E-07	4.85E-04	up
PB.29663(chr3:99563603-99633697)	45	1.03E-06	5.79E-04	up
ENSBIXG00005022063	31	1.87E-06	9.99E-04	up
PB.34225(chr6:22280211-22432196)	15	1.92E-06	9.99E-04	up
PB.35018(chr6:88097650-88206360)	78	1.92E-06	9.99E-04	up
ENSBIXG00005004538	27	2.02E-06	0.00102912	up
ENSBIXG00005001387	18	2.06E-06	0.00103154	up
PB.41124(chrX:Inf--Inf)	2	3.96E-06	0.00182406	up
ENSBIXG00005010381	23	7.32E-06	0.00311707	up
ENSBIXG00005001405	13	7.33E-06	0.00311707	up

ENSBIXG00005010189	23	1.34E-05	0.00512999	up
ENSBIXG00005006815	11	1.35E-05	0.00512999	up
PB.24961(chr26:8215826-8302896)	34	1.59E-05	0.00587321	up
PB.35014(chr6:87912339-88086954)	156	1.89E-05	0.00680506	up
ENSBIXG00005019414	43	2.02E-05	0.00718033	up
ENSBIXG00005027600	1	2.07E-05	0.00727429	up
PB.38791(chr8:102944402-102957561)	8	2.40E-05	0.00830776	up
ENSBIXG00005010669	46	3.16E-05	0.01067279	up
ENSBIXG00005000548	48	3.43E-05	0.01132645	up
ENSBIXG00005008859	8	3.64E-05	0.01181116	up
ENSBIXG00005006456	7	3.71E-05	0.01182383	up
ENSBIXG00005007343	3	4.25E-05	0.01298388	up
ENSBIXG00005029121	2	4.94E-05	0.01429893	up
ENSBIXG00005002619	6	5.51E-05	0.0155122	up
ENSBIXG00005015502	16	5.53E-05	0.0155122	up
PB.20446(chr22:162727-163808)	2	5.89E-05	0.01636945	up
ENSBIXG00005014432	20	6.34E-05	0.01710955	up
ENSBIXG00005020810	2	6.60E-05	0.01765205	up
PB.13747(chr18:13477881-13533644)	30	6.89E-05	0.01824429	up

ENSBIXG00005024698	24	7.10E-05	0.01863057	up
PB.16479(chr19:63575740-63609965)	40	7.43E-05	0.0192744	up
ENSBIXG00005017436	8	7.48E-05	0.0192744	up
ENSBIXG00005018683	11	7.84E-05	0.01992936	up
ENSBIXG00005008295	15	1.05E-04	0.02602164	up
PB.17597(chr2:89854055-89893530)	43	1.06E-04	0.02602164	up
PB.36408(chr7:69390189-69392416)	18	1.35E-04	0.03235371	up
ENSBIXG00005009756	8	1.37E-04	0.03235371	up
ENSBIXG00005014542	13	1.37E-04	0.03235371	up
ENSBIXG00005001102	46	1.41E-04	0.03278731	up
ENSBIXG00005029757	14	1.67E-04	0.03750985	up
ENSBIXG00005005354	25	1.69E-04	0.03759831	up
PB.8231(chr13:72399949-72713627)	11	1.73E-04	0.03831276	up
PB.32967(chr5:52069922-52302837)	17	1.76E-04	0.03855406	up
PB.20287(chr21:65548753-65548235)	1	1.79E-04	0.03858554	up
ENSBIXG00005016922	18	1.93E-04	0.04095777	up
ENSBIXG00005010659	14	1.97E-04	0.04151294	up
ENSBIXG00005013789	4	2.13E-04	0.04437351	up

ENSBIXG00005021219	8	2.24E-04	0.04568067	up
ENSBIXG00005005823	22	2.41E-04	0.04756864	up
ENSBIXG00005013769	38	2.45E-04	0.04807167	up
ENSBIXG00005029811	1	2.51E-04	0.04859066	up
ENSBIXG00005004010	40	1.32E-63	3.71E-59	down
PB.16032(chr19:49267630-49357766)	34	1.44E-21	1.35E-17	down
ENSBIXG00005019601	13	1.07E-17	7.52E-14	down
PB.16446(chr19:62408309-62529375)	54	4.36E-17	2.45E-13	down
ENSBIXG00005022489	15	4.05E-14	1.90E-10	down
ENSBIXG00005001381	12	7.81E-14	3.13E-10	down
ENSBIXG00005005332	26	6.83E-13	2.40E-09	down
ENSBIXG00005006062	21	1.48E-12	4.15E-09	down
ENSBIXG00005030810	27	2.51E-12	6.42E-09	down
ENSBIXG00005003152	21	5.23E-11	9.80E-08	down
ENSBIXG00005015315	4	1.03E-10	1.80E-07	down
ENSBIXG00005007542	51	1.40E-10	2.31E-07	down
ENSBIXG00005027705	5	2.03E-10	3.00E-07	down
PB.19328(chr21:2109716-2141036)	7	2.15E-10	3.01E-07	down
PB.9513(chr15:1743877-1891946)	34	3.18E-10	4.25E-07	down
ENSBIXG00005027231	12	3.33E-10	4.25E-07	down

PB.19319(chr21:1973995-1979927)	34	7.45E-10	8.71E-07	down
PB.938(chr1:80194050-80341962)	220	2.40E-09	2.47E-06	down
ENSBIXG00005024440	11	2.46E-09	2.47E-06	down
PB.39994(chr9:87165774-87341542)	12	1.52E-08	1.33E-05	down
ENSBIXG00005016748	26	2.79E-08	2.32E-05	down
PB.27139(chr28:44228544-44335452)	39	6.04E-08	4.59E-05	down
ENSBIXG00005011180	18	1.64E-07	1.18E-04	down
ENSBIXG00005027701	10	1.73E-07	1.21E-04	down
PB.14631(chr18:57211453-57284650)	23	2.10E-07	1.44E-04	down
ENSBIXG00005005974	6	2.30E-07	1.50E-04	down
ENSBIXG00005011224	26	2.49E-07	1.59E-04	down
ENSBIXG00005008786	8	5.84E-07	3.56E-04	down
ENSBIXG00005013614	35	6.57E-07	3.84E-04	down
ENSBIXG00005008643	19	1.50E-06	8.23E-04	down
ENSBIXG00005020160	4	2.09E-06	0.00103154	down
ENSBIXG00005007141	12	2.33E-06	0.00112889	down
PB.31848(chr4:107690452-107691441)	52	2.66E-06	0.0012651	down
ENSBIXG00005010835	27	5.15E-06	0.00233195	down

ENSBIXG00005011852	23	6.32E-06	0.00281836	down
ENSBIXG00005006225	19	6.75E-06	0.00296036	down
ENSBIXG00005021285	7	7.75E-06	0.00324862	down
ENSBIXG00005003637	1	8.12E-06	0.00335401	down
ENSBIXG00005028769	24	8.73E-06	0.00355281	down
ENSBIXG00005030486	8	1.05E-05	0.00420662	down
ENSBIXG00005024769	10	1.25E-05	0.00494304	down
ENSBIXG00005003661	38	1.32E-05	0.00512999	down
PB.7890(chr13:52705508-52877219)	3	1.42E-05	0.00533015	down
ENSBIXG00005005876	4	1.66E-05	0.00606831	down
ENSBIXG00005005187	14	3.07E-05	0.01052662	down
ENSBIXG00005027255	5	3.28E-05	0.0109615	down
PB.38120(chr8:59957431-59957977)	3	3.66E-05	0.01181116	down
ENSBIXG00005000124	18	3.87E-05	0.01222256	down
ENSBIXG00005012399	13	4.02E-05	0.01253006	down
ENSBIXG00005006159	1	4.35E-05	0.01311817	down
PB.38870(chr8:110824021-110879086)	3	4.45E-05	0.01322787	down
ENSBIXG00005019708	21	4.48E-05	0.01322787	down
ENSBIXG00005004586	2	4.90E-05	0.01429893	down
ENSBIXG00005014873	1	5.42E-05	0.0155122	down

ENSBIXG00005019049	1	5.97E-05	0.01643615	down
ENSBIXG00005017376	7	7.88E-05	0.01992936	down
ENSBIXG00005016242	6	1.04E-04	0.02602164	down
ENSBIXG00005003180	5	1.21E-04	0.02951297	down
ENSBIXG00005007378	21	1.31E-04	0.03162387	down
PB.15310(chr19:26275476-26341578)	53	1.41E-04	0.03278731	down
ENSBIXG00005006330	4	1.43E-04	0.03281697	down
ENSBIXG00005017341	25	1.44E-04	0.03281697	down
ENSBIXG00005011866	1	1.60E-04	0.03632798	down
ENSBIXG00005030565	1	1.78E-04	0.03858554	down
ENSBIXG00005010543	2	1.80E-04	0.03858554	down
ENSBIXG00005015994	26	2.11E-04	0.0441618	down
ENSBIXG00005013076	12	2.18E-04	0.04510633	down
ENSBIXG00005007934	1	2.25E-04	0.04568067	down
PB.26206(chr27:38716949-38716260)	5	2.32E-04	0.04676969	down
ENSBIXG00005022067	1	2.37E-04	0.04712807	down
ENSBIXG00005015655	1	2.51E-04	0.04859066	down
ENSBIXG00005029561	1	2.57E-04	0.04945964	down

Table S4. DETs identified by Iso-Seq technology using limma Linear model with p-value <0.05.

Transcript ID (CDS chr:start-end)	logFC	log2 Average	P-value	Q-value	Up or downregulat

		Expression			ed in Angus
PB.35887.89(chr7:42578060-42572956)	9.17469233	1.03414053	4.51E-06	0.02371186	up
PB.32937.63(chr5:50551573-50526721)	3.73663893	2.85479696	9.82E-06	0.03287655	up
PB.10073.13(chr15:38866633-39020852)	3.98738444	3.70975295	1.05E-05	0.03348933	up
PB.17402.5(chr2:70935364-70984504)	2.52681848	4.34872654	1.57E-05	0.03597122	up
PB.38862.6(chr8:110399933-110376009)	3.70051049	2.00977042	1.80E-05	0.03733984	up
PB.37807.116(chr8:38724276-38749773)	3.78294891	2.23268704	2.28E-05	0.03733984	up
ENSBIXT00005041568	3.72516352	2.81401882	2.37E-05	0.03733984	up
PB.16479.202(chr19:63612006-63599416)	3.38343521	2.96669378	2.52E-05	0.03733984	up
PB.37807.117(chr8:38734624-38749773)	2.83708776	3.6956437	3.05E-05	0.03733984	up
ENSBIXT00005002166	2.26155082	5.89840992	2.77E-05	0.03733984	up
PB.41850.107(chrX:89440766-89377399)	3.35772218	7.28325999	3.23E-05	0.03733984	up
PB.11287.308(chr16:41918357-41929734)	6.86678808	-0.0627655	2.34E-05	0.03733984	up
PB.33571.63(chr5:NON_CODING)	6.7363331	-0.1385971	2.40E-05	0.03733984	up

PB.11287.194(chr16:41915419-41917739)	7.26755154	0.14962124	2.60E-05	0.03733984	up
PB.4790.38(chr11:49285202-49258804)	7.0057868	-0.0617781	2.83E-05	0.03733984	up
PB.17180.18(chr2:48097949-48148359)	7.03926868	0.66160158	3.03E-05	0.03733984	up
PB.14576.48(chr18:53957143-53940247)	7.19323716	0.06908795	3.01E-05	0.03733984	up
PB.4692.50(chr11:44549009-44541722)	6.57284988	-0.2485325	3.36E-05	0.03812666	up
PB.28297.100(chr3:16277111-16255521)	5.25733253	3.69039789	3.68E-05	0.03877928	up
PB.27929.2(chr29:49586313-49548100)	1.9966966	4.33901844	3.63E-05	0.03877928	up
PB.32432.2(chr5:16648461-16658035)	3.87884769	7.21937847	3.96E-05	0.03877928	up
PB.36156.92(chr7:58985329-58983348)	6.54484838	-0.22805	3.62E-05	0.03877928	up
PB.27455.10(chr29:7836143-7833401)	7.70785117	1.4733367	4.07E-05	0.03877928	up
PB.9533.6(chr15:2885344-2866888)	1.92730698	3.9233806	4.97E-05	0.03943862	up
ENSBIXT00005016490	2.94920613	3.57069019	5.11E-05	0.03943862	up
ENSBIXT00005009640	2.68743496	4.51271392	5.12E-05	0.03943862	up
PB.31492.6(chr4:75157807-	1.99834379	3.8952841	5.34E-05	0.03943862	up

75152995)					
ENSBIXT00005008825	2.04757046	6.63119627	5.43E-05	0.03943862	up
PB.32577.1(chr5:25946234-25870710)	1.92920961	6.25525771	5.71E-05	0.03943862	up
PB.11238.77(chr16:40000289-39992002)	6.32202766	-0.3468573	4.48E-05	0.03943862	up
PB.2253.60(chr10:8811018-8795070)	7.56488699	0.24937532	4.68E-05	0.03943862	up
PB.30102.25(chr3:119951603-119954436)	6.57305662	-0.2459518	4.68E-05	0.03943862	up
PB.38862.47(chr8:110399933-110374648)	6.24309601	-0.3650502	5.55E-05	0.03943862	up
PB.5092.63(chr11:73682558-73670151)	7.28580681	0.06027485	5.69E-05	0.03943862	up
PB.35034.5(chr6:89421068-89546130)	2.62205963	2.37077389	6.06E-05	0.04031333	up
PB.9356.3(chr14:70410373-70472652)	2.60087108	3.43028762	6.20E-05	0.04031333	up
PB.11287.301(chr16:41918357-41929696)	8.1903313	0.67045899	6.19E-05	0.04031333	up
PB.938.669(chr1:80299730-80293650)	2.32948873	3.31565246	7.20E-05	0.04085865	up
ENSBIXT00005047399	2.57577333	7.18095352	7.20E-05	0.04085865	up
PB.20974.3(chr22:17704661-17685401)	7.51499402	1.00851263	6.55E-05	0.04085865	up

PB.41124.8(chrX:NON_CODING)	6.23961346	-0.429075	6.78E-05	0.04085865	up
PB.2428.39(chr10:NON_CODING)	6.02546238	-0.5086663	6.77E-05	0.04085865	up
PB.36841.5(chr7:91301898-91300930)	6.74786901	0.47730325	7.41E-05	0.04085865	up
PB.938.291(chr1:80254381-80245909)	3.01029595	1.73841702	8.03E-05	0.0416508	up
PB.32432.1(chr5:16648774-16658035)	3.73365137	3.23433706	7.93E-05	0.0416508	up
PB.34736.6(chr6:58766461-58756887)	1.71323127	4.00711653	8.21E-05	0.0416508	up
PB.32371.1(chr5:14276109-14298886)	1.61894391	5.41447129	8.14E-05	0.0416508	up
PB.32432.28(chr5:16654066-16658035)	2.50070961	3.20504	8.47E-05	0.04229696	up
PB.4790.32(chr11:49285202-49258804)	5.76047376	-0.6550251	8.83E-05	0.0428298	up
PB.19600.57(chr21:21828729-21818127)	6.20794074	-0.4762865	9.44E-05	0.0435763	up
PB.27049.119(chr28:35648996-35634229)	5.30272859	2.69460712	9.70E-05	0.04417849	up
PB.14259.10(chr18:31154370-31108242)	1.360485	6.37980135	9.99E-05	0.04457377	up
PB.34141.53(chr6:15816002-15866182)	2.8058888	2.29927275	1.02E-04	0.04468188	up
PB.32432.15(chr5:16654066-	3.1505113	1.9103662	1.09E-04	0.0459440	up

16658035)	9	5		3	
PB.13806.25(chr18:15026970-15008969)	3.1821731 8	2.7412421 6	1.19E-04	0.0479548 9	up
PB.25828.6(chr27:15022628-15014234)	2.0748714 3	2.7663488 8	1.20E-04	0.0479548 9	up
ENSBIXT00005008594	2.8513337 9	2.9122724 9	1.22E-04	0.0479824 1	up
PB.22157.10(chr23:18055163-18054687)	5.9350012 1	- 0.5672414	1.25E-04	0.0482389 1	up
PB.6540.1(chr12:70657802-70923002)	2.6217054 8	2.1348422 6	1.31E-04	0.0490526 5	up
ENSBIXT00005047147	1.2558790 6	6.7534915 5	1.31E-04	0.0490526 5	up
PB.4790.126(chr11:49285202-49268360)	5.6208439 6	- 0.6970088	1.30E-04	0.0490526 5	up
PB.6828.21(chr12:86203889-86194230)	2.8646873 7	1.5623294	1.35E-04	0.0494344 2	up
PB.29537.3(chr3:92462520-92450035)	1.5301382 6	4.9195634 5	1.33E-04	0.0494344 2	up
PB.11287.299(chr16:41918357-41929696)	7.0520126 3	0.0453407 6	1.37E-04	0.0498439 6	up
PB.35887.71(chr7:42578060-42572956)	- 7.3632439	7.7696217 4	7.39E-08	0.0029427 8	down
PB.2635.36(chr10:30740448-30769846)	-12.07668	2.5221720 5	8.79E-08	0.0029427 8	down
PB.25298.209(chr26:30450563-30460737)	- 2.5220461	5.8959872 9	9.70E-07	0.0162261 1	down

PB.19319.15(chr21:NON_CODING)	-10.11093	1.53531931	7.42E-07	0.01622611	down
ENSBIXT00005015481	-4.1035251	3.18687101	1.90E-06	0.02089662	down
PB.19319.1(chr21:NON_CODING)	-7.7314324	2.56538166	2.10E-06	0.02089662	down
PB.36348.82(chr7:66997224-66989116)	-8.684486	0.84218494	2.19E-06	0.02089662	down
PB.41850.111(chrX:89440766-89386897)	-2.9800587	6.3592985	3.87E-06	0.02371186	down
PB.14835.31(chr19:10311396-10301216)	-3.0548758	3.06285755	5.23E-06	0.02371186	down
ENSBIXT00005051562	-4.5675291	1.99971364	3.91E-06	0.02371186	down
PB.20748.11(chr22:9759743-9792495)	-4.703573	2.07666664	5.67E-06	0.02371186	down
PB.9636.171(chr15:7310781-7296846)	-5.4570209	2.13636897	4.87E-06	0.02371186	down
PB.40235.37(chr9:102817859-102806869)	-7.8117981	0.41144587	3.33E-06	0.02371186	down
PB.20974.5(chr22:17704661-17685401)	-8.0352945	0.48330294	5.02E-06	0.02371186	down
PB.3807.7(chr10:97995423-98003076)	-9.285043	1.08204028	5.55E-06	0.02371186	down
PB.34736.14(chr6:58780414-58756887)	-4.0973496	4.80498811	7.15E-06	0.0281418	down
ENSBIXT00005034025	-4.8294468	2.41568251	8.10E-06	0.02856446	down

ENSBIXT00005026772	-10.699943	1.93125554	8.11E-06	0.02856446	down
PB.20664.6(chr22:7558168-7557101)	-8.1619835	0.5243417	1.12E-05	0.03417141	down
PB.30206.80(chr4:5328448-5304125)	-2.8217775	2.93011254	1.31E-05	0.03597122	down
PB.13594.9(chr18:NON_CODING)	-2.7566048	2.65369902	1.41E-05	0.03597122	down
ENSBIXT00005019331	-6.512617	1.92186063	1.58E-05	0.03597122	down
PB.3572.99(chr10:78451290-78489956)	-7.8579223	0.38814548	1.25E-05	0.03597122	down
PB.36348.84(chr7:66998204-66989116)	-7.6252964	0.31047697	1.40E-05	0.03597122	down
PB.36019.8(chr7:NON_CODING)	-6.8180753	-0.1169343	1.51E-05	0.03597122	down
PB.16863.24(chr2:27082588-27093737)	-7.0542293	0.05957923	1.61E-05	0.03597122	down
PB.2484.23(chr10:20552403-20550753)	-8.1848506	0.65023438	1.68E-05	0.03622744	down
PB.16155.165(chr19:52070980-52072931)	-2.7323295	2.5207462	1.93E-05	0.03733984	down
ENSBIXT00005050859	-3.4099944	2.56080683	2.21E-05	0.03733984	down
ENSBIXT00005003968	-3.4753403	2.36824799	2.54E-05	0.03733984	down
PB.32148.4(chr5:7608118-7576303)	-2.6165102	2.58848427	2.79E-05	0.03733984	down

PB.42344.4(chrX:140885512-140885811)	-3.138045	4.0186742 2	2.66E-05	0.0373398 4	down
PB.36348.22(chr7:66997224-66989116)	- 6.9489642	- 0.0651987	1.93E-05	0.0373398 4	down
PB.9668.16(chr15:8963965-8966879)	- 7.1895575	0.0571064 1	2.02E-05	0.0373398 4	down
PB.35887.157(chr7:42578060-42572956)	- 7.9771977	1.6561482 9	2.18E-05	0.0373398 4	down
PB.2484.3(chr10:20551866-20550753)	- 8.5811583	0.8018386 8	2.47E-05	0.0373398 4	down
PB.35014.89(chr6:88039353-88055202)	- 6.1770566	- 0.4212628	2.76E-05	0.0373398 4	down
PB.29663.80(chr3:99564583-99598509)	- 6.9714118	0.0295380 2	2.96E-05	0.0373398 4	down
PB.37437.52(chr8:7388413-7395948)	- 10.195829	1.6320704 8	3.24E-05	0.0373398 4	down
PB.20721.2(chr22:9185159-9186472)	-6.901335	0.0017541 8	3.11E-05	0.0373398 4	down
PB.2253.47(chr10:8811018-8795070)	- 8.6187447	0.7487751 3	3.22E-05	0.0373398 4	down
ENSBIXT00005044948	- 8.2676135	0.6982416 5	3.46E-05	0.0386112 4	down
PB.20783.108(chr22:10270049-10275095)	- 2.7643316	2.0599141 5	3.79E-05	0.0387792 8	down
PB.29663.54(chr3:99564583-99598509)	- 8.2905036	0.7244219 6	3.83E-05	0.0387792 8	down
ENSBIXT00005000672	-	2.2150893	4.01E-05	0.0387792	down

	9.9140682	5		8	
PB.22526.17(chr23:34225027-34225417)	-6.6967298	-0.0959219	3.82E-05	0.0387792 8	down
PB.2167.36(chr10:5219242-5206193)	-6.2636806	-0.3996169	4.02E-05	0.0387792 8	down
PB.2484.20(chr10:20551866-20550753)	-6.6423115	-0.1644743	4.11E-05	0.0387792 8	down
PB.8084.32(chr13:63379311-63364660)	-3.2606747	2.8211149	4.22E-05	0.0391907 7	down
PB.11003.204(chr16:28220265-28202674)	-2.4314121	2.4930576 2	4.85E-05	0.0394386 2	down
PB.13527.12(chr18:9267992-9253461)	-3.0544352	2.2653947 7	4.92E-05	0.0394386 2	down
PB.20750.17(chr22:9823794-9824757)	-2.5792629	2.8764689 3	4.86E-05	0.0394386 2	down
PB.22243.13(chr23:22145804-21949644)	-3.4636056	4.0660006 1	4.83E-05	0.0394386 2	down
ENSBIXT00005038318	-1.9387741	4.1375051 6	4.83E-05	0.0394386 2	down
PB.20750.55(chr22:9823794-9824757)	-2.8433865	2.9625993 8	5.64E-05	0.0394386 2	down
PB.28927.16(chr3:52038165-52015297)	-5.4235127	1.6979815 3	4.68E-05	0.0394386 2	down
PB.14445.23(chr18:49113739-49043923)	-6.2036055	-0.431449	4.71E-05	0.0394386 2	down
PB.29663.88(chr3:99564583-99598509)	-6.3547615	-0.2850771	4.74E-05	0.0394386 2	down

PB.32079.2(chr5:5883360-5832634)	-6.5689567	-0.2757768	5.48E-05	0.03943862	down
PB.30102.19(chr3:119956437-119962056)	-7.0063744	0.02536853	5.54E-05	0.03943862	down
ENSBIXT00005026300	-6.6287877	-0.221327	5.51E-05	0.03943862	down
PB.2484.13(chr10:20557023-20552326)	-6.7875378	-0.0680293	5.60E-05	0.03943862	down
PB.5953.31(chr12:21371640-21389731)	-6.3504584	-0.3776881	5.63E-05	0.03943862	down
ENSBIXT00005045982	-6.557593	0.62673131	6.10E-05	0.04031333	down
PB.26108.60(chr27:32932312-32929468)	-5.9110441	-0.5122605	6.07E-05	0.04031333	down
PB.26486.11(chr28:10274552-10347184)	-5.6172777	-0.6824819	6.15E-05	0.04031333	down
PB.30230.2(chr4:6418811-6414779)	-2.0955904	2.75354437	6.39E-05	0.04085865	down
PB.1439.16(chr1:111242242-111174568)	-1.8037332	4.34051328	6.97E-05	0.04085865	down
PB.6828.14(chr12:86211999-86196819)	-2.0389345	3.52021692	7.37E-05	0.04085865	down
ENSBIXT00005001323	-3.132311	3.79175826	7.45E-05	0.04085865	down
ENSBIXT00005053696	-2.1552594	3.90993287	7.29E-05	0.04085865	down
PB.16479.126(chr19:63612006-63604028)	-2.7760124	4.21372991	7.43E-05	0.04085865	down

PB.28297.76(chr3:16277111-16255521)	-1.999398	5.1700961 3	7.33E-05	0.0408586 5	down
PB.20615.2(chr22:5540603-5540980)	- 5.5126501	1.2348370 7	6.87E-05	0.0408586 5	down
PB.13408.152(chr18:4481829-4474664)	- 7.0795458	- 0.0369505	6.81E-05	0.0408586 5	down
PB.2236.20(chr10:7825431-7773253)	- 5.6546937	- 0.6929378	6.60E-05	0.0408586 5	down
PB.13866.11(chr18:16791915-16796698)	- 6.0133055	- 0.5039224	6.73E-05	0.0408586 5	down
PB.30102.3(chr3:119956437-119962056)	- 8.8069047	1.0049632 5	7.10E-05	0.0408586 5	down
PB.24629.17(chr25:38644799-38648054)	- 5.6926985	- 0.6298106	6.71E-05	0.0408586 5	down
PB.8526.38(chr14:1076709-1077791)	- 6.4892878	-0.210222	7.52E-05	0.0409009 4	down
ENSBIXT00005029675	-3.049765	3.7358916 3	7.64E-05	0.0412173 9	down
PB.36828.19(chr7:90991826-91004762)	- 2.1018521	3.3984988 9	7.80E-05	0.0416508	down
PB.4077.7(chr11:7060007-7078509)	- 6.9385004	- 0.1032508	7.91E-05	0.0416508	down
PB.20615.9(chr22:5540603-5540980)	- 5.9188067	- 0.5122399	8.11E-05	0.0416508	down
PB.15574.28(chr19:35415512-35423824)	- 5.9756253	- 0.5584424	8.17E-05	0.0416508	down
ENSBIXT00005037691	-	1.8773967	8.39E-05	0.0422448	down

	2.9319984	5		5	
PB.26077.93(chr27:32529019-32542157)	-2.1848383	2.53795354	8.67E-05	0.04259843	down
PB.5989.7(chr12:NON_CODING)	-6.3053614	-0.4126744	8.67E-05	0.04259843	down
PB.36900.43(chr7:92993974-93047719)	-5.615058	-0.6998364	8.72E-05	0.04259843	down
PB.6810.15(chr12:86079603-86085842)	-5.667619	-0.7042555	8.93E-05	0.04301189	down
PB.38798.336(chr8:103341865-103337672)	-3.0320099	2.02992497	9.28E-05	0.0435763	down
PB.36830.15(chr7:91035528-91025776)	-5.8916347	-0.4995593	9.26E-05	0.0435763	down
PB.41435.89(chrX:57244328-57226925)	-5.9134245	-0.4867323	9.37E-05	0.0435763	down
PB.6960.166(chr13:6202246-6235992)	-5.624009	-0.6629474	9.29E-05	0.0435763	down
PB.36348.119(chr7:66997224-66989116)	-5.751613	-0.6047852	9.40E-05	0.0435763	down
PB.31086.25(chr4:54147119-54255078)	-2.4051664	2.33786257	9.56E-05	0.04382251	down
PB.11562.116(chr16:54555118-54544642)	-2.6126987	2.38419624	9.81E-05	0.04438467	down
PB.6846.8(chr12:86504527-86498438)	-5.737788	0.02087685	9.92E-05	0.04456616	down
PB.1860.2(chr1:144168993-144152297)	-3.4053199	1.89201534	1.02E-04	0.04468188	down

PB.4077.6(chr11:7060007-7078509)	-6.8610889	-0.1462795	1.03E-04	0.04468188	down
ENSBIXT00005021866	-5.9445433	0.08051579	1.03E-04	0.04468188	down
PB.19376.7(chr21:5656953-5565738)	-5.5108627	-0.7675143	1.02E-04	0.04468188	down
PB.2484.2(chr10:20552403-20550753)	-6.2185829	-0.3756977	1.05E-04	0.04497588	down
PB.28118.11(chr3:9409729-9414121)	-5.340182	-0.8471729	1.05E-04	0.04497588	down
PB.5608.21(chr11:NON_CODING)	-6.0544397	-0.4395567	1.07E-04	0.04513895	down
PB.16258.114(chr19:55110838-55117827)	-5.7409744	-0.5982861	1.10E-04	0.04604821	down
PB.25828.30(chr27:NON_CODING)	-5.9456453	-0.4635385	1.14E-04	0.04729945	down
PB.15828.8(chr19:43078374-43065366)	-2.4077405	4.47828589	1.16E-04	0.04795489	down
PB.9308.6(chr14:67717561-67289170)	-5.6766297	-0.6348805	1.18E-04	0.04795489	down
PB.42344.5(chrX:140885512-140885811)	-5.7891372	-0.5951898	1.19E-04	0.04795489	down
PB.36348.72(chr7:66998204-66989116)	-5.3012237	-0.8431402	1.19E-04	0.04795489	down
PB.29663.116(chr3:99564583-99598509)	-8.4795351	0.73480924	1.21E-04	0.04796879	down
PB.23131.7(chr24:33219019-	-6.579953	-0.145718	1.21E-04	0.0479687	down

33264811)				9	
ENSBIXT00005015038	-3.1640027	1.52560821	1.24E-04	0.04823891	down
PB.29663.85(chr3:99564583-99598509)	-4.5151111	1.35735549	1.23E-04	0.04823891	down
PB.30132.9(chr3:120532589-120546469)	-5.5427923	-0.7761853	1.25E-04	0.04823891	down
PB.27081.19(chr28:41660347-41628806)	-2.7171041	2.67733502	1.30E-04	0.04905265	down
PB.25552.66(chr26:42580542-42620338)	-6.2740279	-0.4073467	1.31E-04	0.04905265	down
PB.6964.52(chr13:6265214-6244202)	-6.1641111	-0.35652	1.35E-04	0.04943442	down
PB.16258.15(chr19:55110838-55125598)	-5.2439403	-0.8481691	1.35E-04	0.04943442	down

Table S5. DETs identified by RNA-Seq technology using sleuth with p-value <0.05.

Transcript ID (CDS chr:start-end)	Chr	P-value	Q-value	Regulation in Angus
ENSBIXT00005019343.1	19	1.25E-08	9.82E-04	up
PB.19319.65(chr21:NON_CODING)	21	6.87E-08	0.00270408	up
PB.35887.71(chr7:42578060-42572956)	7	1.27E-09	1.99E-04	down
PB.35887.157(chr7:42578060-42572956)	7	2.76E-08	0.0014472	down
PB.35887.148(chr7:42578060-42572956)	7	1.11E-07	0.00350408	down

PB.2214.1(chr10:7104977-7100453)	10	1.83E-07	0.00480907	down
PB.11151.2(chr16:34583523-34514491)	16	5.00E-07	0.01123648	down
ENSBIXT00005026772.1	10	1.54E-06	0.03026355	down
PB.35887.58(chr7:42578060-42572956)	7	1.95E-06	0.0341517	down
PB.35887.137(chr7:42578060-42572956)	7	2.28E-06	0.03588643	down

Table S6. The p-values from Wilcoxon test of the significance of different multi-mapping levels between three groups (DEGs identified by RNAseq, DEGs identified by Iso-Seq, DEGs identified by both)

Sample	RNAseq_vs_Both	Isoseq_vs_Both	RNAseq_vs_Isoseq
Angus_7	0.15008243	0.002138762	7.38E-08
Angus_53	0.08005686	0.079623451	2.03E-06
Angus_60	0.15907523	0.015884967	6.35E-07
Brahman_22	0.44530593	0.001240485	2.55E-06
Brahman_65	0.26738805	0.004761729	3.12E-06
Brahman_99	0.24113059	0.007142374	7.62E-06

Table S7. The significant DTUs ($p < 0.05$) identified using Iso-Seq technology.

Gene ID	likelihood ratio statistics	degrees of freedom	p-value	adjust p-value
ENSBIXG00005020385	873.277626	27	1.42E-166	8.78E-163
ENSBIXG00005005332	534.320359	3	1.74E-115	5.39E-112
ENSBIXG00005015350	366.778334	1	9.41E-82	1.95E-78

ENSBIXG00005025666	326.943369	4	1.66E-69	2.58E-66
ENSBIXG00005026491	267.03212	6	9.36E-55	1.16E-51
ENSBIXG00005000563	210.645933	7	6.37E-42	6.58E-39
ENSBIXG00005007485	199.985429	5	2.86E-41	2.53E-38
ENSBIXG00005011661	200.417962	9	2.71E-38	2.10E-35
ENSBIXG00005022121	178.924227	4	1.27E-37	8.75E-35
ENSBIXG00005020694	227.271546	20	4.21E-37	2.61E-34
ENSBIXG00005013320	148.631619	3	5.20E-32	2.93E-29
ENSBIXG00005012188	190.168012	16	7.63E-32	3.94E-29
ENSBIXG00005028664	154.965065	10	3.54E-28	1.69E-25
ENSBIXG00005000323	176.961683	18	3.83E-28	1.70E-25
ENSBIXG00005009015	156.239485	11	7.89E-28	3.26E-25
ENSBIXG00005017338	115.407314	1	6.41E-27	2.48E-24
ENSBIXG00005011318	147.134963	11	5.73E-26	2.09E-23
ENSBIXG00005029828	130.457257	8	2.27E-24	7.84E-22
ENSBIXG00005006925	131.416793	12	3.21E-22	1.05E-19
ENSBIXG00005003856	111.820462	7	3.85E-21	1.19E-18
ENSBIXG00005021168	106.755051	6	9.74E-21	2.87E-18
ENSBIXG00005006359	90.691502	2	2.03E-20	5.71E-18
ENSBIXG00005007320	89.3746987	3	2.98E-19	8.05E-17
ENSBIXG00005006819	94.4856495	5	7.66E-19	1.98E-16
ENSBIXG00005001996	90.9043165	4	8.46E-19	2.10E-16
ENSBIXG00005024919	95.9687575	6	1.74E-18	4.14E-16
ENSBIXG00005014726	80.7390083	2	2.94E-18	6.74E-16
ENSBIXG00005006435	145.493969	27	3.36E-18	7.44E-16

ENSBIXG00005017326	86.483172	4	7.35E-18	1.57E-15
ENSBIXG00005002147	77.7422995	2	1.31E-17	2.63E-15
ENSBIXG00005002855	118.044232	16	1.30E-17	2.63E-15
ENSBIXG00005016389	77.2599178	2	1.67E-17	3.24E-15
ENSBIXG00005007178	89.2758531	6	4.28E-17	8.05E-15
ENSBIXG00005022038	89.2050066	6	4.43E-17	8.08E-15
ENSBIXG00005000680	80.7854796	4	1.19E-16	2.10E-14
ENSBIXG00005000548	136.531445	27	1.36E-16	2.34E-14
ENSBIXG00005002016	80.2494863	4	1.54E-16	2.58E-14
ENSBIXG00005018766	76.3183305	3	1.89E-16	3.08E-14
ENSBIXG00005006472	79.6234574	4	2.09E-16	3.33E-14
ENSBIXG00005029361	100.90065	12	3.71E-16	5.75E-14
ENSBIXG00005009822	92.6543804	9	4.77E-16	7.21E-14
ENSBIXG00005028510	65.7249646	1	5.18E-16	7.65E-14
ENSBIXG00005005056	115.515739	19	7.60E-16	1.10E-13
ENSBIXG00005023052	97.6760828	12	1.59E-15	2.24E-13
ENSBIXG00005010965	80.8669327	6	2.37E-15	3.26E-13
ENSBIXG00005030476	60.4715079	1	7.47E-15	1.01E-12
ENSBIXG00005024924	89.0583091	10	8.23E-15	1.09E-12
ENSBIXG00005020089	78.113605	6	8.76E-15	1.11E-12
ENSBIXG00005009097	78.147535	6	8.62E-15	1.11E-12
ENSBIXG00005013515	64.4141869	2	1.03E-14	1.28E-12
ENSBIXG00005005927	104.578719	17	1.25E-14	1.52E-12
ENSBIXG00005031360	132.64732	31	1.55E-14	1.85E-12
ENSBIXG00005015341	73.2982707	5	2.11E-14	2.46E-12

ENSBIXG00005026742	61.7979637	2	3.81E-14	4.37E-12
ENSBIXG00005029622	61.6176241	2	4.17E-14	4.70E-12
ENSBIXG00005024440	56.8606975	1	4.68E-14	5.18E-12
ENSBIXG00005014588	86.2979674	11	8.83E-14	9.60E-12
ENSBIXG00005001988	66.8754132	4	1.04E-13	1.11E-11
ENSBIXG00005009375	58.7369758	2	1.76E-13	1.85E-11
ENSBIXG00005001444	62.1918558	3	2.00E-13	2.03E-11
ENSBIXG00005002646	68.6357749	5	1.97E-13	2.03E-11
ENSBIXG00005001051	98.8961194	18	3.53E-13	3.53E-11
ENSBIXG00005013866	68.5764799	6	8.01E-13	7.88E-11
ENSBIXG00005018920	92.2210148	16	9.73E-13	9.43E-11
ENSBIXG00005014248	70.83875	7	1.00E-12	9.54E-11
ENSBIXG00005006815	50.7764531	1	1.04E-12	9.73E-11
ENSBIXG00005028474	65.06977	5	1.08E-12	1.00E-10
ENSBIXG00005007167	61.6767102	4	1.29E-12	1.17E-10
ENSBIXG00005023567	67.3346902	6	1.44E-12	1.29E-10
ENSBIXG00005008286	64.0010456	5	1.81E-12	1.60E-10
ENSBIXG00005008330	81.2841521	12	2.35E-12	2.04E-10
ENSBIXG00005031003	60.415688	4	2.37E-12	2.04E-10
ENSBIXG00005002082	68.7942353	7	2.59E-12	2.20E-10
ENSBIXG00005023663	48.8001779	1	2.83E-12	2.37E-10
ENSBIXG00005017862	75.8055022	10	3.32E-12	2.74E-10
ENSBIXG00005024905	48.0748331	1	4.10E-12	3.35E-10
ENSBIXG00005011367	76.8598426	11	5.95E-12	4.79E-10
ENSBIXG00005007534	63.3350678	6	9.43E-12	7.50E-10

ENSBIXG00005016748	45.7962021	1	1.31E-11	1.03E-09
ENSBIXG00005028528	81.302896	14	1.62E-11	1.26E-09
ENSBIXG00005010195	91.7005429	19	1.66E-11	1.27E-09
ENSBIXG00005023159	49.5352367	2	1.75E-11	1.32E-09
ENSBIXG00005009439	85.1762892	17	4.55E-11	3.40E-09
ENSBIXG00005016714	62.4539819	7	4.88E-11	3.60E-09
ENSBIXG00005006900	78.3757716	14	5.66E-11	4.13E-09
ENSBIXG00005013329	69.3129494	10	6.02E-11	4.34E-09
ENSBIXG00005010202	64.0365503	8	7.48E-11	5.33E-09
ENSBIXG00005015785	50.023999	3	7.90E-11	5.56E-09
ENSBIXG00005012297	55.9961071	5	8.14E-11	5.67E-09
ENSBIXG00005007412	52.3282624	4	1.18E-10	8.11E-09
ENSBIXG00005011852	41.1619949	1	1.40E-10	9.55E-09
ENSBIXG00005013563	51.5488262	4	1.71E-10	1.16E-08
ENSBIXG00005008649	48.097709	3	2.03E-10	1.35E-08
ENSBIXG00005008181	56.7535918	6	2.05E-10	1.35E-08
ENSBIXG00005008474	59.2632296	7	2.12E-10	1.38E-08
ENSBIXG00005031029	53.86408	5	2.24E-10	1.44E-08
ENSBIXG00005015098	63.9860209	9	2.27E-10	1.45E-08
ENSBIXG00005018464	61.5118659	8	2.35E-10	1.49E-08
ENSBIXG00005002246	47.3342062	3	2.95E-10	1.85E-08
ENSBIXG00005015815	55.7501732	6	3.27E-10	2.03E-08
ENSBIXG00005007871	60.5217243	8	3.68E-10	2.26E-08
ENSBIXG00005012185	64.942638	10	4.16E-10	2.51E-08
ENSBIXG00005031485	43.1961898	2	4.17E-10	2.51E-08

ENSBIXG00005021531	75.4415546	15	4.71E-10	2.81E-08
ENSBIXG00005030133	77.3180934	16	5.04E-10	2.98E-08
ENSBIXG00005021362	62.0268958	9	5.44E-10	3.19E-08
ENSBIXG00005029662	86.3415648	21	6.85E-10	3.97E-08
ENSBIXG00005014197	51.4381644	5	7.03E-10	4.04E-08
ENSBIXG00005028188	45.2718847	3	8.10E-10	4.61E-08
ENSBIXG00005011224	51.0857314	5	8.31E-10	4.68E-08
ENSBIXG00005004648	41.5481426	2	9.50E-10	5.31E-08
ENSBIXG00005026723	40.9888297	2	1.26E-09	6.96E-08
ENSBIXG00005029700	44.0566853	3	1.47E-09	8.06E-08
ENSBIXG00005018618	54.8634787	7	1.59E-09	8.63E-08
ENSBIXG00005011771	57.2518764	8	1.61E-09	8.67E-08
ENSBIXG00005003072	52.2666362	6	1.65E-09	8.74E-08
ENSBIXG00005028037	98.5784432	29	1.65E-09	8.74E-08
ENSBIXG00005014223	58.5086827	9	2.60E-09	1.36E-07
ENSBIXG00005023447	42.7950584	3	2.72E-09	1.42E-07
ENSBIXG00005001663	39.424991	2	2.75E-09	1.42E-07
ENSBIXG00005011764	47.8092238	5	3.89E-09	1.99E-07
ENSBIXG00005011552	55.0071465	8	4.40E-09	2.24E-07
ENSBIXG00005016658	52.5021587	7	4.65E-09	2.34E-07
ENSBIXG00005023404	41.2022948	3	5.92E-09	2.96E-07
ENSBIXG00005000254	33.8294117	1	6.02E-09	2.97E-07
ENSBIXG00005024897	41.1665666	3	6.03E-09	2.97E-07
ENSBIXG00005013614	44.0353244	4	6.31E-09	3.08E-07
ENSBIXG00005014443	48.7359873	6	8.42E-09	4.08E-07

ENSBIXG00005017036	65.9840505	14	1.02E-08	4.90E-07
ENSBIXG00005000966	52.8656396	8	1.14E-08	5.46E-07
ENSBIXG00005000757	63.0786399	13	1.47E-08	6.96E-07
ENSBIXG00005005328	49.8666379	7	1.53E-08	7.21E-07
ENSBIXG00005029478	51.8552492	8	1.79E-08	8.37E-07
ENSBIXG00005031552	83.2810454	24	1.81E-08	8.38E-07
ENSBIXG00005031472	38.8714931	3	1.85E-08	8.49E-07
ENSBIXG00005009520	38.7451682	3	1.97E-08	8.96E-07
ENSBIXG00005006486	41.5546647	4	2.06E-08	9.34E-07
ENSBIXG00005003580	35.1985535	2	2.27E-08	1.02E-06
ENSBIXG00005016993	46.4885163	6	2.37E-08	1.06E-06
ENSBIXG00005030508	69.5767127	17	2.55E-08	1.13E-06
ENSBIXG00005026203	55.1695475	10	2.94E-08	1.29E-06
ENSBIXG00005000787	34.603188	2	3.06E-08	1.34E-06
ENSBIXG00005030292	37.6507587	3	3.35E-08	1.45E-06
ENSBIXG00005011818	34.3945401	2	3.40E-08	1.46E-06
ENSBIXG00005028248	56.7225878	11	3.74E-08	1.60E-06
ENSBIXG00005007417	36.9148326	3	4.80E-08	2.04E-06
ENSBIXG00005027255	33.6467317	2	4.94E-08	2.08E-06
ENSBIXG00005013250	39.547172	4	5.37E-08	2.25E-06
ENSBIXG00005005564	36.2831922	3	6.52E-08	2.72E-06
ENSBIXG00005010241	33.0514429	2	6.65E-08	2.75E-06
ENSBIXG00005015955	28.9994067	1	7.24E-08	2.97E-06
ENSBIXG00005008137	32.7898236	2	7.58E-08	3.09E-06
ENSBIXG00005006965	41.0720321	5	9.07E-08	3.68E-06

ENSBIXG00005005065	28.4114902	1	9.81E-08	3.95E-06
ENSBIXG00005023844	32.2394626	2	9.98E-08	3.99E-06
ENSBIXG00005010886	45.5899809	7	1.05E-07	4.18E-06
ENSBIXG00005025814	47.4174807	8	1.28E-07	5.01E-06
ENSBIXG00005016334	31.750632	2	1.27E-07	5.01E-06
ENSBIXG00005002526	37.5545439	4	1.38E-07	5.40E-06
ENSBIXG00005001709	31.5497472	2	1.41E-07	5.46E-06
ENSBIXG00005000636	34.6306503	3	1.46E-07	5.58E-06
ENSBIXG00005026079	75.8608426	23	1.47E-07	5.58E-06
ENSBIXG00005005083	44.8507286	7	1.46E-07	5.58E-06
ENSBIXG00005003353	42.4426904	6	1.50E-07	5.68E-06
ENSBIXG00005009420	37.2671359	4	1.59E-07	5.96E-06
ENSBIXG00005026717	27.3920877	1	1.66E-07	6.21E-06
ENSBIXG00005005951	41.9107945	6	1.91E-07	7.11E-06
ENSBIXG00005007938	52.8171888	11	1.94E-07	7.13E-06
ENSBIXG00005015994	27.0887083	1	1.94E-07	7.13E-06
ENSBIXG00005013872	33.875965	3	2.10E-07	7.68E-06
ENSBIXG00005007499	33.8611753	3	2.12E-07	7.69E-06
ENSBIXG00005017952	33.7866896	3	2.20E-07	7.92E-06
ENSBIXG00005022034	33.6386216	3	2.36E-07	8.46E-06
ENSBIXG00005022573	30.4394541	2	2.46E-07	8.75E-06
ENSBIXG00005000096	26.2480799	1	3.00E-07	1.06E-05
ENSBIXG00005029621	38.3492218	5	3.21E-07	1.13E-05
ENSBIXG00005019627	35.714128	4	3.31E-07	1.16E-05
ENSBIXG00005005345	40.6486795	6	3.40E-07	1.18E-05

ENSBIXG00005011842	62.8576666	17	3.52E-07	1.21E-05
ENSBIXG00005025569	49.3388663	10	3.53E-07	1.21E-05
ENSBIXG00005004905	40.5702882	6	3.52E-07	1.21E-05
ENSBIXG00005003045	38.1301591	5	3.55E-07	1.21E-05
ENSBIXG00005008711	32.7468669	3	3.64E-07	1.23E-05
ENSBIXG00005006038	45.0273124	8	3.64E-07	1.23E-05
ENSBIXG00005017177	57.1040788	14	3.75E-07	1.26E-05
ENSBIXG00005001346	37.9322758	5	3.89E-07	1.29E-05
ENSBIXG00005018396	40.3478688	6	3.89E-07	1.29E-05
ENSBIXG00005015768	35.1142054	4	4.40E-07	1.45E-05
ENSBIXG00005010883	34.5960342	4	5.62E-07	1.84E-05
ENSBIXG00005018494	46.1269391	9	5.70E-07	1.86E-05
ENSBIXG00005004135	43.8207446	8	6.15E-07	2.00E-05
ENSBIXG00005000933	63.1640299	18	6.24E-07	2.02E-05
ENSBIXG00005017614	24.7665696	1	6.47E-07	2.08E-05
ENSBIXG00005029405	24.7307886	1	6.59E-07	2.11E-05
ENSBIXG00005029285	24.6172401	1	6.99E-07	2.22E-05
ENSBIXG00005030396	38.5172408	6	8.90E-07	2.82E-05
PB.27545(chr29:10071831-10100345)	23.9059009	1	1.01E-06	3.18E-05
ENSBIXG00005022093	27.2391155	2	1.22E-06	3.81E-05
ENSBIXG00005028844	32.7974577	4	1.31E-06	4.09E-05
ENSBIXG00005019335	23.3152595	1	1.38E-06	4.26E-05
ENSBIXG00005009138	39.6950767	7	1.44E-06	4.44E-05
ENSBIXG00005008064	29.9006461	3	1.45E-06	4.45E-05
ENSBIXG00005010669	72.8002491	25	1.46E-06	4.47E-05

ENSBIXG00005003674	35.0041299	5	1.50E-06	4.57E-05
ENSBIXG00005007376	26.6832924	2	1.61E-06	4.86E-05
ENSBIXG00005009648	39.3821292	7	1.65E-06	4.97E-05
ENSBIXG00005022036	29.5991654	3	1.68E-06	5.00E-05
ENSBIXG00005029423	41.5142188	8	1.67E-06	5.00E-05
ENSBIXG00005014227	32.2521884	4	1.70E-06	5.04E-05
ENSBIXG00005012877	45.5055704	10	1.76E-06	5.20E-05
ENSBIXG00005015603	29.4760343	3	1.78E-06	5.23E-05
ENSBIXG00005016751	26.3987174	2	1.85E-06	5.42E-05
ENSBIXG00005012824	22.119404	1	2.56E-06	7.44E-05
ENSBIXG00005000501	33.8344711	5	2.57E-06	7.44E-05
ENSBIXG00005012192	21.9131016	1	2.85E-06	8.23E-05
ENSBIXG00005007396	25.4392173	2	2.99E-06	8.59E-05
ENSBIXG00005009658	30.9895228	4	3.08E-06	8.79E-05
ENSBIXG00005003088	37.9142098	7	3.15E-06	8.95E-05
ENSBIXG00005031139	35.6560146	6	3.22E-06	9.10E-05
ENSBIXG00005026495	37.8320094	7	3.26E-06	9.19E-05
ENSBIXG00005008869	28.1913492	3	3.31E-06	9.29E-05
ENSBIXG00005025367	60.3969579	19	3.35E-06	9.35E-05
ENSBIXG00005004685	27.9741323	3	3.68E-06	1.02E-04
ENSBIXG00005007473	30.2449825	4	4.36E-06	1.21E-04
ENSBIXG00005028073	24.5797821	2	4.60E-06	1.27E-04
ENSBIXG00005006845	59.4337159	19	4.75E-06	1.30E-04
ENSBIXG00005011725	27.3959944	3	4.86E-06	1.33E-04
ENSBIXG00005005344	32.2699861	5	5.25E-06	1.43E-04

ENSBIXG00005029055	34.5081026	6	5.37E-06	1.45E-04
ENSBIXG00005025885	20.6771924	1	5.44E-06	1.47E-04
ENSBIXG00005029176	46.3175953	12	6.12E-06	1.64E-04
ENSBIXG00005006348	23.9997478	2	6.14E-06	1.64E-04
ENSBIXG00005001135	34.1699969	6	6.24E-06	1.65E-04
ENSBIXG00005009895	23.9751132	2	6.22E-06	1.65E-04
ENSBIXG00005020290	66.9553567	24	6.26E-06	1.65E-04
ENSBIXG00005030433	20.2923004	1	6.65E-06	1.75E-04
ENSBIXG00005029641	68.3244092	25	6.80E-06	1.78E-04
ENSBIXG00005002998	19.9760196	1	7.84E-06	2.04E-04
ENSBIXG00005029790	41.8302368	10	8.04E-06	2.09E-04
ENSBIXG00005026480	31.3099317	5	8.14E-06	2.10E-04
ENSBIXG00005030714	19.8919284	1	8.19E-06	2.11E-04
ENSBIXG00005014615	41.7682331	10	8.25E-06	2.11E-04
ENSBIXG00005012083	28.7703671	4	8.70E-06	2.22E-04
ENSBIXG00005014137	19.769804	1	8.74E-06	2.22E-04
ENSBIXG00005026444	33.4011378	6	8.78E-06	2.22E-04
ENSBIXG00005025779	33.3908768	6	8.82E-06	2.22E-04
ENSBIXG00005001718	47.2190909	13	8.87E-06	2.23E-04
ENSBIXG00005029459	28.7203442	4	8.91E-06	2.23E-04
ENSBIXG00005017054	19.6523446	1	9.29E-06	2.31E-04
ENSBIXG00005014671	39.3790441	9	9.84E-06	2.44E-04
ENSBIXG00005029985	23.0291016	2	9.98E-06	2.47E-04
ENSBIXG00005024260	22.9704022	2	1.03E-05	2.53E-04
ENSBIXG00005011816	22.8729688	2	1.08E-05	2.65E-04

ENSBIXG00005025960	25.6993539	3	1.10E-05	2.69E-04
ENSBIXG00005016664	22.8099293	2	1.11E-05	2.71E-04
ENSBIXG00005017161	30.579365	5	1.13E-05	2.75E-04
ENSBIXG00005009350	42.775244	11	1.19E-05	2.87E-04
ENSBIXG00005013348	19.1133066	1	1.23E-05	2.96E-04
ENSBIXG00005029410	27.7678357	4	1.39E-05	3.33E-04
ENSBIXG00005018119	54.659632	18	1.45E-05	3.45E-04
ENSBIXG00005028672	61.2074249	22	1.48E-05	3.51E-04
ENSBIXG00005002448	27.4408069	4	1.62E-05	3.83E-04
ENSBIXG00005020523	36.1516009	8	1.65E-05	3.88E-04
ENSBIXG00005009534	29.3138522	5	2.01E-05	4.73E-04
ENSBIXG00005012385	26.9612204	4	2.02E-05	4.73E-04
ENSBIXG00005017176	31.5037708	6	2.03E-05	4.73E-04
PB.32103(chr5:6541748-6562966)	24.3987357	3	2.06E-05	4.79E-04
ENSBIXG00005029879	26.9095485	4	2.07E-05	4.80E-04
ENSBIXG00005001792	24.3302482	3	2.13E-05	4.91E-04
ENSBIXG00005025196	31.3745433	6	2.15E-05	4.94E-04
ENSBIXG00005009184	18.0086698	1	2.20E-05	5.03E-04
ENSBIXG00005007413	41.1486702	11	2.27E-05	5.18E-04
ENSBIXG00005027851	17.9372456	1	2.28E-05	5.19E-04
ENSBIXG00005017527	82.8460906	37	2.31E-05	5.20E-04
ENSBIXG00005020145	31.2218679	6	2.30E-05	5.20E-04
ENSBIXG00005001180	24.1118319	3	2.37E-05	5.30E-04
ENSBIXG00005000623	21.3093861	2	2.36E-05	5.30E-04
ENSBIXG00005021913	21.2883707	2	2.38E-05	5.32E-04

ENSBIXG00005031294	67.3261213	27	2.68E-05	5.95E-04
ENSBIXG00005015681	17.5184764	1	2.85E-05	6.28E-04
ENSBIXG00005026634	44.2052584	13	2.84E-05	6.28E-04
ENSBIXG00005014302	23.6468762	3	2.96E-05	6.51E-04
ENSBIXG00005025580	20.8240434	2	3.01E-05	6.59E-04
ENSBIXG00005012722	28.3430518	5	3.12E-05	6.81E-04
ENSBIXG00005002222	23.4901886	3	3.19E-05	6.94E-04
ENSBIXG00005003661	40.0473225	11	3.51E-05	7.61E-04
ENSBIXG00005012368	20.4423021	2	3.64E-05	7.86E-04
ENSBIXG00005002625	39.8482385	11	3.80E-05	8.14E-04
ENSBIXG00005001703	23.1344407	3	3.79E-05	8.14E-04
ENSBIXG00005007457	25.6023678	4	3.81E-05	8.14E-04
ENSBIXG00005003368	23.0455846	3	3.95E-05	8.42E-04
ENSBIXG00005020956	33.8513705	8	4.32E-05	9.18E-04
ENSBIXG00005015899	31.7347357	7	4.55E-05	9.53E-04
ENSBIXG00005016663	16.6332481	1	4.53E-05	9.53E-04
ENSBIXG00005020398	22.7527607	3	4.55E-05	9.53E-04
ENSBIXG00005028549	25.2355149	4	4.51E-05	9.53E-04
ENSBIXG00005008471	22.7162815	3	4.63E-05	9.66E-04
ENSBIXG00005001528	27.4566105	5	4.65E-05	9.67E-04
ENSBIXG00005003152	19.9244882	2	4.71E-05	9.78E-04
ENSBIXG00005021588	22.663272	3	4.75E-05	9.81E-04
ENSBIXG00005008543	29.55325	6	4.78E-05	9.85E-04
ENSBIXG00005014009	22.4952648	3	5.14E-05	0.00105636
ENSBIXG00005006510	37.1692286	10	5.29E-05	0.00108267

ENSBIXG00005030889	50.9439277	18	5.43E-05	0.00110707
ENSBIXG00005025335	16.2849681	1	5.45E-05	0.00110794
ENSBIXG00005028993	24.7998642	4	5.52E-05	0.00111846
ENSBIXG00005027913	24.7671193	4	5.60E-05	0.00113184
ENSBIXG00005026852	16.1917941	1	5.72E-05	0.00115245
ENSBIXG00005020914	61.8368806	25	5.81E-05	0.00116596
ENSBIXG00005010839	16.1184873	1	5.95E-05	0.0011902
ENSBIXG00005028612	26.8854498	5	6.00E-05	0.0011973
ENSBIXG00005016897	16.0706652	1	6.10E-05	0.00121281
ENSBIXG00005000390	28.8215853	6	6.58E-05	0.0012903
ENSBIXG00005005957	24.4375064	4	6.53E-05	0.0012903
ENSBIXG00005023486	36.62484	10	6.57E-05	0.0012903
ENSBIXG00005018018	19.2628782	2	6.56E-05	0.0012903
ENSBIXG00005006780	21.9009578	3	6.84E-05	0.00133802
ENSBIXG00005027411	38.3316484	11	6.88E-05	0.00134093
ENSBIXG00005019083	34.594424	9	7.02E-05	0.00136535
ENSBIXG00005006033	26.5009354	5	7.13E-05	0.00138229
ENSBIXG00005013672	30.5728525	7	7.45E-05	0.00143964
ENSBIXG00005022089	18.9897925	2	7.52E-05	0.00144886
ENSBIXG00005002843	32.4851822	8	7.62E-05	0.00146344
ENSBIXG00005029883	48.3106201	17	7.70E-05	0.00147307
ENSBIXG00005001254	26.2958816	5	7.82E-05	0.00149176
PB.24224(chr25:16412071-16419791)	18.8747157	2	7.97E-05	0.00151583
ENSBIXG00005030601	21.5701941	3	8.01E-05	0.00151972
ENSBIXG00005020510	21.5254346	3	8.19E-05	0.00153852

ENSBIXG00005005621	23.9474282	4	8.18E-05	0.00153852
ENSBIXG00005019953	28.3280951	6	8.15E-05	0.00153852
ENSBIXG00005015630	21.4072013	3	8.66E-05	0.00162317
ENSBIXG00005029916	30.2082586	7	8.70E-05	0.00162421
ENSBIXG00005016070	30.1987093	7	8.73E-05	0.00162588
ENSBIXG00005011435	15.2742649	1	9.30E-05	0.00172616
ENSBIXG00005003427	49.2179729	18	9.90E-05	0.00183284
ENSBIXG00005020801	21.1054829	3	1.00E-04	0.00184733
ENSBIXG00005021217	27.8111803	6	1.02E-04	0.00187642
ENSBIXG00005006700	37.2564245	11	1.04E-04	0.00191475
ENSBIXG00005031028	15.0261502	1	1.06E-04	0.00193954
ENSBIXG00005013152	31.5700711	8	1.11E-04	0.00202358
ENSBIXG00005026884	35.2928678	10	1.11E-04	0.00202358
ENSBIXG00005027649	18.1699642	2	1.13E-04	0.00205531
ENSBIXG00005008654	14.8428337	1	1.17E-04	0.00211252
ENSBIXG00005031030	27.4132831	6	1.21E-04	0.00218366
ENSBIXG00005014872	14.7468247	1	1.23E-04	0.00220359
ENSBIXG00005009043	14.7471589	1	1.23E-04	0.00220359
ENSBIXG00005008020	17.9493383	2	1.27E-04	0.00226195
ENSBIXG00005004540	22.9822231	4	1.28E-04	0.00227487
ENSBIXG00005025346	46.8223577	17	1.30E-04	0.00230457
ENSBIXG00005013369	14.6025866	1	1.33E-04	0.00234868
ENSBIXG00005030410	20.5117886	3	1.33E-04	0.00234868
ENSBIXG00005027990	17.8148766	2	0.00013538	0.00238489
ENSBIXG00005002513	14.554068	1	1.36E-04	0.00239247

ENSBIXG00005008424	27.1073381	6	1.38E-04	0.00242178
ENSBIXG00005019541	17.7521475	2	1.40E-04	0.00244008
ENSBIXG00005029077	51.3094739	20	1.43E-04	0.00249886
ENSBIXG00005024219	24.9030746	5	1.45E-04	0.00252674
ENSBIXG00005018568	28.9009128	7	1.51E-04	0.00261259
ENSBIXG00005023232	14.2124248	1	1.63E-04	0.00282048
ENSBIXG00005025372	14.1578192	1	1.68E-04	0.00289547
ENSBIXG00005030718	19.9842838	3	1.71E-04	0.00293766
ENSBIXG00005008505	32.3697574	9	1.72E-04	0.00294364
ENSBIXG00005017024	22.3274258	4	1.72E-04	0.00294634
ENSBIXG00005005934	17.2905185	2	1.76E-04	0.00299759
ENSBIXG00005004463	44.2893731	16	1.78E-04	0.00302799
ENSBIXG00005000556	26.4543208	6	1.83E-04	0.00309499
ENSBIXG00005006585	26.454591	6	1.83E-04	0.00309499
ENSBIXG00005008506	17.1920446	2	1.85E-04	0.00311465
ENSBIXG00005027106	13.9469897	1	1.88E-04	0.00315997
ENSBIXG00005011355	13.9394856	1	1.89E-04	0.00316148
ENSBIXG00005003469	24.3113854	5	1.89E-04	0.00316148
ENSBIXG00005011822	13.9128754	1	1.91E-04	0.00319189
ENSBIXG00005031377	13.8984449	1	1.93E-04	0.00320786
ENSBIXG00005002587	17.0219985	2	2.01E-04	0.00333665
ENSBIXG00005021186	13.8122401	1	2.02E-04	0.00334053
ENSBIXG00005012054	28.183875	7	2.04E-04	0.0033585
ENSBIXG00005018040	21.9593026	4	2.04E-04	0.00335863
ENSBIXG00005010670	21.7881861	4	2.21E-04	0.00361099

ENSBIXG00005008044	19.4493001	3	2.21E-04	0.00361099
ENSBIXG00005029268	21.783855	4	2.21E-04	0.00361099
ENSBIXG00005027791	40.3688426	14	2.23E-04	0.0036364
ENSBIXG00005005775	38.5652166	13	2.35E-04	0.00380692
ENSBIXG00005003150	13.5075195	1	2.38E-04	0.00384704
ENSBIXG00005002918	21.6185987	4	2.39E-04	0.0038505
ENSBIXG00005011264	36.8184077	12	2.39E-04	0.0038505
ENSBIXG00005002043	16.6703127	2	2.40E-04	0.00385445
ENSBIXG00005004673	13.4237436	1	2.48E-04	0.00398113
ENSBIXG00005030960	41.7038521	15	2.50E-04	0.0039888
ENSBIXG00005013675	27.6788558	7	2.51E-04	0.00400731
ENSBIXG00005013076	13.3868933	1	2.53E-04	0.00401675
ENSBIXG00005007709	27.6425294	7	2.55E-04	0.00401675
ENSBIXG00005008616	33.1892917	10	2.53E-04	0.00401675
ENSBIXG00005021901	19.1460636	3	2.55E-04	0.00401675
ENSBIXG00005009706	13.3813433	1	2.54E-04	0.00401675
ENSBIXG00005003214	23.5804214	5	2.61E-04	0.00410292
ENSBIXG00005017367	19.0892528	3	2.62E-04	0.00410305
ENSBIXG00005022971	31.1685597	9	2.77E-04	0.00432589
ENSBIXG00005012101	32.8971858	10	2.83E-04	0.00440498
ENSBIXG00005023101	21.2428735	4	2.83E-04	0.00440498
ENSBIXG00005018251	13.170489	1	2.84E-04	0.0044088
ENSBIXG00005024052	34.5975275	11	2.89E-04	0.00446365
ENSBIXG00005014737	21.1973596	4	2.89E-04	0.00446371
ENSBIXG00005011082	25.3603806	6	2.93E-04	0.00450203

ENSBIXG00005024072	37.9525959	13	2.93E-04	0.00450203
ENSBIXG00005028467	21.1369441	4	2.97E-04	0.00455464
ENSBIXG00005002652	16.2055301	2	3.03E-04	0.00462327
ENSBIXG00005028391	41.1429364	15	3.04E-04	0.00463374
ENSBIXG00005024670	29.0763058	8	3.07E-04	0.00467305
ENSBIXG00005010477	32.6624865	10	3.10E-04	0.00470504
ENSBIXG00005027389	12.8992259	1	3.29E-04	0.00497166
ENSBIXG00005017342	16.0214558	2	3.32E-04	0.00500732
ENSBIXG00005030705	28.8470352	8	3.37E-04	0.00507675
ENSBIXG00005009907	60.6127124	28	3.40E-04	0.0050791
ENSBIXG00005027601	12.839281	1	3.39E-04	0.0050791
ENSBIXG00005018867	28.8284994	8	3.40E-04	0.0050791
ENSBIXG00005028142	18.4969965	3	3.47E-04	0.00517732
ENSBIXG00005006190	15.909643	2	3.51E-04	0.00520738
ENSBIXG00005019320	50.1201095	21	3.51E-04	0.00520738
ENSBIXG00005009072	18.4601611	3	3.53E-04	0.00523107
ENSBIXG00005022240	15.889526	2	3.55E-04	0.00523415
ENSBIXG00005001920	15.8301537	2	3.65E-04	0.00537905
ENSBIXG00005004036	18.3821077	3	3.67E-04	0.00539023
ENSBIXG00005005509	12.614032	1	3.83E-04	0.00561259
ENSBIXG00005010752	20.4777876	4	4.02E-04	0.00586957
ENSBIXG00005018511	12.5215509	1	4.02E-04	0.00586957
ENSBIXG00005024769	12.5011389	1	4.07E-04	0.00592012
ENSBIXG00005005026	22.5559869	5	4.10E-04	0.00595898
ENSBIXG00005021238	15.4785152	2	4.35E-04	0.00629343

ENSBIXG00005030333	49.4499807	21	4.35E-04	0.00629343
ENSBIXG00005001653	24.378695	6	0.00044477	0.00641406
ENSBIXG00005027158	26.2658671	7	4.51E-04	0.00649498
ENSBIXG00005006544	26.244498	7	4.55E-04	0.00653728
ENSBIXG00005008165	15.3567326	2	4.63E-04	0.00662677
ENSBIXG00005029852	17.8687294	3	4.68E-04	0.00668875
ENSBIXG00005001982	17.7969692	3	4.84E-04	0.0069046
ENSBIXG00005020388	22.1689295	5	4.86E-04	0.00691584
ENSBIXG00005002265	24.0960856	6	5.01E-04	0.00711518
ENSBIXG00005026942	17.6677786	3	5.15E-04	0.00729086
ENSBIXG00005008955	29.5816575	9	5.17E-04	0.00729859
ENSBIXG00005027788	24.0016875	6	5.22E-04	0.00735502
ENSBIXG00005006331	15.1106594	2	5.23E-04	0.00735843
ENSBIXG00005000643	15.0118929	2	5.50E-04	0.00771344
ENSBIXG00005001284	19.7383932	4	5.62E-04	0.00785486
ENSBIXG00005016972	17.4836104	3	5.62E-04	0.00785486
ENSBIXG00005008145	27.5681624	8	5.64E-04	0.00785541
ENSBIXG00005017212	32.7636212	11	5.75E-04	0.00797135
ENSBIXG00005022755	32.7642659	11	5.74E-04	0.00797135
ENSBIXG00005009174	21.7734797	5	5.78E-04	0.00800227
ENSBIXG00005014801	11.811049	1	5.89E-04	0.00813177
ENSBIXG00005018463	17.3749519	3	5.92E-04	0.0081537
ENSBIXG00005009583	30.9564715	10	5.97E-04	0.00820176
ENSBIXG00005002955	21.6894284	5	6.00E-04	0.0082282
ENSBIXG00005020287	14.8236108	2	6.04E-04	0.00825087

ENSBIXG00005010528	14.8248187	2	6.04E-04	0.00825087
ENSBIXG00005005607	11.6913427	1	6.28E-04	0.00855759
ENSBIXG00005019856	14.7345449	2	6.32E-04	0.00858877
ENSBIXG00005000921	17.2302265	3	6.34E-04	0.00859888
ENSBIXG00005024446	25.4348664	7	6.35E-04	0.00859998
ENSBIXG00005025750	32.4623657	11	6.43E-04	0.00868278
ENSBIXG00005011718	32.4321221	11	6.50E-04	0.00876169
ENSBIXG00005007633	21.487624	5	6.55E-04	0.00881069
ENSBIXG00005006964	11.5973131	1	6.60E-04	0.00886166
ENSBIXG00005008880	23.4402976	6	6.62E-04	0.00886166
ENSBIXG00005011614	23.426142	6	6.66E-04	0.00889552
ENSBIXG00005007330	19.3274796	4	6.78E-04	0.00903669
ENSBIXG00005030114	17.0681045	3	6.84E-04	0.00910603
ENSBIXG00005000439	19.2872821	4	6.90E-04	0.00916337
ENSBIXG00005014939	23.311847	6	6.98E-04	0.00925493
ENSBIXG00005001627	14.5250902	2	7.01E-04	0.00925905
ENSBIXG00005009696	25.1912221	7	7.02E-04	0.00925905
ENSBIXG00005017706	14.4633256	2	7.23E-04	0.00952291
ENSBIXG00005022029	41.7572716	17	7.27E-04	0.00955069
ENSBIXG00005022661	14.4271317	2	7.37E-04	0.00965581
ENSBIXG00005016371	19.0918087	4	7.54E-04	0.00986356
ENSBIXG00005017742	14.3238452	2	7.76E-04	0.01012476
ENSBIXG00005008296	14.3174236	2	7.78E-04	0.01013598
ENSBIXG00005020138	11.2704216	1	7.88E-04	0.01023774
ENSBIXG00005005839	11.2169544	1	8.11E-04	0.0105149

ENSBIXG00005022063	28.3985185	9	8.18E-04	0.01056799
ENSBIXG00005023981	20.9807489	5	8.17E-04	0.01056799
ENSBIXG00005022819	18.8534027	4	8.40E-04	0.01082678
ENSBIXG00005022404	14.1599475	2	8.42E-04	0.01082982
ENSBIXG00005012220	14.1481761	2	8.47E-04	0.01087119
ENSBIXG00005024785	16.6075193	3	8.51E-04	0.01088058
ENSBIXG00005012059	16.6090679	3	8.50E-04	0.01088058
PB.36968(chr7:94802176-94814350)	11.078543	1	8.73E-04	0.01114297
ENSBIXG00005020884	14.0770271	2	8.77E-04	0.01117237
ENSBIXG00005025113	20.769552	5	8.95E-04	0.01137795
ENSBIXG00005001278	24.5484825	7	9.12E-04	0.01156573
ENSBIXG00005029748	22.658654	6	9.19E-04	0.01163316
ENSBIXG00005019400	10.9576365	1	9.32E-04	0.01177289
ENSBIXG00005004873	16.4099209	3	9.34E-04	0.01177623
ENSBIXG00005015465	10.9297837	1	9.46E-04	0.01190272
ENSBIXG00005023899	16.3754532	3	9.50E-04	0.0119212
ENSBIXG00005030316	13.8905905	2	9.63E-04	0.01206572
ENSBIXG00005016033	10.8530272	1	9.86E-04	0.01233129
ENSBIXG00005031284	24.3124987	7	0.00100382	0.01252449
ENSBIXG00005002616	18.4470031	4	0.00100898	0.0125591
ENSBIXG00005031540	24.2958009	7	0.00101064	0.0125591
ENSBIXG00005022864	10.7346353	1	0.00105149	0.01304055
ENSBIXG00005002987	20.3913714	5	0.00105505	0.01305034
ENSBIXG00005026618	22.3264636	6	0.00105649	0.01305034
ENSBIXG00005021196	10.7037088	1	0.00106921	0.0131488

ENSBIXG00005004883	10.7009283	1	0.00107082	0.0131488
ENSBIXG00005001801	42.1021853	18	0.00107012	0.0131488
ENSBIXG00005031184	10.6867178	1	0.00107908	0.013224
ENSBIXG00005025092	10.6775404	1	0.00108444	0.01326356
ENSBIXG00005005361	13.6408347	2	0.00109127	0.01332074
ENSBIXG00005020351	18.1966835	4	0.00112951	0.01376051
ENSBIXG00005025283	20.2214655	5	0.0011356	0.01380755
ENSBIXG00005014948	15.9923266	3	0.0011381	0.01381087
ENSBIXG00005011714	13.5281657	2	0.00115451	0.01392819
ENSBIXG00005026144	18.1481352	4	0.00115448	0.01392819
ENSBIXG00005007118	15.9658785	3	0.0011524	0.01392819
ENSBIXG00005004176	23.9506505	7	0.00116233	0.01399537
ENSBIXG00005005579	10.4965802	1	0.00119596	0.01437234
ENSBIXG00005006759	10.4826433	1	0.00120501	0.01445316
ENSBIXG00005001739	18.0144746	4	0.00122609	0.01467752
ENSBIXG00005030187	19.9834386	5	0.00125871	0.01503898
ENSBIXG00005030135	13.3178171	2	0.00128255	0.01529435
ENSBIXG00005016186	15.728868	3	0.00128875	0.01533885
ENSBIXG00005013295	17.8915662	4	0.00129579	0.0153931
ENSBIXG00005015834	15.7051376	3	0.00130325	0.01545216
ENSBIXG00005013047	17.8348774	4	0.00132925	0.01573025
ENSBIXG00005013365	17.8196693	4	0.00133836	0.015808
ENSBIXG00005013052	10.2806123	1	0.00134435	0.01584855
ENSBIXG00005022052	25.3451419	8	0.00135838	0.01598348
ENSBIXG00005020002	17.6513813	4	0.00144349	0.01695283

ENSBIXG00005013497	17.6295358	4	0.00145772	0.0170876
ENSBIXG00005013481	13.0496302	2	0.00146659	0.01712679
ENSBIXG00005024029	17.6171335	4	0.00146586	0.01712679
ENSBIXG00005027514	12.9717676	2	0.00152481	0.01777324
ENSBIXG00005026202	10.0202332	1	0.0015483	0.01800985
ENSBIXG00005015906	10.0171168	1	0.00155092	0.01800985
ENSBIXG00005026294	17.4525016	4	0.00157828	0.01829325
ENSBIXG00005020120	19.4125092	5	0.00161001	0.01862621
ENSBIXG00005020155	9.93593793	1	0.00162083	0.0186817
ENSBIXG00005019129	17.3965607	4	0.00161838	0.0186817
ENSBIXG00005009734	12.8303713	2	0.00163652	0.01876365
ENSBIXG00005008677	17.371023	4	0.00163702	0.01876365
ENSBIXG00005026115	21.2753031	6	0.0016369	0.01876365
ENSBIXG00005030190	17.3508478	4	0.00165189	0.01889921
ENSBIXG00005031188	15.1973308	3	0.00165555	0.0189062
ENSBIXG00005028205	9.89345552	1	0.00165868	0.01890708
ENSBIXG00005008700	60.5201514	32	0.00169711	0.01930974
ENSBIXG00005012389	9.831011	1	0.00171594	0.01948818
ENSBIXG00005003530	12.7298125	2	0.0017209	0.01950881
ENSBIXG00005006198	29.7321358	11	0.00174548	0.0197513
ENSBIXG00005025904	26.3907985	9	0.00176273	0.01991019
ENSBIXG00005008435	22.9109262	7	0.00176657	0.01991722
ENSBIXG00005021153	9.77206877	1	0.00177183	0.01994032
ENSBIXG00005010531	17.1831694	4	0.00178079	0.02000485
ENSBIXG00005007105	28.0160969	10	0.00179457	0.02008684

ENSBIXG00005006978	31.2660466	12	0.00179406	0.02008684
ENSBIXG00005012153	15.022127	3	0.00179784	0.02008719
ENSBIXG00005011825	26.3135882	9	0.00181497	0.02024209
ENSBIXG00005001335	14.9966853	3	0.00181948	0.02025605
ENSBIXG00005010796	34.3528666	14	0.00183105	0.02034823
ENSBIXG00005009546	14.9598381	3	0.00185129	0.02053644
ENSBIXG00005019251	19.0497387	5	0.00188156	0.02083492
ENSBIXG00005010835	26.1978434	9	0.0018961	0.02095845
PB.11603(chr16:55669671-55693267)	9.63476832	1	0.00190928	0.02106666
ENSBIXG00005022202	26.1563842	9	0.001926	0.02121341
ENSBIXG00005024974	20.873765	6	0.00193291	0.02125177
ENSBIXG00005021407	24.3985895	8	0.00196427	0.02155826
ENSBIXG00005006651	20.7969386	6	0.00199524	0.02185951
ENSBIXG00005021503	9.54975125	1	0.00199977	0.02187045
ENSBIXG00005023641	9.52964716	1	0.00202179	0.02207239
ENSBIXG00005029121	18.8700986	5	0.00203221	0.02214711
ENSBIXG00005028058	18.8377914	5	0.00206053	0.02241636
ENSBIXG00005014624	16.8177671	4	0.00209704	0.02277362
ENSBIXG00005025925	14.6807396	3	0.00211084	0.02287698
ENSBIXG00005019322	22.4618593	7	0.00211394	0.02287698
ENSBIXG00005005783	14.6314637	3	0.00216027	0.02333774
ENSBIXG00005021603	45.6734762	22	0.00217787	0.02348687
ENSBIXG00005000424	24.1089413	8	0.00219735	0.02353324
ENSBIXG00005016706	24.1093126	8	0.00219703	0.02353324
ENSBIXG00005025754	18.6879511	5	0.00219703	0.02353324

ENSBIXG00005016325	18.6888932	5	0.00219614	0.02353324
ENSBIXG00005005580	9.36500572	1	0.00221167	0.02364584
PB.6810(chr12:86079603-86113432)	20.5382858	6	0.00221986	0.02369254
ENSBIXG00005004322	16.658772	4	0.00225137	0.02398752
ENSBIXG00005001065	14.5263399	3	0.00226961	0.02414036
ENSBIXG00005008637	9.29953995	1	0.00229211	0.02433801
ENSBIXG00005021763	12.1467964	2	0.00230333	0.02441533
ENSBIXG00005025871	9.28573497	1	0.00230945	0.02443842
ENSBIXG00005008674	23.9355214	8	0.00234954	0.02482021
ENSBIXG00005019091	12.0530138	2	0.00241391	0.02545691
ENSBIXG00005020946	9.1893253	1	0.00243431	0.02562841
ENSBIXG00005011087	16.4748091	4	0.00244394	0.02568623
ENSBIXG00005002110	16.4600952	4	0.00246003	0.02581154
ENSBIXG00005010070	22.0760665	7	0.00246483	0.0258182
ENSBIXG00005028991	11.9925466	2	0.00248801	0.02601709
ENSBIXG00005001356	11.9829039	2	0.00250003	0.02609881
ENSBIXG00005027348	11.9458254	2	0.00254681	0.02654249
ENSBIXG00005003200	14.2538397	3	0.00257923	0.0268352
ENSBIXG00005023644	20.15359	6	0.00260032	0.02700937
ENSBIXG00005016418	18.2716849	5	0.00262449	0.02721483
ENSBIXG00005006999	18.2548899	5	0.00264335	0.02736463
ENSBIXG00005019505	9.02927462	1	0.0026569	0.02745905
ENSBIXG00005005902	11.8467056	2	0.00267621	0.02755827
ENSBIXG00005002182	18.2277359	5	0.00267412	0.02755827
ENSBIXG00005006680	9.01356529	1	0.00267983	0.02755827

ENSBIXG00005015121	9.0030754	1	0.00269526	0.02767101
ENSBIXG00005000466	11.8167086	2	0.00271665	0.02784458
ENSBIXG00005020507	16.2249718	4	0.00273168	0.02795234
ENSBIXG00005004592	8.97443457	1	0.00273783	0.0279692
ENSBIXG00005013519	19.98038	6	0.00279175	0.02842636
ENSBIXG00005021510	8.94104158	1	0.00278834	0.02842636
ENSBIXG00005011947	11.7360975	2	0.00282839	0.02871597
ENSBIXG00005023072	8.91430435	1	0.00282946	0.02871597
ENSBIXG00005001466	26.7551346	10	0.00284693	0.02884605
ENSBIXG00005009931	18.0758162	5	0.00285287	0.02885914
ENSBIXG00005002433	18.0697304	5	0.00286027	0.02888687
ENSBIXG00005028591	14.0073483	3	0.00289517	0.02919176
ENSBIXG00005025373	17.9770354	5	0.00297532	0.02995127
ENSBIXG00005006402	17.9581494	5	0.00299932	0.03013929
ENSBIXG00005007369	16.0078989	4	0.00300858	0.03013929
ENSBIXG00005030244	8.80515584	1	0.00300381	0.03013929
ENSBIXG00005014625	11.5618056	2	0.00308593	0.03086426
ENSBIXG00005014960	21.4971248	7	0.0031	0.03090533
ENSBIXG00005000161	11.5549499	2	0.00309652	0.03090533
ENSBIXG00005015367	15.9322614	4	0.00311142	0.03096935
ENSBIXG00005012952	17.8527063	5	0.00313678	0.03107202
ENSBIXG00005011796	37.2244957	17	0.00313718	0.03107202
ENSBIXG00005011549	15.8996555	4	0.00315681	0.03107202
ENSBIXG00005016571	8.71991482	1	0.00314753	0.03107202
ENSBIXG00005018536	26.4858967	10	0.00313882	0.03107202

ENSBIXG00005005890	17.8393278	5	0.00315466	0.03107202
ENSBIXG00005000704	13.8295367	3	0.00314661	0.03107202
ENSBIXG00005021493	11.4887573	2	0.00320072	0.03139705
ENSBIXG00005026505	24.7962572	9	0.00320502	0.03139705
ENSBIXG00005026615	28.0341441	11	0.00319859	0.03139705
ENSBIXG00005005918	17.7674932	5	0.00325237	0.03181063
ENSBIXG00005008273	15.8012175	4	0.00329784	0.03220453
ENSBIXG00005021696	8.63105447	1	0.0033048	0.03222182
ENSBIXG00005022780	17.7078526	5	0.00333574	0.0324523
ENSBIXG00005004055	11.4042228	2	0.00333891	0.0324523
ENSBIXG00005010114	41.3585453	20	0.00334957	0.03250502
ENSBIXG00005007916	15.7579353	4	0.00336179	0.03257257
ENSBIXG00005024878	19.5053806	6	0.00339004	0.03279506
ENSBIXG00005012728	13.6667753	3	0.00339567	0.03279839
PB.38092(chr8:59087253-59114232)	13.6490653	3	0.00342392	0.03301983
ENSBIXG00005010415	8.5497943	1	0.00345561	0.03327369
ENSBIXG00005016181	11.3318935	2	0.00346187	0.03328225
ENSBIXG00005026519	15.6691675	4	0.00349679	0.03351406
ENSBIXG00005007599	8.53041667	1	0.0034926	0.03351406
ENSBIXG00005019559	22.8597255	8	0.00354909	0.03389018
ENSBIXG00005026248	21.1588632	7	0.003542	0.03389018
ENSBIXG00005019988	13.5702942	3	0.00355243	0.03389018
ENSBIXG00005005046	13.5367904	3	0.00360852	0.03437244
ENSBIXG00005025085	8.46586114	1	0.00361874	0.03441687
ENSBIXG00005011081	8.45182891	1	0.00364677	0.03463031

ENSBIXG00005006403	21.0400725	7	0.00371129	0.03516594
ENSBIXG00005029180	11.1910159	2	0.00371451	0.03516594
ENSBIXG00005021537	22.7202592	8	0.00374259	0.03537778
ENSBIXG00005008079	24.3097789	9	0.00383751	0.03621979
ENSBIXG00005006792	27.5024869	11	0.00385598	0.03633877
ENSBIXG00005027120	24.2760585	9	0.00388556	0.03656196
ENSBIXG00005027502	8.32298782	1	0.00391465	0.0367799
ENSBIXG00005006809	15.3828355	4	0.00396963	0.03724008
ENSBIXG00005022938	13.304931	3	0.0040215	0.03766969
ENSBIXG00005019962	24.1453669	9	0.00407731	0.03813483
ENSBIXG00005026732	13.2560196	3	0.00411442	0.03833204
ENSBIXG00005001719	17.2107675	5	0.00411694	0.03833204
ENSBIXG00005030361	10.9856264	2	0.00411625	0.03833204
ENSBIXG00005014922	24.105223	9	0.00413802	0.03841296
ENSBIXG00005006542	10.9768788	2	0.00413429	0.03841296
ENSBIXG00005018014	22.4504133	8	0.00414638	0.03843303
ENSBIXG00005012135	8.20751983	1	0.00417171	0.03860925
ENSBIXG00005014765	13.2232615	3	0.00417784	0.03860925
ENSBIXG00005009457	17.166893	5	0.00419392	0.03870013
ENSBIXG00005015711	18.9763294	6	0.0042038	0.03873364
ENSBIXG00005013552	15.2190213	4	0.00426786	0.03920739
ENSBIXG00005013120	13.1796181	3	0.00426384	0.03920739
ENSBIXG00005018965	22.3586541	8	0.00429304	0.03938042
ENSBIXG00005020926	8.1310921	1	0.00435125	0.03985542
ENSBIXG00005013019	10.866786	2	0.00436825	0.03995208

ENSBIXG00005026787	17.0662947	5	0.00437577	0.03996191
ENSBIXG00005000976	20.5993722	7	0.00441064	0.04017281
ENSBIXG00005028873	15.1439307	4	0.00441182	0.04017281
ENSBIXG00005022102	13.1008012	3	0.00442361	0.04022113
ENSBIXG00005013049	25.5042953	10	0.00446731	0.040559
ENSBIXG00005028315	13.0755261	3	0.00447609	0.04057929
ENSBIXG00005021627	8.07288145	1	0.00449326	0.04067549
ENSBIXG00005029750	10.8074225	2	0.00449985	0.04067576
ENSBIXG00005018810	13.0362969	3	0.00455877	0.04114833
ENSBIXG00005000502	8.02156875	1	0.00462235	0.04166163
ENSBIXG00005026588	16.9250577	5	0.00464414	0.04179728
ENSBIXG00005009728	10.7239252	2	0.00469169	0.04216401
ENSBIXG00005024843	18.6601192	6	0.00477786	0.04287626
PB.38288(chr8:69397067-69465250)	7.95649711	1	0.00479151	0.0429366
ENSBIXG00005015056	10.6579565	2	0.00484902	0.0433893
ENSBIXG00005029979	10.6524597	2	0.00486237	0.04344602
ENSBIXG00005025308	7.91913016	1	0.00489148	0.04364325
ENSBIXG00005009081	12.8638716	3	0.00494043	0.0440167
PB.28146(chr3:10536308-10623429)	16.7641183	5	0.00496958	0.04417917
ENSBIXG00005007885	10.6074971	2	0.00497292	0.04417917
ENSBIXG00005000750	18.518953	6	0.0050581	0.04487164
ENSBIXG00005021374	20.2423474	7	0.00506933	0.04490702
ENSBIXG00005019414	23.5465917	9	0.00507873	0.04492615
ENSBIXG00005005393	18.5049732	6	0.00508671	0.04493258
ENSBIXG00005020588	12.7950939	3	0.00510135	0.0449978

ENSBIXG00005025612	25.1093636	10	0.00514202	0.04529213
ENSBIXG00005005253	10.5333146	2	0.00516083	0.04539337
ENSBIXG00005008687	7.81203909	1	0.00518993	0.04558465
ENSBIXG00005029838	16.641488	5	0.00523239	0.04589257
ENSBIXG00005002667	20.150326	7	0.00525397	0.04601678
ENSBIXG00005018990	14.7135466	4	0.00533375	0.04664962
ENSBIXG00005031536	10.4448059	2	0.00539435	0.0471132
ENSBIXG00005024933	16.5574009	5	0.00542039	0.04727402
ENSBIXG00005024741	12.6547377	3	0.00544599	0.04743055
ENSBIXG00005011417	7.71661653	1	0.00547148	0.0475858
ENSBIXG00005020724	24.9028902	10	0.00553265	0.04803278
ENSBIXG00005014254	10.3921099	2	0.00553837	0.04803278
ENSBIXG00005017356	7.65772808	1	0.00565297	0.04895815
ENSBIXG00005030022	14.5542129	4	0.00572099	0.04939537
ENSBIXG00005002063	7.63421051	1	0.00572715	0.04939537
ENSBIXG00005027512	16.4260386	5	0.00572735	0.04939537
ENSBIXG00005014736	12.5317032	3	0.00576697	0.04966799
ENSBIXG00005007786	19.8939166	7	0.00580344	0.04991277

Table S8. The significant DTUs ($p < 0.05$) identified using Iso-Seq technology.

Gene ID	likelihood ratio statistics	degrees of freedom	p-value	adjust p-value
ENSBIXG00005004010	1008.02667	18	3.27E-188	3.60E-184
ENSBIXG00005007542	409.67934	18	6.60E-65	3.63E-61

ENSBIXG00005011852	267.424436	18	1.28E-44	4.68E-41
PB.16479(chr19:63575740-63609965)	292.52201	18	2.36E-43	6.48E-40
ENSBIXG00005022063	237.822234	18	1.30E-35	2.85E-32
PB.19319(chr21:1973995-1979927)	228.478303	18	1.58E-31	2.89E-28
ENSBIXG00005029889	150.421338	18	1.60E-28	2.52E-25
ENSBIXG00005000548	190.283597	18	4.66E-26	6.40E-23
ENSBIXG00005016992	174.805764	18	8.89E-26	1.09E-22
ENSBIXG00005015502	134.301979	18	6.18E-24	6.80E-21
PB.20892(chr22:12720297-12728339)	172.697548	18	2.23E-22	2.23E-19
ENSBIXG00005003661	171.747104	18	2.00E-21	1.83E-18
ENSBIXG00005011180	138.452702	18	4.36E-21	3.69E-18
ENSBIXG00005005332	148.809919	18	8.02E-21	6.30E-18
ENSBIXG00005008345	150.254518	18	1.89E-19	1.39E-16
PB.16032(chr19:49267630-49357766)	119.49584	18	1.92E-17	1.32E-14
PB.16145(chr19:51943946-51958822)	83.2227228	18	1.77E-16	1.15E-13
ENSBIXG00005019381	85.5972806	18	1.24E-14	7.58E-12
PB.29663(chr3:99563603-99633697)	138.367662	18	6.35E-14	3.68E-11
PB.9513(chr15:1743877-1891946)	100.725732	18	9.34E-13	5.14E-10

PB.34736(chr6:58764940-58831483)	128.921695	18	1.13E-12	5.89E-10
ENSBIXG00005010835	103.87974	18	2.99E-12	1.49E-09
ENSBIXG00005017232	86.3894659	18	4.65E-12	2.23E-09
ENSBIXG00005007376	71.0190936	18	9.61E-12	4.40E-09
PB.25344(chr26:32691762-32697739)	92.9296232	18	1.00E-11	4.40E-09
ENSBIXG00005023373	92.5452732	18	1.17E-11	4.95E-09
ENSBIXG00005017376	58.8634326	18	2.09E-11	8.50E-09
ENSBIXG00005008286	81.5478551	18	8.74E-11	3.43E-08
ENSBIXG00005011434	112.639436	18	1.25E-10	4.74E-08
ENSBIXG00005020385	90.1449429	21	1.52E-10	5.59E-08
ENSBIXG00005007178	74.4471315	18	2.99E-10	1.06E-07
PB.32119(chr5:6971631-6989332)	99.6882657	18	5.69E-10	1.96E-07
ENSBIXG00005008643	73.8920342	18	8.97E-10	2.99E-07
ENSBIXG00005031540	75.4101701	18	1.11E-09	3.58E-07
PB.23722(chr25:4113454-4125673)	66.291724	18	1.57E-09	4.94E-07
ENSBIXG00005027278	41.539928	18	5.02E-09	1.53E-06
ENSBIXG00005014694	41.4223218	18	5.32E-09	1.54E-06
PB.34727(chr6:58698918-58751422)	75.4650647	18	5.26E-09	1.54E-06
ENSBIXG00005010659	61.0397674	18	5.94E-09	1.68E-06

ENSBIXG00005002395	42.0682118	18	1.61E-08	4.44E-06
PB.36968(chr7:94802176-94814350)	66.3512779	18	1.98E-08	5.31E-06
ENSBIXG00005010669	110.619341	18	2.59E-08	6.79E-06
ENSBIXG00005024769	43.6071407	18	2.78E-08	7.12E-06
ENSBIXG00005031233	40.0524917	4	4.22E-08	1.06E-05
PB.37807(chr8:38483047-38759945)	102.040729	18	5.35E-08	1.31E-05
ENSBIXG00005031552	74.0678716	18	7.72E-08	1.85E-05
ENSBIXG00005016694	115.876857	18	9.58E-08	2.24E-05
ENSBIXG00005009001	58.4760484	18	9.82E-08	2.25E-05
ENSBIXG00005006456	42.8748296	18	1.23E-07	2.77E-05
PB.11885(chr16:75203717-75371535)	111.748188	45	1.34E-07	2.94E-05
ENSBIXG00005012018	80.890975	18	1.53E-07	3.29E-05
ENSBIXG00005024324	55.2375965	18	1.64E-07	3.47E-05
ENSBIXG00005003152	63.6756722	18	2.57E-07	5.33E-05
ENSBIXG00005026849	66.7776872	18	3.13E-07	6.38E-05
ENSBIXG00005012171	66.4944566	18	3.48E-07	6.97E-05
ENSBIXG00005003223	84.3940368	18	4.53E-07	8.79E-05
ENSBIXG00005005877	25.443586	1	4.56E-07	8.79E-05
ENSBIXG00005031107	79.0846253	18	5.19E-07	9.85E-05
ENSBIXG00005018229	65.2863294	18	5.48E-07	1.02E-04

ENSBIXG00005023156	50.2290496	18	5.69E-07	1.04E-04
PB.40969(chrX:13224913-13318080)	112.442042	18	6.80E-07	1.23E-04
PB.2841(chr10:40851802-40868644)	59.1574682	18	7.25E-07	1.29E-04
ENSBIXG00005005229	40.9958977	18	8.11E-07	1.42E-04
ENSBIXG00005025242	33.675203	18	8.69E-07	1.49E-04
ENSBIXG00005015894	30.8962683	3	8.94E-07	1.51E-04
ENSBIXG00005014257	27.5855962	18	1.02E-06	1.70E-04
ENSBIXG00005027604	37.9909497	6	1.13E-06	1.85E-04
ENSBIXG00005021883	55.942942	18	1.24E-06	2.00E-04
ENSBIXG00005016748	64.6286566	18	1.34E-06	2.13E-04
ENSBIXG00005006900	72.219664	18	1.79E-06	2.82E-04
ENSBIXG00005005553	29.216394	18	2.02E-06	3.12E-04
PB.24123(chr25:14785042-14811863)	46.9956088	18	2.15E-06	3.29E-04
ENSBIXG00005026004	34.0947272	18	2.28E-06	3.44E-04
ENSBIXG00005030486	33.9941811	18	2.39E-06	3.55E-04
PB.21494(chr22:43939399-43951434)	52.1208132	18	2.68E-06	3.93E-04
PB.12319(chr17:8691917-8709902)	42.0294071	9	3.25E-06	4.64E-04
PB.16171(chr19:52193574-52212381)	58.7529568	18	3.25E-06	4.64E-04

ENSBIXG00005024440	32.7778524	5	4.17E-06	5.87E-04
ENSBIXG00005030139	88.2951827	18	4.43E-06	6.18E-04
ENSBIXG00005005507	45.1429952	18	4.58E-06	6.30E-04
ENSBIXG00005020956	24.5311279	2	4.71E-06	6.40E-04
ENSBIXG00005014919	27.2116927	18	5.32E-06	7.13E-04
PB.20814(chr22:10727777-10775442)	68.3644775	18	6.71E-06	8.90E-04
ENSBIXG00005009277	47.8596008	18	6.91E-06	9.05E-04
PB.2687(chr10:32310810-32354720)	58.3106736	18	7.14E-06	9.24E-04
ENSBIXG00005014432	58.2403975	18	7.32E-06	9.36E-04
ENSBIXG00005017614	26.4203402	18	7.79E-06	9.85E-04
ENSBIXG00005028060	51.1194737	18	7.89E-06	9.86E-04
ENSBIXG00005026175	25.9467356	18	9.79E-06	0.00120944
ENSBIXG00005020530	60.6831508	18	1.01E-05	0.0012296
ENSBIXG00005014567	44.7292696	18	1.15E-05	0.00138595
ENSBIXG00005015705	59.7284372	18	1.40E-05	0.00167848
PB.33649(chr5:93218040-93219853)	36.4557458	18	1.45E-05	0.00171364
ENSBIXG00005000426	21.1519009	18	2.55E-05	0.00298668
ENSBIXG00005025146	21.0425157	2	2.70E-05	0.00312137
ENSBIXG00005008760	36.7220246	18	2.95E-05	0.00334537
PB.16325(chr19:56911635-56912408)	40.5035062	18	2.93E-05	0.00334537

PB.24757(chr25:41147235-41172326)	48.5985575	18	3.82E-05	0.00429187
ENSBIXG00005008802	20.2907322	2	3.93E-05	0.00436195
ENSBIXG00005015576	35.985245	18	3.99E-05	0.00438749
ENSBIXG00005005187	43.2790893	18	4.04E-05	0.00439788
ENSBIXG00005025598	22.7645204	18	4.52E-05	0.004876
ENSBIXG00005004863	46.0789763	18	5.17E-05	0.00551646
ENSBIXG00005002643	35.1064925	18	5.71E-05	0.00597776
PB.36408(chr7:69390189-69392416)	44.1204235	18	5.66E-05	0.00597776
ENSBIXG00005018882	16.0930334	1	6.03E-05	0.00625812
PB.18945(chr20:25789814-25818510)	58.126061	18	7.10E-05	0.00729449
ENSBIXG00005001116	46.7202631	18	7.53E-05	0.00766781
PB.3630(chr10:80143893-80194985)	60.8748672	18	7.91E-05	0.00798521
ENSBIXG00005014086	46.225835	18	8.98E-05	0.00898343
ENSBIXG00005009383	31.9952602	18	9.33E-05	0.00924831
ENSBIXG00005018018	35.68008	18	9.55E-05	0.00938168
ENSBIXG00005003544	29.7774825	18	1.04E-04	0.01015412
ENSBIXG00005008273	50.5769064	18	1.08E-04	0.01039556
ENSBIXG00005000599	23.2595286	18	1.12E-04	0.01074849
ENSBIXG00005019414	71.4420713	18	1.19E-04	0.01126509
ENSBIXG00005024645	23.1168534	18	1.20E-04	0.01128187

ENSBIXG00005013045	40.2698782	18	1.25E-04	0.01165859
ENSBIXG00005012981	31.1129993	18	1.34E-04	0.0123998
ENSBIXG00005029373	41.7389662	18	1.36E-04	0.01246517
ENSBIXG00005011513	28.9550263	18	1.47E-04	0.01340406
ENSBIXG00005000232	49.4822245	18	1.56E-04	0.01398091
ENSBIXG00005014406	26.8223919	18	1.56E-04	0.01398091
ENSBIXG00005001102	55.5855766	18	1.62E-04	0.01411557
ENSBIXG00005025472	30.6762445	18	1.60E-04	0.01411557
ENSBIXG00005030960	60.1343558	18	1.61E-04	0.01411557
ENSBIXG00005023458	22.449049	18	1.63E-04	0.01412771
ENSBIXG00005023035	14.1140437	1	1.72E-04	0.01478593
ENSBIXG00005000394	41.0323212	18	1.76E-04	0.01499089
ENSBIXG00005016209	17.2547632	18	1.79E-04	0.01515741
ENSBIXG00005013327	81.1386989	18	1.89E-04	0.01574523
PB.1469(chr1:115444316-115534516)	52.041294	18	1.88E-04	0.01574523
ENSBIXG00005005761	40.8052908	18	1.91E-04	0.01578549
ENSBIXG00005018761	22.0656678	4	1.94E-04	0.01596456
ENSBIXG00005006693	31.9937302	18	2.00E-04	0.01626583
ENSBIXG00005020914	62.295628	18	2.06E-04	0.01663261
ENSBIXG00005019708	43.8118097	18	2.11E-04	0.01691272
ENSBIXG00005029164	29.9907275	18	2.12E-04	0.01691272
ENSBIXG00005028479	43.7534453	18	2.15E-04	0.01701487

ENSBIXG00005009015	35.1189577	18	2.37E-04	0.01860965
ENSBIXG00005031446	33.3051326	18	2.42E-04	0.01888067
ENSBIXG00005026666	34.7425374	11	2.73E-04	0.0211654
ENSBIXG00005004648	31.1632835	18	2.78E-04	0.0213485
ENSBIXG00005014651	58.3268542	18	2.80E-04	0.02141207
ENSBIXG00005009706	27.3764086	18	2.85E-04	0.02148167
ENSBIXG00005010240	34.6120067	18	2.87E-04	0.02148167
ENSBIXG00005017850	34.6232201	18	2.86E-04	0.02148167
ENSBIXG00005006504	56.6649453	18	2.96E-04	0.02202603
ENSBIXG00005026082	41.0807102	18	3.11E-04	0.02294915
ENSBIXG00005000134	23.1427208	18	3.17E-04	0.02324694
ENSBIXG00005019344	62.2121806	18	3.25E-04	0.02368861
ENSBIXG00005022121	45.5235297	18	3.48E-04	0.02518674
ENSBIXG00005031281	47.0571371	18	3.51E-04	0.02520007
ENSBIXG00005029451	30.5298869	18	3.56E-04	0.0254509
ENSBIXG00005029121	12.715298	1	3.63E-04	0.02573833
ENSBIXG00005005056	40.5511924	18	3.74E-04	0.02630093
ENSBIXG00005030622	18.3335699	18	3.75E-04	0.02630093
ENSBIXG00005030508	15.6767945	2	3.94E-04	0.0274513
ENSBIXG00005003860	12.5223502	1	4.02E-04	0.02764525
ENSBIXG00005011271	20.4792029	18	4.02E-04	0.02764525
ENSBIXG00005007421	12.4934357	1	4.08E-04	0.02772981

ENSBIXG00005008995	37.0534552	18	4.06E-04	0.02772981
ENSBIXG00005014287	33.6343759	18	4.15E-04	0.02800479
ENSBIXG00005013475	22.4872861	18	4.23E-04	0.02836518
ENSBIXG00005000434	33.5096925	11	4.35E-04	0.02898985
ENSBIXG00005000161	44.8286174	18	4.39E-04	0.02907026
ENSBIXG00005014897	22.261916	18	4.67E-04	0.03045043
ENSBIXG00005030889	52.2151349	18	4.68E-04	0.03045043
PB.32271(chr5:10908380-10949113)	39.9184114	18	4.67E-04	0.03045043
PB.10035(chr15:36140829-36212600)	60.7155112	29	5.03E-04	0.03253224
ENSBIXG00005005030	31.05964	18	5.74E-04	0.03689601
ENSBIXG00005029325	48.4753332	18	5.92E-04	0.03787421
ENSBIXG00005020343	17.3507626	18	5.99E-04	0.03804325
ENSBIXG00005023567	25.5667126	18	6.02E-04	0.03804325
ENSBIXG00005025215	43.8371828	18	6.09E-04	0.03825623
PB.32124(chr5:7034118-7159232)	40.628595	18	6.30E-04	0.03936395
ENSBIXG00005027356	35.6282023	18	6.77E-04	0.04210417
PB.10777(chr16:1956937-2018352)	40.3724925	16	6.87E-04	0.04244118
ENSBIXG00005003180	19.2482252	18	7.02E-04	0.0429327
ENSBIXG00005009506	21.3270286	18	7.03E-04	0.0429327
ENSBIXG00005020105	28.5649195	18	7.67E-04	0.04661802

PB.22186(chr23:18780103-18809679)	86.3695644	18	7.85E-04	0.04741865
ENSBIXG00005030608	38.3654609	18	7.97E-04	0.04788233
ENSBIXG00005006560	41.3456363	18	8.33E-04	0.04926397
ENSBIXG00005010688	30.0872692	18	8.29E-04	0.04926397
ENSBIXG00005023220	36.6775078	18	8.25E-04	0.04926397

Figures

Figure S1. An overview of the analysis pipeline used to generate full-length transcript annotations in Brahman and Angus liver samples. In expression correlation, pair-wise correlation plots were made between the outputs for the same colors (green and grey). Similarly, the comparisons were made for the same colors (purple, yellow, pink and orange) in sequencing technology comparison.

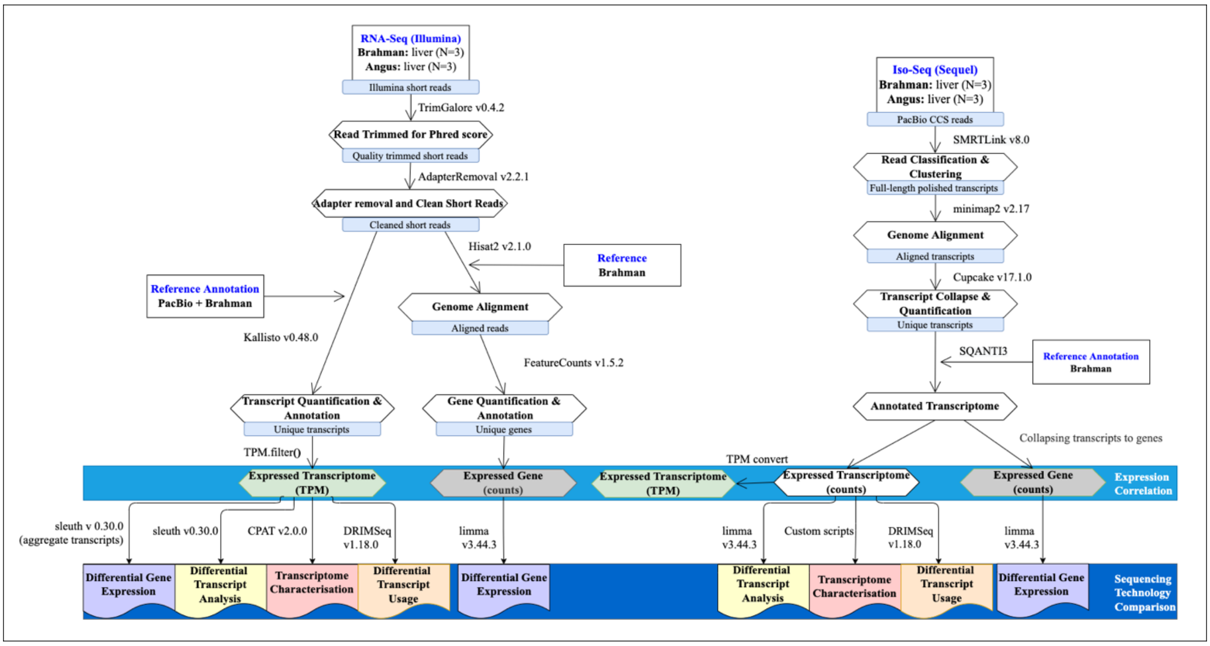

Figure S2. Rarefaction curve of Iso-Seq data for gene and transcript level.

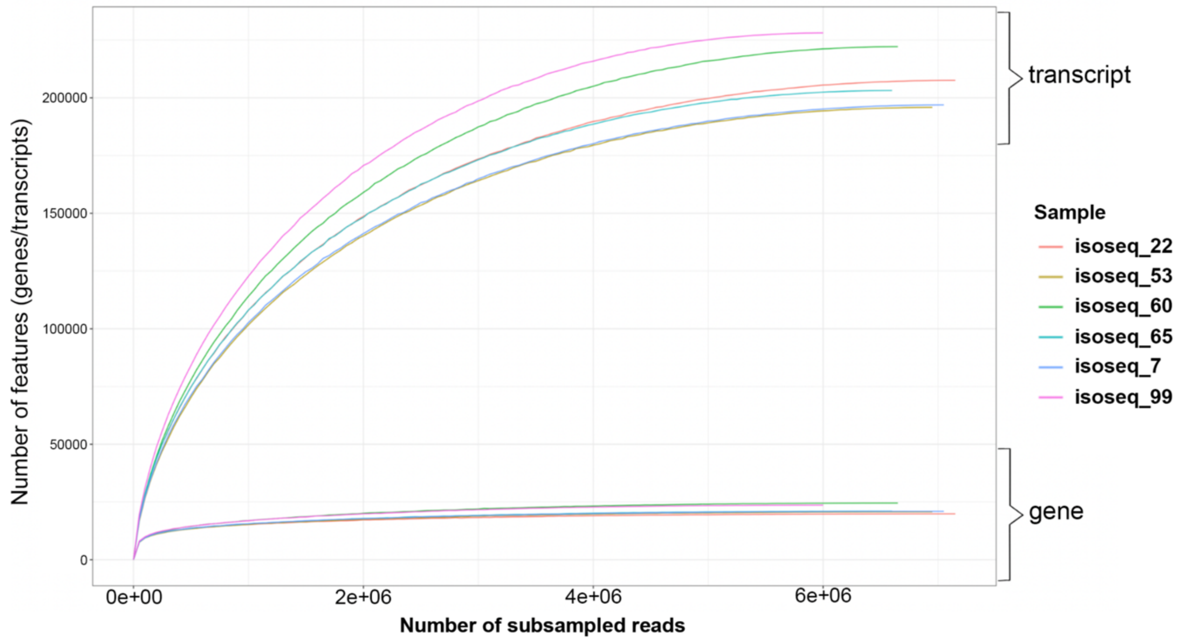

Figure S3. Number of isoforms per gene identified by Iso-Seq. (A) Number of isoforms per gene separated by known and novel genes. (B) The distribution of number of isoforms per gene.

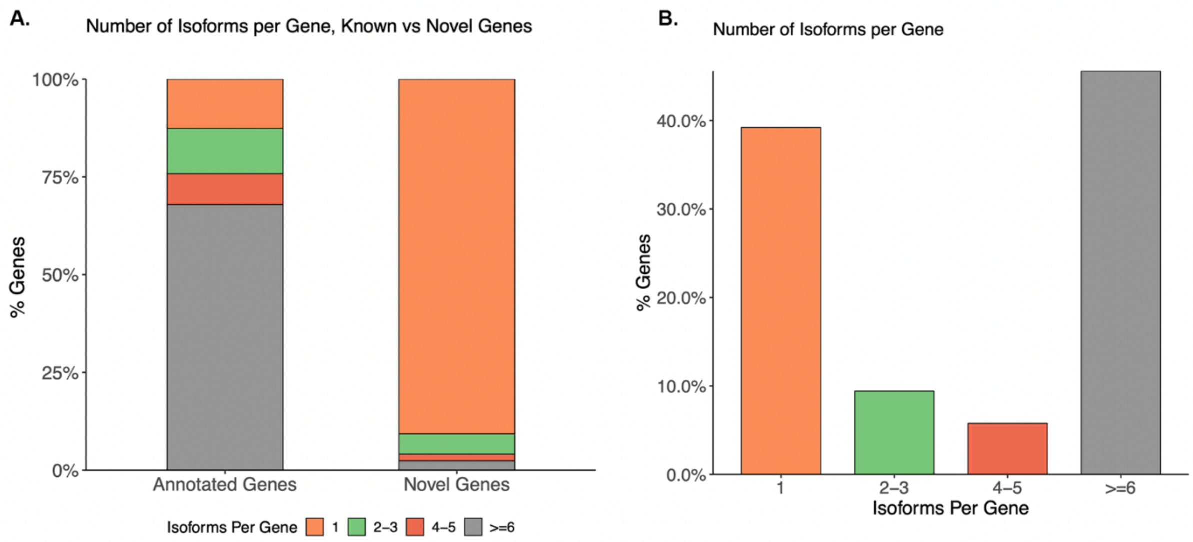

Figure S4. Transcript distribution across structural categories and the distribution of transcript length identified by SQANTI3. Full Splice Match (FSM), Incomplete Splice Match (ISM), Novel in Catalog (NIC), and Novel Not in Catalog (NNC).

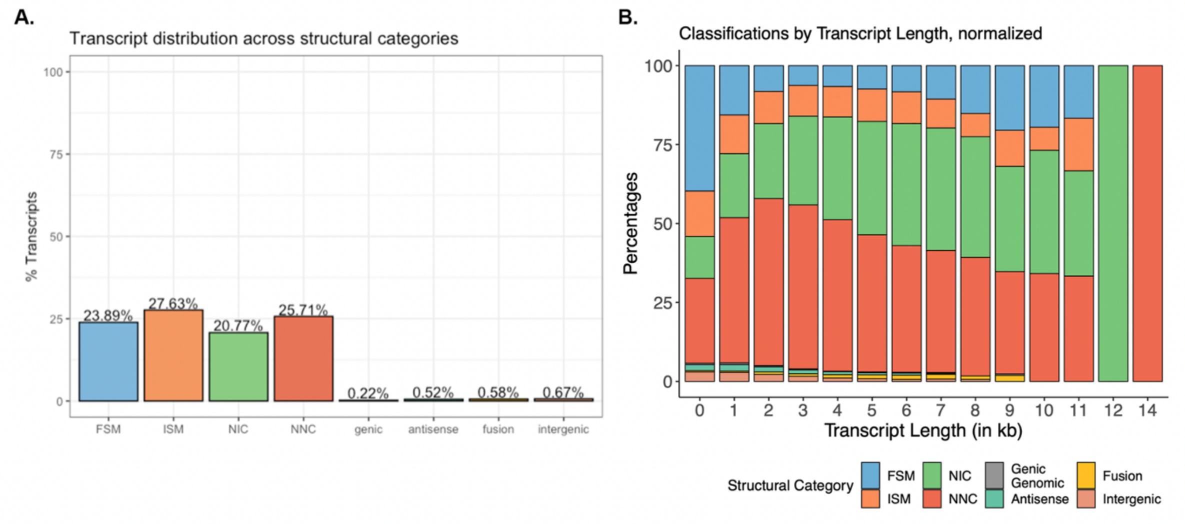

Figure S5. The percentage of overlapping genes in DEGs. The percentage of overlapping DEGs identified by RNA-seq only, the percentage of overlapping DEGs identified by Iso-Seq only and the percentage of overlapping DEGs identified by both are presented.

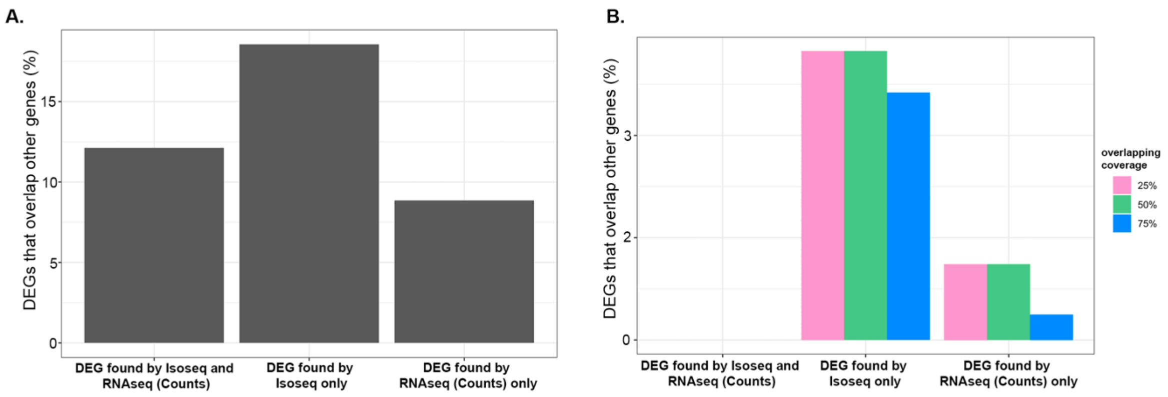

Figure S6. Proportion of differential transcript usage for gene encoding Ig-like domain-containing protein. This transcript usage result was done with RNA-seq data.

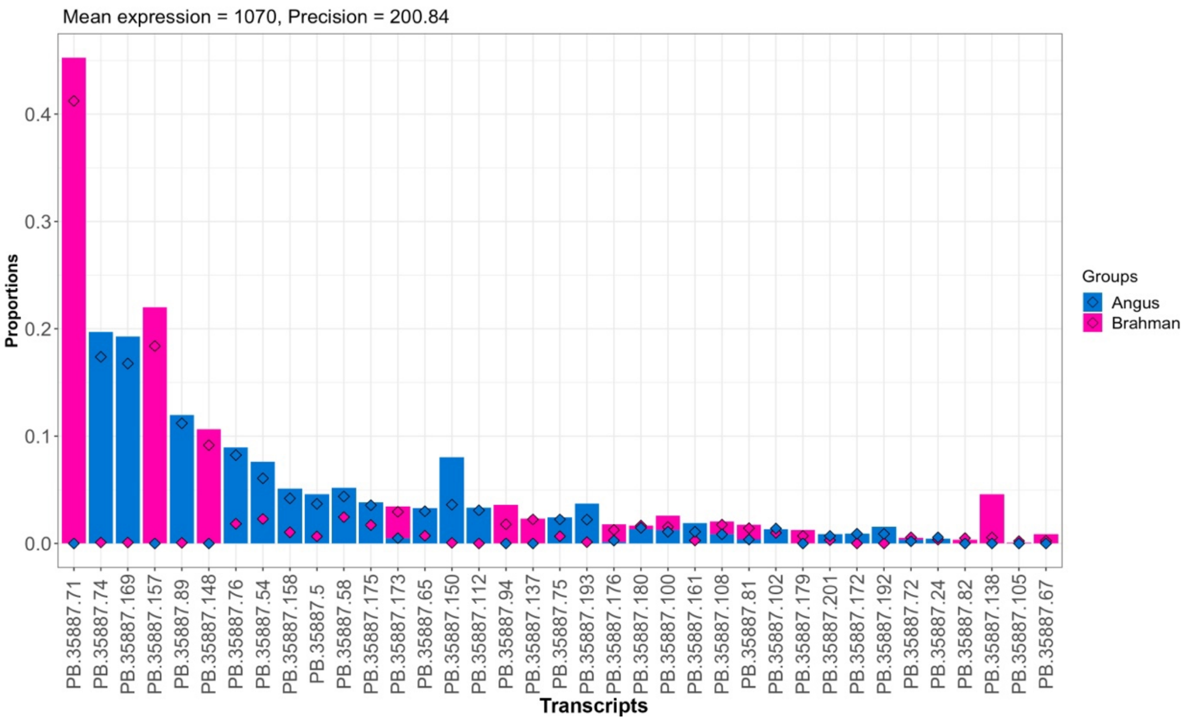

Figure S7. The GO pathway analysis results on high confidence DEGs (A) and DTUs (B).

A.

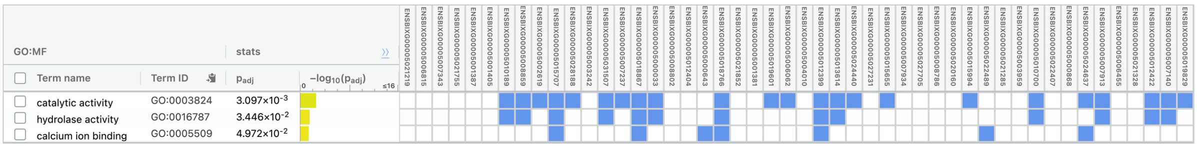

B.

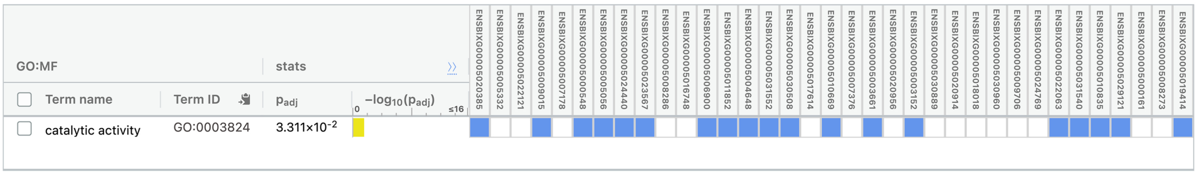

Supplement: Supplementary file 1 — Additional file 1: Table S1. Significant DEGs (p-value <0.05) identified from Iso-Seq dataset using limma linear model. Table S2. DEGs identified by RNA-seq technology using RfeatureCounts and limma linear model with p-value <0.05. Table S3. DEGs identified by RNA-seq technology using sleuth with p-value <0.05. Table S4. DETs identified by Iso-Seq technology using limma Linear model with p-value <0.05. Table S5. DETs identified by RNA-Seq technology using sleuth with p-value <0.05. Table S6. The p-values from Wilcoxon test of the significance of different multi-mapping levels between three groups (DEGs identified by RNA-seq, DEGs identified by Iso-Seq, DEGs identified by both). Table S7. The significant DTUs (p < 0.05) identified using Iso-Seq technology. Table S8. The significant DTUs (p < 0.05) identified using Iso-Seq technology. Figure S1. An overview of the analysis pipeline used to generate full-length transcript annotations in Brahman and Angus liver samples. In expression correlation, pair-wise correlation plots were made between the outputs for the same colors (green and grey). Similarly, the comparisons were made for the same colors (purple, yellow, pink and orange) in sequencing technology comparison. Figure S2. Rarefaction curve of Iso-Seq data for gene and transcript level. Figure S3. Number of isoforms per gene identified by Iso-Seq. (A) Number of isoforms per gene separated by known and novel genes. (B) The distribution of number of isoforms per gene. Figure S4. Transcript distribution across structural categories and the distribution of transcript length identified by SQANTI3. Full Splice Match (FSM), Incomplete Splice Match (ISM), Novel in Catalog (NIC), and Novel Not in Catalog (NNC). Figure S5. The percentage of overlapping genes in DEGs. The percentage of overlapping DEGs identified by RNA-seq only, the percentage of overlapping DEGs identified by Iso-Seq only and the percentage of overlapping DEGs identified by both are presented. Figure S6. Pro [file 12864_2023_9212_MOESM1_ESM.pdf]
